# Predicting Conversion From Unipolar Depression to Bipolar Disorder and Schizophrenia: A 10-Year Retrospective Cohort Study on 12,182 Inpatients

**DOI:** 10.1155/da/4048082

**Published:** 2025-02-20

**Authors:** Ting Zhu, Ran Kou, Di Mu, Yao Hu, Cui Yuan, Minlan Yuan, Li Luo, Wei Zhang

**Affiliations:** ^1^West China Biomedical Big Data Center, West China Hospital, Sichuan University, Chengdu, China; ^2^Med-X Center for Informatics, Sichuan University, Chengdu, China; ^3^Business School, Sichuan University, Chengdu, China; ^4^Centre for Epidemiology and Biostatistics, Melbourne School of Population and Global Health, The University of Melbourne, Parkville 3053, Victoria, Australia; ^5^Sichuan Provincial Center for Mental Health, The Center of Psychosomatic Medicine of Sichuan Provincial People's Hospital, University of Electronic Science and Technology of China, Chengdu, China; ^6^Mental Health Center of West China Hospital, Sichuan University, Chengdu, China

**Keywords:** bipolar disorder, disease conversion, machine learning (ML), risk prediction, schizophrenia, unipolar depression

## Abstract

**Background:** The initial stages of bipolar disorder (BD) and schizophrenia (SCZ) often exhibit depressive symptoms and syndromes, leading to potential misdiagnosis and treatment for unipolar depression (UD). However, no consensus exists on individualized and time-varying intervenable conversion predictors for both BD and SCZ.

**Methods:** This study examined the rate of true conversion from UD to BD and SCZ, considering factors such as sex, family history of mental illness, psychotic features, recurrent depression, and treatment patterns. The objective was to develop predictive models for short-, medium-, and long-term risk stratification for BD/SCZ conversion. Data were extracted from electronic medical records (EMRs) between January 2009 and December 2020 in a large academic medical center-based health system in China. Participants included 12,182 depressive inpatients without previous or comorbid diagnoses of BD and SCZ. The outcome measure was a subsequent admission record with a diagnostic code reflective of BD or SCZ. Four machine-learning algorithms using sociodemographic, clinical, laboratory, vital signs, symptoms, and treatment features were applied to predict this outcome. Explainable methodologies, specifically SHapley Additive exPlanations (SHAP) and Break Down, were employed to analyze the contribution of each individual feature.

**Results:** Among 12,182 individuals, 344 (2.82%) and 64 (0.53%) received a subsequent diagnosis of BD and SCZ, respectively. Higher risk factors for BD progression included being female, having severe depression, being prescribed mood stabilizers, *β* receptor blockers (e.g., metoprolol tartrate and propranolol hydrochloride), and antipsychotics (e.g., sulpiride and quetiapine). Higher risk factors for SCZ progression included being male, exhibiting psychotic symptoms, being prescribed antipsychotics (e.g., risperidone and sulpiride), antiside effects drugs (e.g., trihexyphenidyl and hemp seed pill), and undergoing psychotherapy. Individuals with a family history of mental illness were particularly susceptible to conversion to BD and SCZ.

**Conclusions:** The model's performance on the test dataset declined over time, with area under the curve (AUC) values for predicting BD conversion decreasing from 0.771 in 1 year to 0.749 in 2 years and 0.733 in 7 years, and for SCZ conversion, from 0.866 in 1 year to 0.829 in 3 years and 0.752 in 7 years. A key finding is that individuals with refractory (particularly psychotic) UD had an elevated risk of transitioning to BD and SCZ, with social-demographic factors, lifestyle behaviors, vital signs, and blood markers becoming significant risk factors over follow-up. Upon further validation, these models could provide clinicians with dynamic information regarding a patient's risk of disease conversion.

## 1. Introduction

Unipolar depression (UD), bipolar disorder (BD), and schizophrenia (SCZ) [1–4] are globally recognized as significant contributors to functional and vocational disability [5], as well as suicide-related fatalities [6]. In the early stages of both BD and psychotic disorders, many individuals primarily exhibit depressive symptoms and syndromes. This often hinders the early identification of BD and SCZ, particularly when an individual initially presented with a phenotype resembling UD but is later diagnosed with BD/SCZ. This early misdiagnosis and subsequent treatment for UD can result in detrimental outcomes for patients, including inappropriate antidepressant prescriptions, transition to manic episodes, and a worsened prognosis, all of which contribute to increased healthcare costs [7, 8]. Furthermore, the prescription of antidepressants has been linked to treatment resistance and increased suicidality [9]. Diagnostic changes may naturally occur as a reflection of illness progression, particularly in younger patients with evolving conditions, or potentially as a result of provocation, such as antidepressant induction [10]. There is a pressing need to identify the clinical characteristics of BD/SCZ converters at the first onset of depression, which could guide both patients and clinicians toward early diagnosis and personalized treatment strategies. Consequently, the stratification of conversion risk, especially early in the illness course, has garnered significant interest from both clinicians and researchers [11].

The transition from UD to BD or SCZ represents a significant clinical milestone that necessitates alterations in psychopharmacological management, such as the reduction of antidepressant usage and the introduction of a mood-stabilizing agent [12]. Regrettably, the transition from UD to BD or SCZ often remains unnoticed or is delayed, leading to extended periods of untreated BD/SCZ [13]. On average, individuals with BD encounter a gap of nearly 6 years between the onset of mood symptoms and the commencement of management [14], with one study suggesting that a third of these individuals endured a decade or longer before receiving a diagnosis [15]. Numerous efforts have been made to identify risk factors for this disease conversion, with the aim of reducing the duration of untreated illness and facilitating early detection. Reported predictors of the development of BD in those initially presenting with UD include: female, family history of BD [16, 17]; younger age at onset [18–20]; atypical depressive symptoms such as hypersomnia and increased appetite or weight [21–23]; greater number of depressive episodes [24, 25]; cyclothymic, hyperthymic or irritable temperament [26–28]; psychotic features [29]; suicidal behavior [30]; mood-switching during antidepressant treatment [31, 32]; and substance abuse [33, 34]. Severe depression, particularly psychotic depression, has been identified as the most consistent predictor for conversion from UD to SCZ [35]. However, these factors have typically been reported in separate studies that generally rely on small, selected cohorts, and widely accepted predictors have demonstrated poor performance [36]. Given the distinct prognosis and treatment strategies for BD and SCZ, understanding the frequency of the UD patients who switch to BD and SCZ, as well as the factors predicting these conversions, is crucial for identifying at-risk patients within different follow-up temporal windows.

Kessing et al.'s [37] meta-analysis demonstrated that across 11 studies, the cumulative risk for conversion from UD to BD over a 10-year period was 12.9%. Conversely, Ratheesh et al.'s [29] meta-analysis highlighted significant variation in conversion risk findings across studies, with inconsistent evidence regarding predictors. An analysis of the National Institute of Mental Health Collaborative Depression Study (CDS) at a 10-year follow-up indicated that ~10% of those with major depression developed BD [38]. Another study investigating the transition from UD to SCZ demonstrated that the cumulative incidence of conversion over an 18.5-year period was over 8% in men and nearly 6% in women [39]. A study investigating the transition from UD to SCZ reported a 15-year cumulative incidence of conversion at 2.5% [35]. The conversion rate from UD to BD or SCZ reported in the literature varies significantly, depending on the research object.

Regrettably, there is a dearth of knowledge regarding the ratio of transition to BD or SCZ and the correlation with the use of psychotropics and other commonly prescribed drugs for patients with UD in China due to the absence of large nationwide studies. Previous literature is subject to several limitations. First, most studies have a relatively small sample size (i.e., fewer than 1000 participants), which restricts the generalizability of their findings. Second, the majority of these studies employ cross-sectional designs, leaving a gap in knowledge about the long-term transition of BD/SCZ in patients of UD. Lastly, there is a lack of data on the association between certain drugs (e.g., citalopram, venlafaxine, and amitriptyline) or physical therapy approaches (e.g., modified electroconvulsive [ME] therapy, biofeedback therapy, transcranial magnetic stimulation, and electroencephalographic [EEG] biofeedback therapy) and the likelihood of BD/SCZ in individuals with UD. Furthermore, few studies have utilized machine learning (ML) approaches to predict disease conversion among UD patients despite the widespread application of artificial intelligence technology in addressing high-dimensional medical prediction problems with good performance. Pradier et al. [36] developed a diagnostic change prediction model among 67,807 UD individuals, achieving an area under the curve (AUC) of 0.76 (0.73–0.80) based on information extracted from electronic medical record (EMR). Nestsiarovich et al. [40] constructed a predictive model of major depressive disorder (MDD) to BD conversion within a 1-year period based on a multinational network of patient databases, with AUC varying from 0.633 to 0.745 (mean = 0.689). However, these studies either only considered the association of the nine most prescribed antidepressants with subsequent transition from UD to BD or only included sociodemographic and clinical predictors. Emerging evidence suggests that the uric acid (UA) level is a great help in predicting BD transition in UD patients [41]. As of now, no definitive laboratory or vital sign markers have been identified to detect the transition between UD and BD [42].

In response to this gap in knowledge, our aim was to construct predictive models for short-, medium-, and long-term risk stratification for BD/SCZ conversion following the prescription of commonly used drugs, physiotherapies, and psychotherapies to inpatients initially diagnosed with UD, relying solely on information available in EMR. The principal aim of this study was to (i) ascertain the cumulative incidence of transition from UD to BD or SCZ; (ii) determine the clinical, laboratory, vital signs, symptoms, and treatment characteristics of the converters and nonconverters; and (iii) develop straightforward, clinically useful classifiers that provide high-quality risk estimation and identify individual risk factors within different follow-up temporal windows, thereby assisting practitioners in early recognition of BD/SCZ among patients presenting with a phenotype resembling UD.

## 2. Materials and Methods

### 2.1. Study Design and Data

The data were sourced from the EMR system of West China Hospital (WCH) of Sichuan University, a prestigious first-class tertiary public hospital in China, ranking second nationally. With a capacity of 4300 beds and more than 10,000 medical staff, WCH's psychiatric department stands as one of the four national mental health centers in China, catering to a vast number of patients with mental health disorders nationwide. Annually, it records over 300,000 outpatient visits and discharges more than 6000 patients. These patients hail from over 33 provinces, municipalities, and autonomous regions across China, with notable concentrations from Sichuan, Tibet, Chongqing, Guizhou, Yunnan, Gansu, Shandong, and other regions. As such, WCH exemplifies clinical practice for Chinese mental health patients. Consequently, the study sample is highly representative of the Chinese population. In 2009, an integrated EMR system was universally adopted across all departments in WCH, marking the starting point for data extraction in our study. The data for the study primarily comprised sociodemographic information collected upon admission, laboratory results, medications, vital signs, diagnoses, length of stay, procedure codes, past medical history, current medical history, physician's medical advice, chief complaints, and discharge summary.

### 2.2. Study Setting and Sample Selection

According to the research data, we established a cohort of UD inpatients treated at WCH (according to the International Classification of Disease, Tenth Revision [ICD-10, Clinical Modification Codes F32 and F33]) between January 2009 and December 2020. The ICD-10 code system, regarded as the gold standard for diagnostic categorization, ensures the accuracy of diagnoses through rigorous evaluations conducted by highly trained psychiatrists. These evaluations utilize both structured instruments, such as the Composite International Diagnostic Interview (CIDI), the Diagnostic Interview Schedule (DIS), and the Mini International Neuropsychiatric Interview (MINI), as well as semi-structured diagnostic interviews, exemplified by the Structured Clinical Interview for DSM (SCID) and Schedules for Clinical Assessment in Neuropsychiatry (SCAN) [43]. These comprehensive evaluations are performed at admission and undergo subsequent confirmation and, at times, revision through repeated assessments during the hospitalization period. These conditions make it the optimal choice to improve the accuracy of diagnosis and data quality. Therefore, the discharge diagnosis codes represent all the diseases of each patient during their hospitalization.

**Figure 1:**
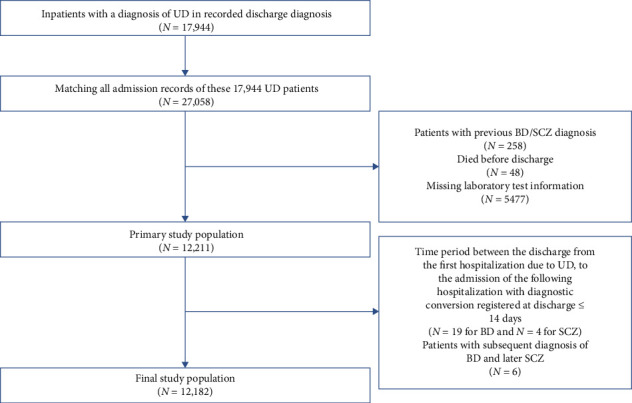
Flowchart yielding the final cohort of 12,182 patients with the first hospitalization due to UD since 2010. BD, bipolar disorder; SCZ, schizophrenia; UD, unipolar depression.

In total, 17,994 patients diagnosed as UD, with a total of 27,058 admission records were extracted. The index admission was defined as the first identifiable hospitalization record associated with the patient's discharge diagnosis of UD. We excluded the following records: (1) patients with previous or comorbid diagnoses of BD, manic/mixed affective states, SCZ, or other nonaffective psychosis; (2) patients who did not survive the UD episode; (3) patient laboratory test information was missing; (4) patients with a time interval of less than 2 weeks between discharge from the initial admission and admission of the subsequent hospitalization with a BD/SCZ diagnosis registered at discharge; (5) patients with subsequent diagnosis of BD and later SCZ. The final count of patients incorporated into the study is illustrated in Figure 1. The index admission records of 12,182 patients were extracted for analysis. Outpatient data were not accessible for this study.

### 2.3. Outcome Definition

This study was primarily designed to predict the transfer from UD to BD within 1 year, 2 years, and 7 years, and to SCZ within 1 year, 3 years, and 7 years. The primary study outcome was the index diagnosis. Conversion outcomes were determined based on the diagnoses of hypomania or mania (F30.x), BD (F31.x), and SCZ (F20.x) according to the ICD-10. The outcome measure was a binary indicator denoting whether a patient had a subsequent hospital admission with the aforementioned outcome diagnoses within different follow-up temporal windows.

A minimum interval of 2 weeks was established between discharge from the first hospitalization due to UD and admission of the subsequent hospitalization with a BD/SCZ diagnosis registered at discharge. This 2-week period was implemented to ensure that the study was examining the true transition from an episode of UD to a subsequent (first) episode of BD/SCZ, thereby avoiding potential misclassification of the index UD diagnosis as an episode of BD/SCZ. Follow-up was stopped when one of these outcome diagnoses occurred or when the follow-up period ended. Consequently, patients with a BD diagnosis conversion within 1 year/2 years/7 years were classified as “cases” (similarly, SCZ diagnosis conversion within 1 year, 3 years, and 7 years), and those who did not convert within different follow-up temporal windows after the index admission were considered as the “control group.” Given the discrepancy in the temporal patterns between the transition from UD to BD and that from UD to SCZ, the three temporal windows were determined by the time point at which the number of cases was divided into thirds, respectively, for BD and SCZ.

### 2.4. Predictors

According to prior studies, the transfer from UD to BD or SCZ was driven by the interaction of a variety of genetical, biological, clinical, psychological, behavioral, pharmacological, social, and environmental factors. A range of covariates were extracted from the original medical records to investigate their influence on the risk of transfer from UD to BD or SCZ. Specifically, (1) basic information: sex, age, marital status, work status; (2) hospitalization information: length of hospital stay (LOS), diagnostic codes (ICD-10 diagnoses, recurrent or single episode UD, comorbidities, etc.), way of leaving the hospital, surgery information; (3) past history encompassing surgical history, trauma history, allergy history, blood transfusion history, family history of mental illness, smoking history, alcohol history, and drug use history; (4) physical examination at admission: pulse, respiration index, body temperature, systolic and diastolic blood pressure; (5) text data (chief complaints, history of present illness, summary of medical records, discharge records); (6) laboratory test data (routine blood, biochemical tests); (7) treatment-related data (prescribed drugs, physiotherapies, and psychotherapies) available at the time of index admission of UD. Through the analysis of the doctors' order dataset, we captured and extracted each patient's treatment patterns from the EMR system. These patterns included medication prescriptions (such as the use of commonly prescribed antidepressants, antipsychotics, anxiolytics, mood stabilizers, anti-side effects drugs, new hypnotics, *β* receptor blockers, hormonal drugs, and Chinese patent medicines), physiotherapy, and psychotherapy patterns.

The fifth item of data requires the technology of natural language processing (NLP). According to our previous work [43], we initially processed patients' chief complaint data by eliminating all punctuation and digits. Text data were segmented into words using the “jieba” in R. We were able to exclude irrelevant words and prevent certain medical terms from being fragmented by incorporating stop words and medical dictionaries. Frequency was employed to identify the informativeness of words in chief complaints. These words were subsequently regarded as features representing the primary symptoms and mood of the patients. The value of each symptom predictor was determined based on whether the patient's chief complaint included the aforementioned keywords, specifically, 1 for inclusion of the specific word and 0 for noninclusion (Supporting Information 1.1).

Regarding the sixth data item, each patient may have undergone multiple laboratory tests in the first hospitalization. We procured the initial recorded laboratory test results during the inpatient stay for all subjects and analyzed all laboratory data. For the laboratory tests, only the most frequently observed result types were incorporated into the ML models. Each numerical or categorical laboratory indicator was treated as a predictor. Any laboratory data recorded after the initial date were excluded from the model.

Finally, 249 predictors were incorporated into the original data pool (details are provided in Table S1).

### 2.5. Prediction Framework

**Figure 2:**
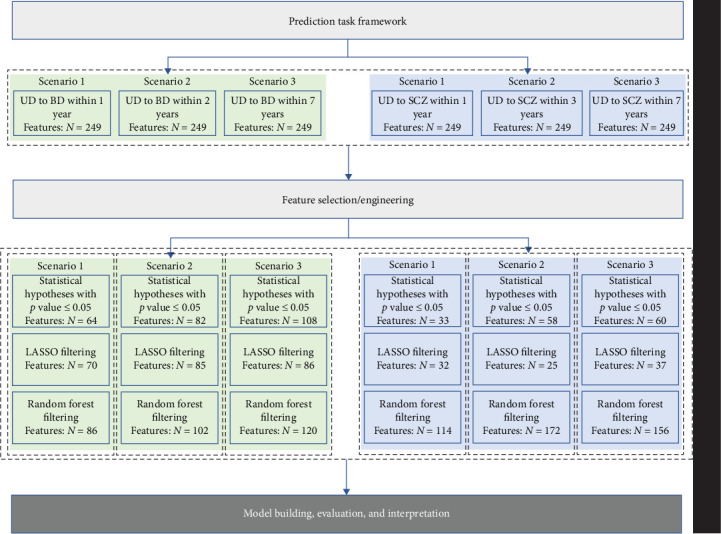
Prediction task framework. BD, bipolar disorder; SCZ, schizophrenia; UD, unipolar depression.

This study aimed to predict the risk of transition from UD to BD within 1, 2, and 7 years and to SCZ within 1, 3, and 7 years, based on the aforementioned predictors available at the time of index admission (refer to Figure 2). The three types of feature filtering methods and ML modeling were performed for each of the six scenarios in Figure 2.

### 2.6. Feature Selection/Engineering

Data preprocessing and ML modeling were carried out in R 3.6.3. The feature filtering phase offers the dual advantages of mitigating overfitting to some extent and facilitating variable selection. Hence, we conducted a comparative analysis among a statistical test method, an inherently interpretable approach, and a method that lacks inherent interpretability. Initially, we compared the case and control (UD-BD converter vs. UD-BD nonconverter and UD-SCZ converter vs. UD-SCZ nonconverter) in terms of all included features. We conducted statistical tests on each predictor and incorporated those with a *p*-value of 0.05 or less into the ML model. Second, we employed the LASSO for feature selection. LASSO is an embedded model that identifies the features contributing most significantly to model accuracy by implementing a shrinking (regularization) process that penalizes the coefficients of the regression features, reducing some of them to zero [44]. We also employed a random forests (RFs) approach, wherein the most commonly used measure of a given variable's importance is the increase in the mean error of a tree within the forest. Furthermore, the Gini impurity function was also employed as a variable importance measure in the RF model. Particularly, LASSO was implemented using the glmnet package (cv.glmnet function to obtain the best parameter lambda), and RF was implemented using the randomForest package (tuneRF function to train the model and importance function to obtain feature importance).

### 2.7. Model Building, Evaluation, and Interpretation

Four standard ML algorithms were implemented to analyze the data obtained by feature filtering methods, and BD, SCZ converters/nonconverters within three follow-up temporal windows as prediction classes, respectively. We evaluated extreme gradient boosting (XGBoost) [45], logistic regression (LR), RF, and AdaBoost. For each scenario, we conducted feature selection, hyperparameter optimization, and fitting of all models within internal cross-validation (CV). Our ML pipeline commenced by randomly dividing the entire dataset into four stratified subsets, each comprising 25% of the UD-BD data and the UD-SCZ data. We employed a grid search on the training set to identify the parameter combination that yielded the maximum AUC value.

In the case of LR, tuned model parameters were grid searched in the range [0, 1] with 11 values for alpha and in the range [0.01, 1] with 100 values for lambda using the expand.grid function. Tuned model parameters of the XGBoost model, including the number of optimization rounds (100–1000 with 10 values), maximum tree depth (2–10 with 9 values), and the learning rate (0.05–1 with 20 values), were grid searched using the expand.grid function. The RF model considers two hyperparameters in the training set: the number of trees (ntree, in the range [200, 1500] with 14 values) and the count of variables tried in each split (mtry, in the range [2, 10] with 9 values). The Adaboost model considers two hyperparameters in the training set: the number of basic classifiers (mfinal, in the range [5, 100] with 20 values) and the maximum tree depth (maxdepth, in the range [3, 10] with 8 values). Training of the four algorithms was based on a 10-fold CV repeated three times.

Model evaluation indicators such as the AUC, sensitivity, specificity, PPV, and NPV (the positive and negative predicted values) in the held-out testing set, averaged over four different splits of the entire data, were utilized. Youden's index was employed to determine the cutoff value for optimal classification, balancing sensitivity and specificity. The 90% confidence intervals (CIs) were estimated for the AUC of prediction performance. The statistical significance of the AUC obtained by different feature filtering methods for each algorithm was calculated using the DeLong test.

Moreover, we employed interpretable ML methods, including SHapley Additive exPlanations (SHAP) [46] and Break Down [47], to the predictive models to derive characteristic explanations of the prediction results. SHAP is a classic interpretable ML method. This method is inspired by cooperative game theory, and the impact of each feature on a specific prediction is expressed using Shapley values [48]. Break Down is used to identify the contribution of variables to the model's predicted results [47]. Our previous research further elaborates on the details of these interpretable ML methods [43].

## 3. Results

### 3.1. Sample Characteristics

Table 1 presents the clinical and sociodemographic characteristics of the patients. The median and interquartile range of patient age was 46 [28, 61], and a majority of the patients were female (65.9%). A diagnosis of single depression was given to 93.3% of the patients at their initial hospitalization, while the remaining patients were diagnosed with recurrent depression. In addition to the primary UD diagnosis, patients' supplementary disease diagnoses were recorded: 38.5% of the patients received at least one other psychiatric disease diagnosis, 22.1% of the patients had at least one endocrine disease, 9.4% of the patients had at least one nervous disease, 19.5% of the patients had at least one digestive disease, 24% of the patients had at least one circulatory disease, 12.3% of the patients had at least one respiratory disease, and 4.9% of the patients had at least one cancer. A family history of mental illness was present in 5.4% of the patients. Considering lifestyle behaviors, 28% of the patients had a history of smoking, 22.2% had a history of drinking, and 19.1% had a history of medication use. For chief complaints, 4.8% of the patients reported psychotic symptoms (like persecutory delusion, suspicion, sensitive, auditory hallucination, tracked, followed, under observation), 4.9% presented suicidal ideation or behavior, 7.2% reported provoke, 18.3% had physical discomfort, 33.1% had symptoms of poor sleeping, etc. Table S2, offers a comprehensive comparison of all characteristics between individuals who converted to BD/SCZ and those who did not.

### 3.2. Conversions to BD/SCZ

**Figure 3:**
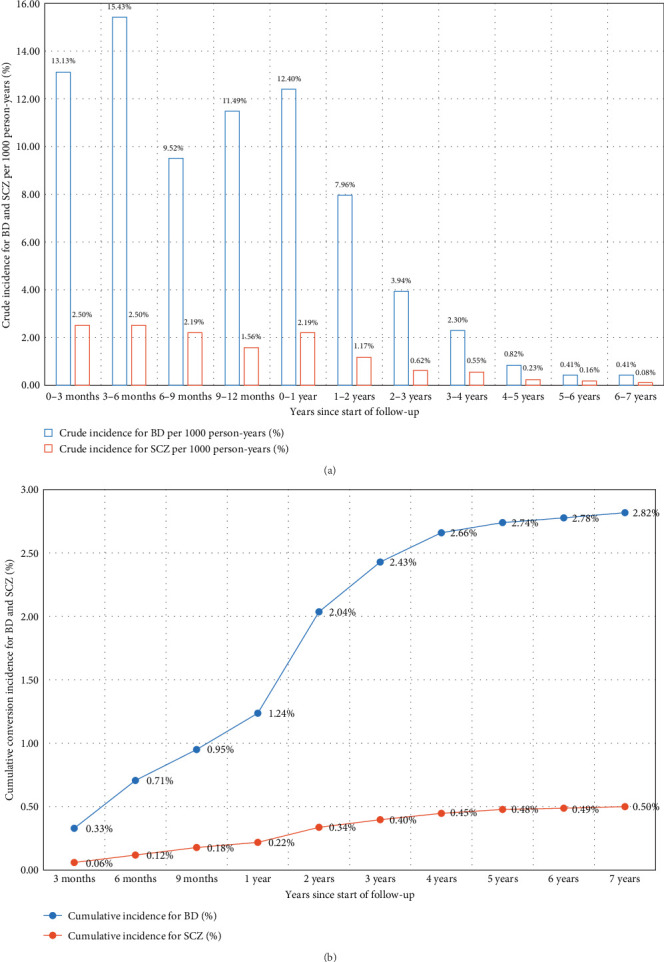
Cumulative conversion incidences and yearly crude incidence rate for the conversion from UD to BD and SCZ. BD, bipolar disorder; SCZ, schizophrenia; UD, unipolar depression.

The sample was observed for a total of 56,810 person-years. The average duration of follow-up was 4.66 years, with a median duration of 4.08 years. In summary, over the 10-year follow-up period, the probability of transitioning from UD to BD was 2.82%, and the probability of transitioning from UD to SCZ was 0.53% (Figure 3). The highest yearly crude incidence rate for BD occurred within the first year of follow-up (12.4 per 1000 person-years); specifically, the crude incidence rate at 0–3, 3−6, 6–9, and 9–12 months since the start of follow-up was 13.13, 15.43, 9.52, and 11.49 per 1000 person-years, respectively. For UD to SCZ conversion, the highest yearly crude incidence rate also emerged within the first year of follow-up (Figure 3). The yearly crude incidence rate progressively declined after the first year and reached zero person-years from the eighth year onward.

Previous research has shown that factors such as sex, family history of mental illness, presence of psychotic features, depression relapse, and treatment modalities can predict the transition from UD to BD and SCZ. From the results of Table 1 and Table S2, we also found that the converter group and nonconverter group presented significant differences among these factors, both for UD to BD and UD to SCZ. Therefore, cumulative conversion incidence was compared between different subgroups with respect to these factors. Rates of transition from UD to BD were substantially higher for females, while rates of transition from UD to SCZ were lower for females compared to males (Figure S1). Transition rates from UD to SCZ were notably higher for patients with a family history of mental illness, and transition rates from UD to BD were marginally higher for patients with a family history of mental illness compared to patients without such a history (Figure S2). Patients with recurrent depression had higher transition rates from UD to BD compared to patients with single-episode depression, while patients with psychotic symptoms had substantially higher transition rates from UD to SCZ compared to patients without psychotic symptoms (Figures S3 and S4).

**Figure 4:**
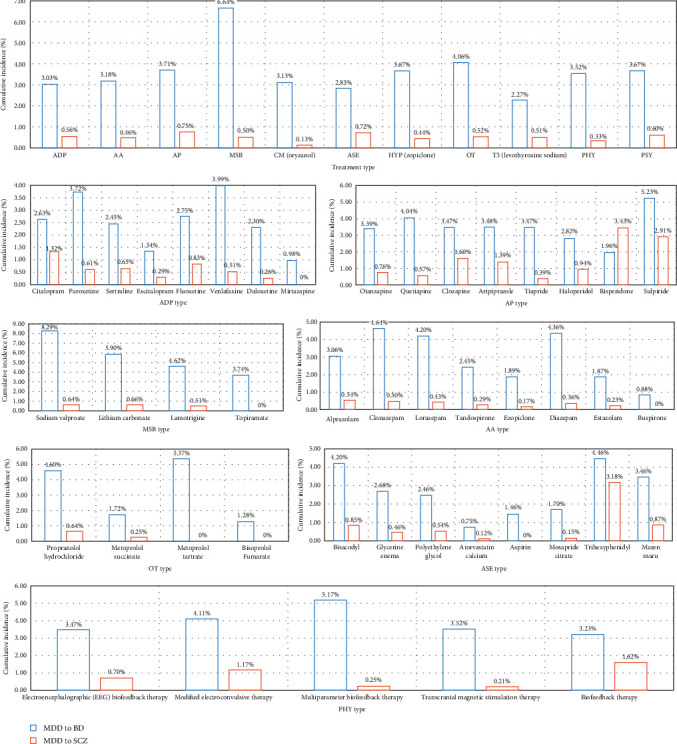
Cumulative conversion incidences for the conversion from UD to BD and SCZ stratified by specific drugs and physiotherapies. BD, bipolar disorder; SCZ, schizophrenia; UD, unipolar depression.

Rates of transition were significantly different across drugs, physiotherapy, and psychotherapy (Figure 4). Generally, patients prescribed mood stabilizers had the highest transition rate (6.64%) from UD to BD, followed by *β* receptor blockers (4.06%) and antipsychotics (3.71%). For rates of transition from UD to SCZ, patients prescribed antipsychotics (0.75%), anti-side effects drugs (0.72%), and psychotherapy (0.6%) had a higher risk than other treatment types. In Figure 4, rates of transition across each specific drug and physiotherapy were compared. For antidepressants, patients prescribed venlafaxine and paroxetine had higher transition rates from UD to BD, and patients prescribed citalopram and fluoxetine had higher transition rates from UD to SCZ. For antipsychotics, patients prescribed sulpiride and quetiapine had higher transition rates from UD to BD, and patients prescribed risperidone and sulpiride had higher transition rates from UD to SCZ. For mood stabilizers, sodium valproate and lithium carbonate prescribed to patients had higher rates of transition. Clonazepam, diazepam, and lorazepam are three anxiolytics with the highest rates of transition from UD to BD. For *β* receptor blockers, metoprolol tartrate and propranolol hydrochloride are two drugs with the highest rates of transition from UD to BD. Trihexyphenidyl and bisacodyl are two anti-side effects drugs affected most for the transition from UD to BD.

### 3.3. Prediction Performance and Predictors of Conversion to BD and SCZ

**Figure 5:**
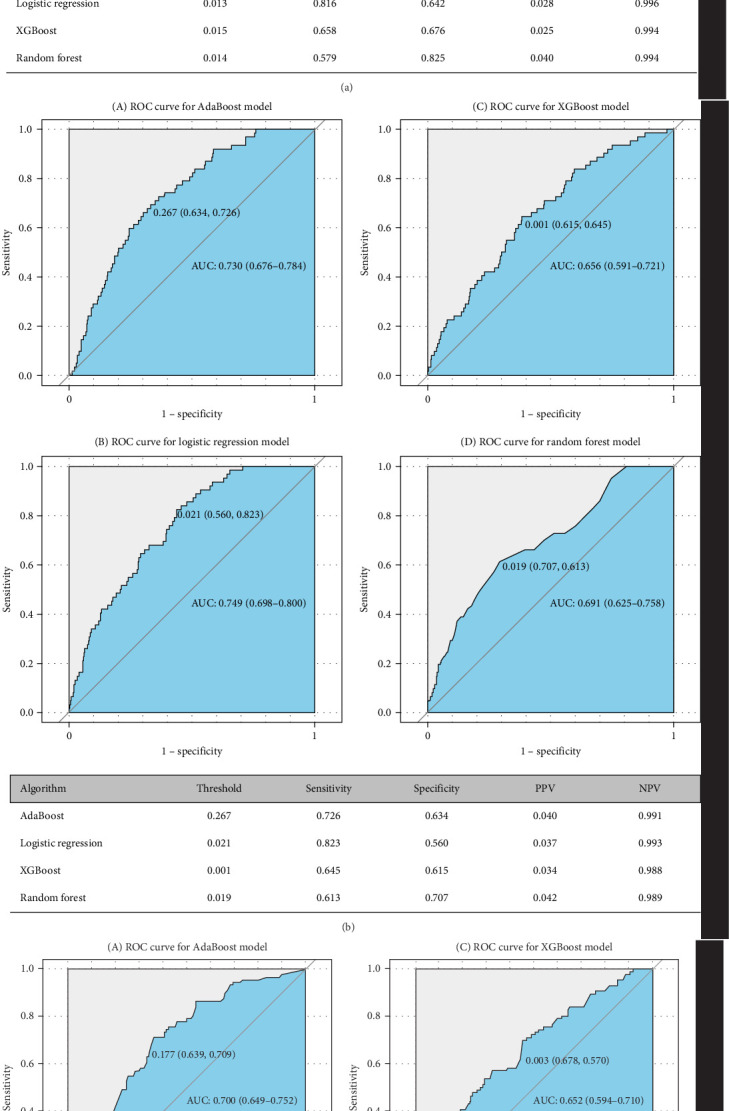
The performance of prediction models for the conversion from UD to BD. The area under the receiver operating characteristic (AUC) ROC curve. The tables showed the internal validation performance (i.e., sensitivity, specificity, positive predictive value [PPV], and negative predictive value [NPV]) of prediction models. (A) Using the statistical test method filtered features to predict conversion to BD within 1 year. (B) Using the statistical test method filtered features to predict conversion to BD within 2 years. (C) Using the statistical test method filtered features to predict conversion to BD within 7 years. BD, bipolar disorder; UD, unipolar depression.

**Figure 6:**
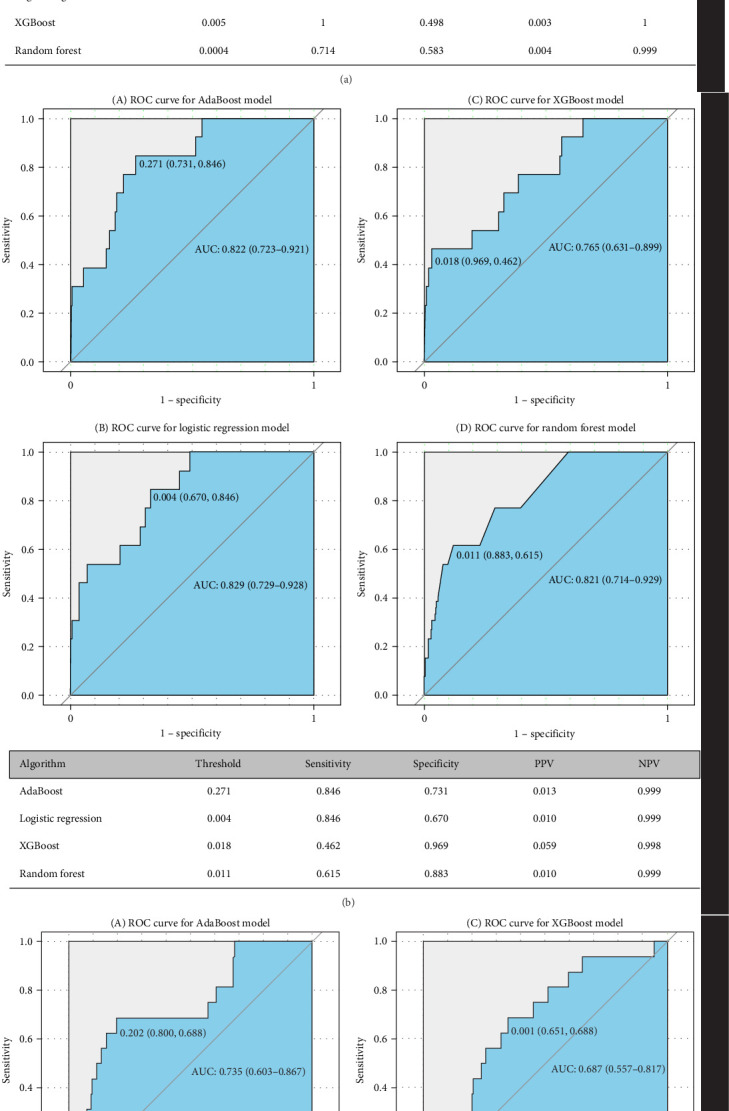
The performance of prediction models for the conversion from UD to SCZ. The area under the receiver operating characteristic (AUC) ROC curve. The tables showed the internal validation performance (i.e., sensitivity, specificity, positive predictive value [PPV], and negative predictive value [NPV]) of prediction models. (A) Using the statistical test method filtered features to predict conversion to SCZ within 1 year. (B) Using the statistical test method filtered features to predict conversion to SCZ within 3 years. (C) Using the statistical test method filtered features to predict conversion to SCZ within 7 years. SCZ, schizophrenia; UD, unipolar depression.

Figures 5 and 6 report model discrimination using the statistical test-filtered features to predict conversion from UD to BD and SCZ within different follow-up temporal windows. For predicting conversion from UD to BD, the best internally validated AUC was 0.771 (90% CI 0.700–0.842) within 1 year achieved by RF, 0.749 (90% CI 0.698–0.800) within 2 years, and 0.733 (90% CI 0.685–0.780) within 7 years achieved by LR. For predicting conversion from UD to SCZ, the best internal validated AUC was 0.866 (90% CI 0.775–0.957) within 1 year, 0.829 (90% CI 0.729–0.928) within 3 years, and 0.752 (90% CI 0.630–0.875) within 7 years achieved by LR. Discrimination achieved by the other two feature filtering methods (LASSO and RF) were illustrated in Supporting information (Figures S5–S8). Among the four ML classifiers evaluated, XGBoost, RF, and AdaBoost did not yield higher AUC values compared to LR. Additionally, the statistical test method employed for filtering important features demonstrated the most effective prediction performance.

**Figure 7:**
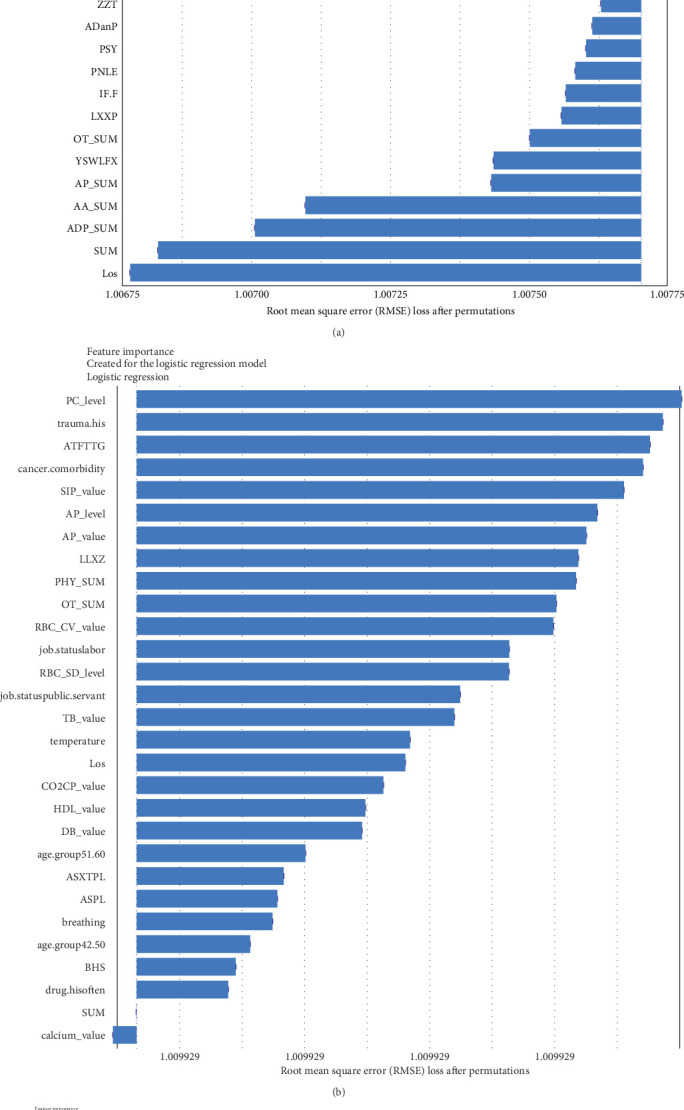
The comparison of the top 30 features to predict conversion from UD to BD within different temporal windows. The blue bar represents the relatively importance of these features. The table shows the ranking of the top 20 features. (The features marked by red indicating they are important in the prediction models within three different time windows. The features marked by yellow indicating they are important in the prediction models within 1 year and within 2 years. The features marked by blue indicating they are important in the prediction models within 1 year and within 7 years. The features marked by orange indicating they are important in the prediction models within 2 years and within 7 years.) The detailed information of the included features is shown in Table S1. (A) The importance of top 30 features to predict conversion to BD within 1 year. (B) The importance of top 30 features to predict conversion to BD within 2 years. (C) The importance of top 30 features to predict conversion to BD within 7 years. BD, bipolar disorder; UD, unipolar depression.

Regarding the importance of features measured using the Break Down method for short-term stratification of risk for BD conversion from UD (Figure 7), length of stay of this hospitalization treatment for UD, sum of prescribed medical orders, sum of prescribed antidepressants, sum of prescribed anxiolytics, sum of prescribed antipsychotics, number of prescribed venlafaxine hydrochloride sustained-release capsules, sum of prescribed *β* receptor blocker orders, number of prescribed clonazepam tablets, whether the patient is admitted to a psychiatric ward, number of prescribed propranolol hydrochloride tablets, etc., were top ranked in predictive models within 1 year. It is noteworthy that markers of greater illness severity, such as length of stay and a higher number of prescribed medications, were strongly associated with the short-term risk of transition to BD. Notable differences were observed with respect to the medium- and long-term stratification of risk for BD transition. Particularly, some features of lifestyle (e.g., history of frequent medication use), vital signs (e.g., breathing and body temperature), and laboratory blood-based markers (e.g., routine biochemical examination_Calcium, level of urea, level of alkaline phosphatase and direct bilirubin, routine blood examination_Red blood cell volume distribution width CV) were top ranked in predictive models within 2 years and within 7 years. In addition, treatment-related factors (e.g., the sum of medical orders, number of orders of ME therapy, benzhexol hydrochloride tablets, escitalopram oxalate tablets, and aspirin enteric-coated tablets) and social factors (age and job status) were also top-ranked.

**Figure 8:**
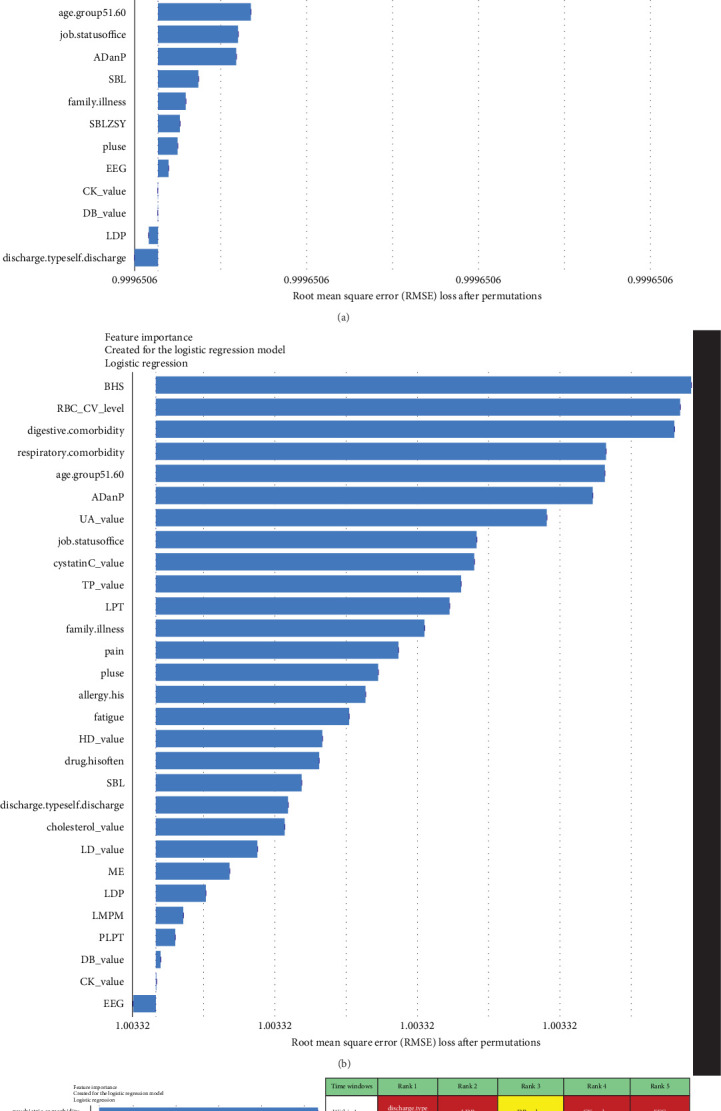
The comparison of the top 30 features to predict conversion from UD to SCZ within different temporal windows. The blue bar represents the relatively importance of these features. The table shows the ranking of the top 20 features. (The features marked by red indicating they are important in the prediction models within three different time windows. The features marked by yellow indicating they are important in the prediction models within 1 year and within 3 years. The features marked by blue indicating they are important in the prediction models within 1 year and within 7 years. The features marked by orange indicating they are important in the prediction models within 3 years and within 7 years.) The detailed information of the included features is shown in Table S1. (A) The importance of top 30 features to predict conversion to SCZ within 1 year. (B) The importance of top 30 features to predict conversion to SCZ within 3 years. (C) The importance of top 30 features to predict conversion to SCZ within 7 years. SCZ, schizophrenia; UD, unipolar depression.

For short-term stratification of risk for SCZ conversion from UD (Figure 8), six features (type of patient discharge is self-discharge, number of orders of clozapine tablets, sulpiride, EEG biofeedback therapy and ME therapy, and routine biochemical examination_Creatine kinase) were top 10 ranked in models within 1 year, 3 years, and 7 years. In addition, features of a family history of mental illness, vital signs (e.g., pulse), laboratory blood-based markers (e.g., routine biochemical examination_Direct bilirubin, Lactate dehydrogenase, and Cholesterol), and treatment-related factors (e.g., number of orders of clomipramine hydrochloride tablets and paliperidone sustained-release tablets) were top-ranked. Interestingly, top-predictive features included markers of poor prognosis for hospitalization (self-discharge), family history of mental illness, medication and physiotherapy indicated for psychosis, changes in vital signs, and biochemical indicators. Detailed information regarding the included features can be found in Table S1.

Furthermore, our model is capable of making predictions for specific patients. We provide illustrations of one patient who converted to BD or SCZ and another patient who did not convert to BD or SCZ in the training set (refer to Figures S9 and S10).

## 4. Discussion

### 4.1. Principal Findings

In this area-based cohort study involving 12,182 patients with UD hospitalized within the health system over a 10-year follow-up period, the rates of progression to BD and SCZ during this period were 2.82% and 0.53%, respectively. These findings align with those from a large national Finnish study by Baryshnikov et al. [35], where the annual crude incidence rates of BD and SCZ were highest during the first year, gradually decreasing after the first year and reaching zero from the eighth year onward. Cumulative conversion incidence was also compared between different subgroups with respect to prior reported risk factors, such as sex, family history of mental illness, psychotic features, recurrent depression, and treatment patterns.

Consistent with prior studies, patients who are female and suffer from recurrent depression were identified as having an increased risk for progression to BD [49], while patients who are male and exhibit psychotic symptoms were identified as having an increased risk for progression to SCZ [16, 35]. Patients who have a family history of mental illness were particularly susceptible to conversion to both BD and SCZ. Moreover, rates of transition were significantly different across psychopharmacological treatment, physiotherapy, and psychotherapy. Generally, patients prescribed mood stabilizers had the highest transition rate from UD to BD, followed by *β* receptor blockers (e.g., metoprolol tartrate and propranolol hydrochloride) and antipsychotics (e.g., sulpiride and quetiapine). This could be attributed to the potential under-diagnosis of BD in individuals with hypomanic or manic symptoms that were either not severe or clinically relevant [50]. Patients with UD who were prescribed antipsychotics (e.g., risperidone and sulpiride), anti-side effects drugs (e.g., trihexyphenidyl and hemp seed pill), and psychotherapy had a higher risk of transition to SCZ than other treatment types.

We constructed ML models to accurately predict the short-, medium-, and long-term risks of illness progression from UD to BD (AUC = 0.733–0.771) and SCZ (AUC = 0.752–0.866). Notably, our models, which utilized easily accessible features, demonstrated good predictive accuracy. The models were notably discriminative in identifying patients at the highest risk for progression to BD and SCZ. However, discrimination somewhat declined with an increasing follow-up temporal window, suggesting that the models generally achieved better prediction accuracy for the period immediately preceding the disease change. Our results align with two previous studies that focused on ML-based diagnosis conversion prediction in individuals with UD using retrospective observational data from two academic medical centers in New England (AUC = 0.73–0.80) [36], and five US databases (AUC = 0.633–0.745) [40]. Our results also identified some well-known risk factors (e.g., sex, younger patient age, severe depression, psychosis, prior mental illness, psychiatry versus other specialty care, medications indicated for psychosis). Another study, which examined 812 mood disorder cases, reported an AUC of 0.72 [51]. However, few studies simultaneously considered the prediction of conversion to BD and SCZ. Additionally, previous investigations have not addressed the comparison of top predictors that matter for short- versus longer-term conversion risk. In this study, our aim was not to catalog all risk factors but to develop applicable predictive models that operate on EMR and to identify time-varying intervenable risk factors.

Benefiting from the enriched data, our established models provide novel insights into factors driving the progression to BD and SCZ within different temporal windows of follow-up. Markers of greater illness severity and more medication prescribed may be strongly associated risk factors for BD transition in the short-term, indicating that short-term risk was more likely to be induced by the severity and treatment of the index depression itself, whereas features of social-demographic (age and job status), lifestyle (e.g., history of frequent medication use), vital signs (e.g., breathing, body temperature), and laboratory blood-based markers (e.g., calcium, urea, alkaline phosphatase, direct bilirubin, red blood cell volume distribution width CV), and treatment-related factors (e.g., sum of medical orders, number of ME therapy orders, benhexol hydrochloride tablets, escitalopram oxalate tablets, and aspirin enteric-coated tablets) were more important for the prediction of medium- and long-term BD conversion risk. Interestingly, self-discharge, clozapine tablets, sulpiride, EEG biofeedback therapy, ME therapy, and Creatine kinase are important for the prediction of SCZ conversion within all temporal windows. Top-predictive risk factors for SCZ conversion included markers of poor prognosis for the index UD hospitalization (self-discharge), family history of mental illness, medication and physiotherapy indicated for psychosis, changes in vital signs, and blood-based biomarkers. Notably, the capacity of certain antidepressants, including tricyclic antidepressants (TCAs) and serotonin–noradrenaline reuptake inhibitors (SNRIs), to induce a switch to mania has been a topic of enduring debate [10]. A key finding of the present study is that individuals with difficult-to-treat UD were more likely to convert to BD and SCZ. Other social-demographic, lifestyle, vital signs, and laboratory blood-based markers became important risk factors with the follow-up period increased. Our findings corroborate the perspective that medication-resistant depression serves as a link between UD and BD [50], and psychosis is strongly associated with UD and SCZ [35].

Consistent with our findings, laboratory blood biomarkers have been documented as beneficial in the diagnosis of BD. Serum UA has excellent prognostic accuracy in predicting conversion to BD in depressed subjects [43]. In addition to UA, several markers have been studied in BD, which can be broadly classified into three categories: neurotrophins, pro-inflammatory cytokines, and oxidative stress markers [37]. Our findings support the hypothesis that routine laboratory biomarkers could be valuable predictors for BD and SCZ conversion risk prediction. Specifically, myocardial enzyme marker (e.g., creatine kinase and lactate dehydrogenase), cardiovascular function marker (e.g., high-density lipoprotein), liver biochemistry marker (e.g., bilirubin), renal function marker (e.g., urea), bone function marker (e.g., alkaline phosphatase), red blood cell markers (e.g., red blood cell volume distribution width CV and SD) and markers of immune-inflammation (e.g., platelet count) were identified as risk factors, with several plausible mechanisms at a molecular level. Evidence suggests that genetic or biological alterations, including immune-inflammatory pathways and oxidative stress pathways, contribute to the risk of co-occurrence of BD and cardiovascular disease [51]. It is recognized that BD patients experience excessive oxidative stress, with reactive species being products of normal physiological functioning and synthesized during mitochondrial cellular respiration [37]. Factors such as hyperactivity and high impulsivity can result in increased levels of cardiac enzymes, cardiovascular function, and immune and inflammatory markers [26]. To date, few efforts have explored the laboratory biomarkers for predicting BD and SCZ [43]. To our knowledge, this study may be the first to investigate the association between all these routine laboratory blood-based biomarkers in UD and conversion risk for BD and SCZ.

## 5. Limitations

This study has several limitations. First, it relies solely on EMR diagnostic codes. Our research subjects hail from a large retrospective observational cohort, where despite rigorous evaluations by psychiatrists aimed at enhancing diagnostic accuracy, the level of participant screening cannot match the stringency of a prospective cohort study design. Even gold standard measures have been found to perform poorly in mood disorders. Research has demonstrated that EMR data can be utilized to identify patients with BD with greater predictive value compared to clinical diagnostic interviews [52]. Likewise, incorporating unstructured clinical text might improve classification rates, so narrative notes on chief complaints were analyzed to capture the symptom features of patients. Second, each patient might seek care at different hospitals; the EMR data extracted from an academic medical center-based health system included incomplete data recording of each patient's all medical visits, leading to missing of diagnosis converters to BD and SCZ. We also recognized that it was not necessarily the first episode of UD in a patient's life captured in our data. Therefore, we cannot state definitively that our models predict the risk of transition to BD and SCZ at the initial-onset stage of UD; rather, detect the individuals at their initial hospitalization for UD and have a subsequent diagnosis of BD or SCZ in the same hospital. Despite all reasonable methodologies employed in training the model, this could introduce a selection bias that restricts the generalizability of the ML models. Validation of our models with more comprehensive data could mitigate some model uncertainties, particularly concerning the stability of identified time-varying risk factors. Given the current lack of data from other medical centers (particularly, laboratory test data and treatment prescription data were difficult to obtain), this study is unable to verify the external validity of our model. Further external validation is a necessary precondition for the application of this risk prediction model within the Chinese health system. Within the health system under study, patients had the option to access other hospitals for healthcare services [45]. This corollary might be adjusted in light of the cumulative incidence of diagnostic change to BD (2.82%) and SCZ (0.53%). Hence, an imbalance in the conversion rate will constrain the PPV of the model. Mislabeling of some true converters as negative cases just because of the inability to obtain their conversion records would tend to decrease the discrimination of predictive models, making the performance estimates provided here likely conservative. This is also why the incidence of evolution from UD to BD and SCZ obtained in this study is lower than that obtained in other studies, such as 12.9% for UD progression to BD [37] and 2.5% for UD progression to SCZ [35]. Finally, while we have rendered the ML models explainable, the percentage of medication-induced disease switching cannot be determined due to the absence of an underlying causal structure in our models.

### 5.1. Strengths

In our study, significant strengths include the utilization of heterogeneous data derived from multiple sources, encompassing individual-level narrative notes on chief complaints, vital signs, laboratory test data, psychopharmacological treatment, physiotherapy, and psychotherapy data from a large regional-based cohort of 12,182 depressive inpatients over a 10-year follow-up. The inclusion of these data suggests that our study may be the first attempt to investigating the association between all routine laboratory blood-based biomarkers and UD and conversion risk for BD and SCZ. Additionally, this study may represent the first investigation into the cumulative transition rate from UD to BD and SCZ within a Chinese public health system. The application of an ML approach, coupled with the use of SHAP and Break Down methods for feature interpretation, allowed us to identify the most informative variables that maximized data efficiency for accurate prediction of disease conversion risk. Furthermore, we enhanced the feasibility of the models by comparing three different feature filtering methods (Figure 2). Our efforts to construct separate models for predicting short-, medium-, and long-term conversion risks provide time-varying intervenable risk factors at both the population and individual levels (Figures 7 and 8 and Figures S9 and S10). We recognize that the aforementioned limitations indicate that the model may not be suitable for clinical application without further validation using external data. We posit that the integration of external data into EMR-based health systems would further enhance the construction of clinical decision-making models.

## 6. Conclusions

In summary, we have developed clinically applicable ML-based models to accurately predict the short-, medium-, and long-term risks of transition from UD to BD and SCZ, utilizing a comprehensive dataset of 12,182 depressive inpatients from a large academic medical center-based health system in Chengdu, China. The robust performance of these models suggests that the integration of explainable ML models with EMR data may enhance prognostic certainty beyond chance level. Model interpretation revealed that top risk factors varied with the follow-up period, suggesting the need for time-varying interventions. The findings on laboratory blood-based markers and treatment-related factors provide directions for further study of disease conversion mechanisms at molecular and psychopharmacological levels.

## Figures and Tables

**Table 1:** Clinical and sociodemographic characteristics of the study population.

Features	Overall *n* = 12,182	BD nonconverter *n* = 11,838	BD converter *n* = 344	*p* Value	SCZ nonconverter *n* = 12,118	SCZ converter *n* = 64	*p* Value
Sex = female (%)	8032 (65.9)	7781 (65.7)	251 (73.0)	**0.006**	7996 (66.0)	36 (56.2)	**0.132**
Age group (%)	—	—	—	**<0.001**	—	—	**<0.001**
0–13	49 (0.4)	48 (0.4)	1 (0.3)	—	48 (0.4)	1 (1.6)	—
13–17	1203 (9.9)	1167 (9.9)	36 (10.5)	—	1193 (9.8)	10 (15.6)	—
18–30	2118 (17.4)	2040 (17.2)	78 (22.7)	—	2088 (17.2)	30 (46.9)	—
31–40	1574 (12.9)	1518 (12.8)	56 (16.3)	—	1564 (12.9)	10 (15.6)	—
41–50	2220 (18.2)	2148 (18.1)	72 (20.9)	—	2212 (18.3)	8 (12.5)	—
51–60	1868 (15.3)	1823 (15.4)	45 (13.1)	—	1866 (15.4)	2 (3.1)	—
61–80	2786 (22.9)	2730 (23.1)	56 (16.3)	—	2785 (23.0)	1 (1.6)	—
>80	364 (3.0)	364 (3.1)	0 (0.0)	—	362 (3.0)	2 (3.1)	—
Marital status (%)	—	—	—	**0.069**	—	—	**<0.001**
Divorced	508 (4.2)	489 (4.1)	19 (5.5)	—	507 (4.2)	1 (1.6)	—
Widowed	501 (4.1)	488 (4.1)	13 (3.8)	—	501 (4.1)	0 (0.0)	—
Single/unmarried	3074 (25.2)	2970 (25.1)	104 (30.2)	—	3034 (25.0)	40 (62.5)	—
Married	8099 (66.5)	7891 (66.7)	208 (60.5)	—	8076 (66.6)	23 (35.9)	—
Job status (%)	—	—	—	**0.016**	—	—	**<0.001**
Labor	1922 (15.8)	1888 (15.9)	34 (9.9)	—	1916 (15.8)	6 (9.4)	—
Public servant	608 (5.0)	584 (4.9)	24 (7.0)	—	604 (5.0)	4 (6.2)	—
Office	656 (5.4)	633 (5.3)	23 (6.7)	—	647 (5.3)	9 (14.1)	—
Other	2315 (19.0)	2251 (19.0)	64 (18.6)	—	2307 (19.0)	8 (12.5)	—
Retired	1976 (16.2)	1933 (16.3)	43 (12.5)	—	1974 (16.3)	2 (3.1)	—
Unemployed	843 (6.9)	818 (6.9)	25 (7.3)	—	837 (6.9)	6 (9.4)	—
Specialized	592 (4.9)	573 (4.8)	19 (5.5)	—	588 (4.9)	4 (6.2)	—
Student	2122 (17.4)	2049 (17.3)	73 (21.2)	—	2099 (17.3)	23 (35.9)	—
Management	627 (5.1)	603 (5.1)	24 (7.0)	—	627 (5.2)	0 (0.0)	—
Freelancer	521 (4.3)	506 (4.3)	15 (4.4)	—	519 (4.3)	2 (3.1)	—
Recurrent depression (%)	—	—	—	**<0.001**	—	—	**1**
False	11,365 (93.3)	11,064 (93.5)	301 (87.5)	—	11,305 (93.3)	60 (93.8)	—
True	817 (6.7)	774 (6.5)	43 (12.5)	—	813 (6.7)	4 (6.2)	—
Psychiatric comorbidity (%)	—	—	—	**<0.001**	—	—	**0.035**
False	7486 (61.5)	7221 (61.0)	265 (77.0)	—	7438 (61.4)	48 (75.0)	—
True	4696 (38.5)	4617 (39.0)	79 (23.0)	—	4680 (38.6)	16 (25.0)	—
Endocrine comorbidity (%)	—	—	—	**<0.001**	—	—	**0.044**
False	9485 (77.9)	9178 (77.5)	307 (89.2)	—	9428 (77.8)	57 (89.1)	—
True	2697 (22.1)	2660 (22.5)	37 (10.8)	—	2690 (22.2)	7 (10.9)	—
Nervous comorbidity (%)	—	—	—	**<0.001**	—	—	**0.511**
False	11,034 (90.6)	10,700 (90.4)	334 (97.1)	—	10,974 (90.6)	60 (93.8)	—
True	1148 (9.4)	1138 (9.6)	10 (2.9)	—	1144 (9.4)	4 (6.2)	—
Digestive comorbidity (%)	—	—	—	**<0.001**	—	—	**0.027**
False	9806 (80.5)	9490 (80.2)	316 (91.9)	—	9747 (80.4)	59 (92.2)	—
True	2376 (19.5)	2348 (19.8)	28 (8.1)	—	2371 (19.6)	5 (7.8)	—
Circulatory comorbidity (%)	—	—	—	**<0.001**	—	—	**<0.001**
False	9263 (76.0)	8961 (75.7)	302 (87.8)	—	9201 (75.9)	62 (96.9)	—
True	2919 (24.0)	2877 (24.3)	42 (12.2)	—	2917 (24.1)	2 (3.1)	—
Respiratory comorbidity (%)	—	—	—	**0.001**	—	—	**0.197**
False	10,682 (87.7)	10,360 (87.5)	322 (93.6)	—	10,622 (87.7)	60 (93.8)	—
True	1500 (12.3)	1478 (12.5)	22 (6.4)	—	1496 (12.3)	4 (6.2)	—
Cancer comorbidity (%)	—	—	—	**0.009**	—	—	**0.129**
False	11,591 (95.1)	11,253 (95.1)	338 (98.3)	—	11,527 (95.1)	64 (100.0)	—
True	591 (4.9)	585 (4.9)	6 (1.7)	—	591 (4.9)	0 (0.0)	—
Family history of mental illness (%)	—	—	—	**0.24**	—	—	**<0.001**
False	11,522 (94.6)	11,202 (94.6)	320 (93.0)	—	11,470 (94.7)	52 (81.2)	—
True	660 (5.4)	636 (5.4)	24 (7.0)	—	648 (5.3)	12 (18.8)	—
History of smoking (%)	—	—	—	**0.108**	—	—	**0.201**
False	8772 (72.0)	8538 (72.1)	234 (68.0)	—	8731 (72.0)	41 (64.1)	—
True	3410 (28.0)	3300 (27.9)	110 (32.0)	—	3387 (28.0)	23 (35.9)	—
History of drinking (%)	—	—	—	**0.796**	—	—	**0.885**
None	9482 (77.8)	9218 (77.9)	264 (76.7)	—	9432 (77.8)	50 (78.1)	—
Often	941 (7.7)	915 (7.7)	26 (7.6)	—	937 (7.7)	4 (6.2)	—
Sometime	1759 (14.4)	1705 (14.4)	54 (15.7)	—	1749 (14.4)	10 (15.6)	—
History of medication use (%)		—	—	**0.02**	—	—	**0.113**
None	9861 (80.9)	9600 (81.1)	261 (75.9)	—	9806 (80.9)	55 (85.9)	—
Often	1741 (14.3)	1674 (14.1)	67 (19.5)	—	1737 (14.3)	4 (6.2)	—
Sometime	580 (4.8)	564 (4.8)	16 (4.7)	—	575 (4.7)	5 (7.8)	—

*Note*: Bold values indicate the *p*-value results among a large number of numbers and does not carry any other significance.

Abbreviations: BD, bipolar disorder; SCZ, schizophrenia.

## Data Availability

The data used to support the findings of this manuscript are restricted by the West China Hospital to protect patient privacy and avoid legal and ethical risks. Data are available from West China Hospital for researchers who meet the criteria for access to confidential data.

## Supporting Information

Supporting Information Supporting information associated with this article can be found, in the online version, which included details about unstructured data processing and laboratory data processing, quality of raw data, results of statistical hypothesis tests (features comparison between BD converters, and nonconverters, SCZ converters and nonconverters), rates of conversions from UD to BD/SCZ stratified by some predictors (sex, single-episode or recurrent depression, psychotic symptoms), performance of prediction models using the LASSO/RF method filtered features, important predictors of conversion to BD and SCZ at population-level and at individual-level. The supporting figures and tables can also be found in this file, such as Figure S1. Cumulative conversion incidences and yearly crude incidence rate for the conversion from UD to BD and SCZ stratified by sex. Figure S2. Cumulative conversion incidences and yearly crude incidence rate for the conversion from UD to BD and SCZ stratified by history of family mental illness. Figure S3. Cumulative conversion incidences and yearly crude incidence rate for the conversion from UD to BD and SCZ stratified by single-episode or recurrent depression. Figure S4. Cumulative conversion incidences and yearly crude incidence rate for the conversion from UD to BD and SCZ stratified by psychotic symptoms. Figure S5. The performance of prediction models using the LASSO method filtered features for the conversion from UD to BD. The area under the receiver operating characteristic (AUC) ROC curve. The tables showed the internal validation performance (i.e., sensitivity, specificity, positive predictive value [PPV], and negative predictive value [NPV]) of prediction models. Figure S6. The performance of prediction models using the RF method filtered features for the conversion from UD to BD. The area under the receiver operating characteristic (AUC) ROC curve. The tables showed the internal validation performance (i.e., sensitivity, specificity, positive predictive value [PPV], and negative predictive value [NPV]) of prediction models. Figure S7. The performance of prediction models using the LASSO method filtered features for the conversion from UD to SCZ. The area under the receiver operating characteristic (AUC) ROC curve. The tables showed the internal validation performance (i.e., sensitivity, specificity, positive predictive value [PPV], and negative predictive value [NPV]) of prediction models. Figure S8. The performance of prediction models using the RF method filtered features for the conversion from UD to SCZ. The area under the receiver operating characteristic (AUC) ROC curve. The tables showed the internal validation performance (i.e., sensitivity, specificity, positive predictive value [PPV], and negative predictive value [NPV]) of prediction models. Figure S9. The comparison of the top 10 features to predict conversion and nonconversion from UD to BD within different temporal windows. The green bar represents the positive contribution features, and the red bar represents the negative contribution features. Figure S10. The comparison of the top 10 features to predict conversion and nonconversion from UD to SCZ within different temporal windows. The green bar represents the positive contribution features, and the red bar represents the negative contribution features. Table S1. Features recruited into the raw data of sub-cohort 1. Table S2. Features comparison between converters and nonconverters.
