# Supplementary material for: Predicting Conversion From Unipolar Depression to Bipolar Disorder and Schizophrenia: A 10-Year Retrospective Cohort Study on 12,182 Inpatients
Source: Depress Anxiety. 2025 Feb 20;2025:4048082. doi: 10.1155/da/4048082 (PMC11919011; doi:10.1155/da/4048082)
Supplement: Supporting Information — associated with this article can be found, in the online version, which included details about unstructured data processing and laboratory data processing, quality of raw data, results of statistical hypothesis tests (features comparison between BD converters, and nonconverters, SCZ converters and nonconverters), rates of conversions from UD to BD/SCZ stratified by some predictors (sex, single-episode or recurrent depression, psychotic symptoms), performance of prediction models using the LASSO/RF method filtered features, important predictors of conversion to BD and SCZ at population-level and at individual-level. The supporting figures and tables can also be found in this file, such as Figure S1. Cumulative conversion incidences and yearly crude incidence rate for the conversion from UD to BD and SCZ stratified by sex. Figure S2. Cumulative conversion incidences and yearly crude incidence rate for the conversion from UD to BD and SCZ stratified by history of family mental illness. Figure S3. Cumulative conversion incidences and yearly crude incidence rate for the conversion from UD to BD and SCZ stratified by single-episode or recurrent depression. Figure S4. Cumulative conversion incidences and yearly crude incidence rate for the conversion from UD to BD and SCZ stratified by psychotic symptoms. Figure S5. The performance of prediction models using the LASSO method filtered features for the conversion from UD to BD. The area under the receiver operating characteristic (AUC) ROC curve. The tables showed the internal validation performance (i.e., sensitivity, specificity, positive predictive value [PPV], and negative predictive value [NPV]) of prediction models. Figure S6. The performance of prediction models using the RF method filtered features for the conversion from UD to BD. The area under the receiver operating characteristic (AUC) ROC curve. The tables showed the internal validation performance (i.e., sensitivity, specificity, positive [file 4048082.f1.docx]

**Supplementary Information**

**Supplementary Information SI1:**

- 1. **Unstructured data processing and laboratory data processing**

In this study, unstructured data like patients’ chief complaint was processed. The text data of chief complaint includes Chinese words, numbers, and punctuation marks. Chinese words record patients’ main symptoms, current mood states, the specific symptom’s duration, frequency, and severity. Sometimes, the specific symptom’s duration and frequency can be recorded as numbers. The text data was split into words by jieba package of R (Version 3.6.1 for Windows). The process is as follows:

Since we aimed at extracting key words of patients’ symptoms as predictors, we first removed all numbers and punctuation marks by using the R function “gsub”. Second, we downloaded a Chinese stop-word dictionary from CSDN (<https://blog.csdn.net/shijiebei2009/article/details/39696571>) and some medical dictionaries from the Sogou Thesaurus (<https://pinyin.sogou.com/dict/>). The medical dictionaries included medical professional dictionary, doctor psychology dictionary, medicine name dictionary, psychology dictionary, and drug name dictionary. Moreover, we defined some stop words (such as time unit, more, less, etc.) and symptom words (such as mood-down, mood-up, bad sleep, loss of interest, etc.) to supplement the downloaded dictionaries. By using the jieba package of R, the text data of each patient’s chief complaint was segmented into words, and the frequency of each word was counted. The term frequency scores were used to identify the most informative words in patients’ chief complaints and these words were referred to as features of patients’ main symptoms and current mood states (mood-down, mood-unstable, bad sleep, worry, dizziness, pain, physical discomfort, fatigue, suicide ideation, less activity, paranoid, provoke, recurrence and worsen of symptoms, etc.). The value of each of the symptom feature depends on whether the chief complaint of this patient includes the above key words or not, particularly, 1 for including the specific word and 0 for not including the specific word.

For the sixth item of data, each patient might have more than once laboratory tests during the index hospitalization. We extracted the first recorded laboratory test results during the inpatient stay of all subjects as the laboratory diagnostic criteria and analyzed all laboratory data including routine blood and biochemical examination. For each laboratory test type, only the most common result types (listed by result name like platelet count, red blood cell count, absolute of lymphocyte, etc.) were used in the models. For each laboratory indicator, the numerical value or the categorical level (in the normal range or higher or lower) were included as predictors. No laboratory data after the first recorded date were included in models.

- 1. **Quality of raw data and data preprocessing**

Our database consists of a representative set of items to record all information generated during a patient’s hospitalization. According to prior studies, the diagnosis conversion from UD to BD or SCZ was driven by the interaction of a variety of genetical, biological, clinical, psychological, behavior, pharmacological, social, and environmental factors. We examined the effects of the following predictor variables on risk of conversion from UD to BD/SCZ. Specifically, 1) Basic information, 2) Hospitalization information, 3) Past history, 4) Physical examination at admission, 5) Chie complaint, 6) Laboratory test, 7) Treatment-related information.

Table S1 Features recruited into the raw data of sub-cohort 1.

| **Variable name** | **Variable interpretation** | **Data type** |
| --- | --- | --- |
| PADMNO | Patient admission number (unique for each admission record of each patient) | factor |
| sex | Sex | factor |
| age | Age group | factor |
| marital.status | Marital status | factor |
| job.status | Job status | factor |
| IF.F | Whether the patient is admitted to a psychiatric ward | factor |
| if.surgery | Whether the patient has a surgery during the hospitalization | factor |
| psychiatric.comorbidity.number | The number of psychiatric comobidity | factor |
| psychiatric.comorbidity | Whether the patient has another psychiatric disease | factor |
| endocrine.comorbidity.number | The number of endocrine system comobidity | factor |
| endocrine.comorbidity | Whether the patient has an endocrine system disease | factor |
| nerve.comorbidity.number | The number of nervous system comobidity | factor |
| nerve.comorbidity | Whether the patient has a nervous system disease | factor |
| digestive.comorbidity.number | The number of digestive system comobidity | factor |
| digestive.comorbidity | Whether the patient has a digestive system disease | factor |
| circulatory.comorbidity.number | The number of circulatory system comobidity | factor |
| circulatory.comorbidity | Whether the patient has a circulatory system disease | factor |
| respiratory.comorbidity.number | The number of respiratory system comobidity | factor |
| respiratory.comorbidity | Whether the patient has a respiratory system disease | factor |
| cancer.comorbidity.number | The number of cancer | factor |
| cancer.comorbidity | Whether the patient has a cancer | factor |
| recurrent.depression | Whether the patient is diagnosed as a recurrent depression | factor |
| discharge.type | The type of patient discharge | factor |
| trauma.his | History of trauma | factor |
| surgery.his | History of surgery | factor |
| allergy.his | History of allergy | factor |
| transblood.his | History of transblood | factor |
| smoking.his | History of smoking | factor |
| drinking.his | History of drinking | factor |
| drug.his | History of medication use | factor |
| family.his | Family history of mental illness | factor |
| revision.times | The number of revision times of the primary diagnosis | factor |
| chief_complaint | Chief complaint | factor |
| mooddown | Whether the patient has a symptom of mood-down | factor |
| moodunstable | Whether the patient has a symptom of mood-unstable | factor |
| sleepbad | Whether the patient has a symptom of bad sleep | factor |
| worry | Whether the patient has a symptom of worry | factor |
| physical.discomfortable | Whether the patient has a physical discomfortable symptom | factor |
| fatigue | Whether the patient has a symptom of fatigue | factor |
| decline | Whether the patient has a symptom of decline | factor |
| pain | Whether the patient has a symptom of pain | factor |
| dizzy | Whether the patient has a symptom of dizzy | factor |
| paranoid | Whether the patient has psychotic symptoms | factor |
| suicide | Whether the patient has a symptom of suicide ideation/attemption/behavior | factor |
| provoke | Whether the patient has a symptom of provoke | factor |
| relapse | Whether the patient has a recurrence of above symptoms | factor |
| worsen | Whether the patient has a worsen of above symptoms | factor |
| Los | Length of stay | numeric |
| temperature | Physical examination: to record a patient's body temperature | numeric |
| pulse | Physical examination: to record a patient's pulse | numeric |
| DP | Physical examination: to record a patient's diastolic blood pressure (DBP) | numeric |
| SP | Physical examination: to record a patient's systolic blood pressure (SBP) | numeric |
| breathing | Physical examination: to record a patient's breath | numeric |
| RBC_CV_value | Routine blood examination: Red blood cell volume distribution width CV | numeric |
| RBC_CV_level | Routine blood examination: Level of red blood cell volume distribution width CV | factor |
| RBC_SD_value | Routine blood examination: Red blood cell volume distribution width SD | numeric |
| RBC_SD_level | Routine blood examination: Level of red blood cell volume distribution width SD | factor |
| WBCC_value | Routine blood examination: White blood cell count (Leukocyte count) | numeric |
| WBCC_level | Routine blood examination: Level of white blood cell count (Leukocyte count) | factor |
| POM_value | Routine blood examination: Percentage value of monocyte | numeric |
| POM_level | Routine blood examination: Level of percentage value of monocyte | factor |
| AOM_value | Routine blood examination: Absolute value of monocyte | numeric |
| AOM_level | Routine blood examination: Level of absolute value of monocyte | factor |
| RBCC_value | Routine blood examination: Red blood cell count (Erythrocyte Count) | numeric |
| RBCC_level | Routine blood examination: Level of red blood cell count (Erythrocyte Count) | factor |
| hematocrit_value | Routine blood examination: Hematocrit value | numeric |
| hematocrit_level | Routine blood examination: Level of hematocrit value | factor |
| POL_value | Routine blood examination: Percentage value of lymphocyte | numeric |
| POL_level | Routine blood examination: Level of percentage value of lymphocyte | factor |
| AOL_value | Routine blood examination: Absolute value of lymphocyte | numeric |
| AOL_level | Routine blood examination: Level of absolute value of lymphocyte | factor |
| ARBC_HGB_value | Routine blood examination: Average value of red blood cell (erythrocyte) HGB | numeric |
| ARBC_ HGB_level | Routine blood examination: Level of average value of red blood cell (erythrocyte) HGB | factor |
| ARBC_HGB_con_value | Routine blood examination: Average value of red blood cell (erythrocyte) HGB concentration | numeric |
| ARBC_HGB_con_level | Routine blood examination: Level of average value of red blood cell (erythrocyte) HGB concentration | factor |
| ARBCV_value | Routine blood examination: Average value of red blood cell volume | numeric |
| ARBCV_level | Routine blood examination: Level of average value of red blood cell volume | factor |
| POB_value | Routine blood examination: Percentage value of basophil | numeric |
| POB_level | Routine blood examination: Level of percentage value of basophil | factor |
| POE_value | Routine blood examination: Percentage value of eosinophil | numeric |
| POE_level | Routine blood examination: Level of percentage value of eosinophil | factor |
| hemoglobin_value | Routine blood examination: Hemoglobin value | numeric |
| hemoglobin_level | Routine blood examination: Level of hemoglobin value | factor |
| PC_value | Routine blood examination: Platelet count | numeric |
| PC_level | Routine blood examination: Level of platelet count | factor |
| PON_value | Routine blood examination: Percentage value of neutrophilic segmented granulocyte | numeric |
| PON_level | Routine blood examination: Level of percentage value of neutrophilic segmented granulocyte | factor |
| AON_value | Routine blood examination: Absolute value of neutrophilic segmented granulocyte | numeric |
| AON_level | Routine blood examination: Level of absolute value of neutrophilic segmented granulocyte | factor |
| ALA_value | Routine biochemical examination: Alanine aminotransferase | numeric |
| ALA_level | Routine biochemical examination: Level of alanine aminotransferase | factor |
| ASA_value | Routine biochemical examination: Aspartate aminotransferase | numeric |
| ASA_level | Routine biochemical examination: Level of aspartate aminotransferase | factor |
| CK_value | Routine biochemical examination: Creatine kinase | numeric |
| CK_level | Routine biochemical examination: Level of creatine kinase | factor |
| LD_value | Routine biochemical examination: Lactate dehydrogenase | numeric |
| LD_level | Routine biochemical examination: Level of lactate dehydrogenase | factor |
| urea_value | Routine biochemical examination: Urea | numeric |
| urea_level | Routine biochemical examination: Level of urea | factor |
| TB_value | Routine biochemical examination: Total bilirubin | numeric |
| TB_level | Routine biochemical examination: Level of total bilirubin | factor |
| DB_value | Routine biochemical examination: Direct bilirubin | numeric |
| DB_level | Routine biochemical examination: Level of direct bilirubin | factor |
| IDB_value | Routine biochemical examination: Indirect bilirubin | numeric |
| IDB_level | Routine biochemical examination: Level of indirect bilirubin | factor |
| TP_value | Routine biochemical examination: Total protein | numeric |
| TP_level | Routine biochemical examination: Level of total protein | factor |
| albumin_value | Routine biochemical examination: Albumin | numeric |
| albumin_level | Routine biochemical examination: Level of albumin | factor |
| creatinine_value | Routine biochemical examination: Creatinine | numeric |
| creatinine_level | Routine biochemical examination: Level of creatinine | factor |
| glucose_value | Routine biochemical examination: Glucose | numeric |
| glucose_level | Routine biochemical examination: Level of glucose | factor |
| AP_value | Routine biochemical examination: Alkaline phosphatase | numeric |
| AP_level | Routine biochemical examination: Level of alkaline phosphatase | factor |
| GT_value | Routine biochemical examination: Glutamyl transpeptidase | numeric |
| GT_level | Routine biochemical examination: Level of glutamyl transpeptidase | factor |
| sodium_value | Routine biochemical examination: Sodium | numeric |
| sodium_level | Routine biochemical examination: Level of sodium | factor |
| potassium_value | Routine biochemical examination: Potassium | numeric |
| potassium_level | Routine biochemical examination: Level of potassium | factor |
| chlorine_value | Routine biochemical examination: Chlorine | numeric |
| chlorine_level | Routine biochemical examination: Level of chlorine | factor |
| globulin_value | Routine biochemical examination: Globulin | numeric |
| globulin_level | Routine biochemical examination: Level of globulin | factor |
| WBR_value | Routine biochemical examination: White ball ratio | numeric |
| WBR_level | Routine biochemical examination: Level of white ball ratio | factor |
| UA_value | Routine biochemical examination: Uric acid | numeric |
| UA_level | Routine biochemical examination: Level of uric acid | factor |
| HD_value | Routine biochemical examination: Hydroxybutyrate dehydrogenase | numeric |
| HD_level | Routine biochemical examination: Level of hydroxybutyrate dehydrogenase | factor |
| cholesterol_value | Routine biochemical examination: Cholesterol | numeric |
| cholesterol_level | Routine biochemical examination: Level of cholesterol | factor |
| HDL_value | Routine biochemical examination: High density lipoprotein | numeric |
| HDL_level | Routine biochemical examination: Level of high density lipoprotein | factor |
| LDL_value | Routine biochemical examination: Low density lipoprotein | numeric |
| LDL_level | Routine biochemical examination: Level of low density lipoprotein | factor |
| cystatin C_value | Routine biochemical examination: Serum cystatin C | numeric |
| cystatin C_level | Routine biochemical examination: Level of serum cystatin C | factor |
| calcium_value | Routine biochemical examination: Calcium | numeric |
| calcium_level | Routine biochemical examination: Level of calcium | factor |
| magnesium_value | Routine biochemical examination:Magnesium | numeric |
| magnesium_level | Routine biochemical examination: Level of magnesium | factor |
| SIP_value | Routine biochemical examination: Serum inorganic phosphorus | numeric |
| SIP_level | Routine biochemical examination: Level of serum inorganic phosphorus | factor |
| CO2CP_value | Routine biochemical examination: Carbon dioxide combining power | numeric |
| CO2CP_level | Routine biochemical examination: Level of carbon dioxide combining power | factor |
| AG_value | Routine biochemical examination: Aninon gap | numeric |
| AG_level | Routine biochemical examination: Level of aninon gap | factor |
| ADP_type | Type of antidepressants used for each patient | factor |
| AP_type | Type of antipsychotics used for each patient | factor |
| AA_type | Type of anxiolytics used for each patient | factor |
| MSB_type | Type of mood stabilizers used for each patient | factor |
| ASE_type | Type of anti-side effects drugs used for each patient | factor |
| HYP_type | Type of new hypnotics used for each patient | factor |
| OT_type | Type of β receptor blockers used for each patient | factor |
| T3_type | Type of hormonal drugs used for each patient | factor |
| CM_type | Type of Chinese patent medicines used for each patient | factor |
| PHY_type | Type of physiotherapies used for each patient | factor |
| PSY_type | Type of psychotherapies used for each patient | factor |
| SUM | Sum of medical orders prescribed for each patient | numeric |
| ADP_SUM | Sum of antidepressant orders prescribed for each patient | numeric |
| AMTL | Number of orders of amitriptyline hydrochloride tablets prescribed for each patient | numeric |
| ASXTPL | Number of orders of escitalopram oxalate tablets prescribed for each patient | numeric |
| BMQ | Number of orders of imipramine hydrochloride tablets prescribed for each patient | numeric |
| DLXT | Number of orders of duloxetine hydrochloride enteric-coated capsules prescribed for each patient | numeric |
| DSP | Number of orders of doxepin hydrochloride tablets prescribed for each patient | numeric |
| FFSM | Number of orders of fluvoxamine maleate tablets prescribed for each patient | numeric |
| FPSDMLQX | Number of orders of flupentixol and meritroxine tablets prescribed for each patient | numeric |
| FXT | Number of orders of fluoxetine hydrochloride dispersible tablets prescribed for each patient | numeric |
| LMPM | Number of orders of clomipramine hydrochloride tablets prescribed for each patient | numeric |
| MDP | Number of orders of mirtazapine tablets prescribed for each patient | numeric |
| MPTL | Number of orders of maprotiline hydrochloride tablets prescribed for each patient | numeric |
| PLXT | Number of orders of paroxetine hydrochloride tablets prescribed for each patient | numeric |
| SQL | Number of orders of sertraline hydrochloride tablets prescribed for each patient | numeric |
| XTPL | Number of orders of citalopram hydrobromide tablets prescribed for each patient | numeric |
| YSQZT | Number of orders of trazodone hydrochloride tablets prescribed for each patient | numeric |
| YSWLFX | Number of orders of venlafaxine hydrochloride sustained-release capsules prescribed for each patient | numeric |
| AP_SUM | Sum of antipsychotic orders prescribed for each patient | numeric |
| ADanP | Number of orders of olanzapine tablets prescribed for each patient | numeric |
| AHBL | Number of orders of amisulpride tablets prescribed for each patient | numeric |
| ALPZ | Number of orders of aripiprazole tablets prescribed for each patient | numeric |
| FMSZLP | Number of orders of quetiapine fumrate tablets prescribed for each patient | numeric |
| FPDC | Number of orders of haloperidol tablets prescribed for each patient | numeric |
| FPDCZSY | Number of orders of haloperidol for injection prescribed for each patient | numeric |
| LBL | Number of orders of tiapride hydrochloride tablets prescribed for each patient | numeric |
| LBQ | Number of orders of chlorpromazine hydrochloride tablets prescribed for each patient | numeric |
| LDP | Number of orders of clozapine tablets prescribed for each patient | numeric |
| LPT | Number of orders of risperidone tablets prescribed for each patient | numeric |
| PLPT | Number of orders of paliperidone sustained-release tablets prescribed for each patient | numeric |
| SBL | Number of orders of sulpiride prescribed for each patient | numeric |
| SBLZSY | Number of orders of sulpiride for injection prescribed for each patient | numeric |
| YSQLXT | Number of orders of ziprasidone hydrochloride capsules prescribed for each patient | numeric |
| AA_SUM | Sum of anxiolytic orders prescribed for each patient | numeric |
| APZL | Number of orders of alprazolam tablets prescribed for each patient | numeric |
| ASZL | Number of orders of estazolam tablets prescribed for each patient | numeric |
| DLHT | Number of orders of buspirone hydrochloride tablets prescribed for each patient | numeric |
| DXP | Number of orders of diazepam tablets prescribed for each patient | numeric |
| LLXZ | Number of orders of lorazepam tablets prescribed for each patient | numeric |
| LXXP | Number of orders of clonazepam tablets prescribed for each patient | numeric |
| MDZL | Number of orders of midazolam maleate tablets prescribed for each patient | numeric |
| MDZLZSY | Number of orders of midazolam for injection prescribed for each patient | numeric |
| TDLTJL | Number of orders of tandospirone citrate capsules prescribed for each patient | numeric |
| XXP | Number of orders of nitrazepam tablets prescribed for each patient | numeric |
| YZPKL | Number of orders of ezopiclone tablets prescribed for each patient | numeric |
| MSB_SUM | Sum of mood stabilizer orders prescribed for each patient | numeric |
| BWSN | Number of orders of sodium valproate sustained-release tablets prescribed for each patient | numeric |
| JBPD | Number of orders of gabapentin capsules prescribed for each patient | numeric |
| KMXP | Number of orders of carbamazepine tablets prescribed for each patient | numeric |
| LMSZ | Number of orders of lamotrigine tablets prescribed for each patient | numeric |
| TBZ | Number of orders of topiramate capsules prescribed for each patient | numeric |
| TSZ | Number of orders of lithium carbonate tablets prescribed for each patient | numeric |
| ASE_SUM | Sum of anti-side effects drugs orders prescribed for each patient | numeric |
| ASPL | Number of orders of aspirin enteric-coated tablets prescribed for each patient | numeric |
| ATFTTG | Number of orders of atorvastatin clacium tablets prescribed for each patient | numeric |
| BHS | Number of orders of benhexol hydrochloride tablets prescribed for each patient | numeric |
| BSKD | Number of orders of bisacodyl enteric-coated tablets prescribed for each patient | numeric |
| JYEC | Number of orders of polyethylene glycol prescribed for each patient | numeric |
| KSL | Number of orders of glycerine enema prescribed for each patient | numeric |
| MR | Number of orders of maren maru prescribed for each patient | numeric |
| MSBL | Number of orders of mosapride citrate tablets prescribed for each patient | numeric |
| HYP_SUM | Sum of new hypnotic orders prescribed for each patient | numeric |
| ZPKL | Number of orders of zopiclone tablets prescribed for each patient | numeric |
| ZZT | Number of orders of zopiclone tartrate tablets prescribed for each patient | numeric |
| OT_SUM | Sum of β receptor blocker orders prescribed for each patient | numeric |
| BSLE | Number of orders of bisoprolol fumarate tablets prescribed for each patient | numeric |
| HPSMTLE | Number of orders of metoprolol succinate sustained-release tablets prescribed for each patient | numeric |
| JSSMTLE | Number of orders of metoprolol tartrate tablets prescribed for each patient | numeric |
| PNLE | Number of orders of propranolol hydrochloride tablets prescribed for each patient | numeric |
| ZJZXSN | Number of orders of levothyroxine sodium tablets prescribed for each patient | numeric |
| GWS | Number of orders of oryzanol tablets prescribed for each patient | numeric |
| PHY_SUM | Sum of physiotherapy orders prescribed for each patient | numeric |
| MPBT | Number of orders of multi-parameter biofeedback therapy prescribed for each patient | numeric |
| ME | Number of orders of modified electroconvulsive therapy prescribed for each patient | numeric |
| TMS | Number of orders of transcranial magnetic stimulation therapy prescribed for each patient | numeric |
| TRMS | Number of orders of transcranial repetitive magnetic stimulation therapy prescribed for each patient | numeric |
| EEG | Number of orders of electroencephalographic (EEG) biofeedback therapy prescribed for each patient | numeric |
| BT | Number of orders of biofeedback therapy prescribed for each patient | numeric |
| PSY | Number of orders of psychotherapy prescribed for each patient | numeric |

**Supplementary Information SI2:**

Table S2 Features comparison between converters and non-converters.

| predictor | level | Overall | BD non-converters | BD converters | p value | SCZ non-converters | SCZ converters | p value |
| --- | --- | --- | --- | --- | --- | --- | --- | --- |
| n |  | 12182 | 11838 | 344 |  | 12118 | 64 |  |
| sex (%) | F | 8032 (65.9) | 7781 (65.7) | 251 (73.0) | 0.006 | 7996 (66.0) | 36 (56.2) | 0.132 |
|  | M | 4150 (34.1) | 4057 (34.3) | 93 (27.0) |  | 4122 (34.0) | 28 (43.8) |  |
| age.group (%) | <13 | 49 (0.4) | 48 (0.4) | 1 (0.3) | <0.001 | 48 (0.4) | 1 (1.6) | <0.001 |
|  | >80 | 364 (3.0) | 364 (3.1) | 0 (0.0) |  | 362 (3.0) | 2 (3.1) |  |
|  | 13-17 | 1203 (9.9) | 1167 (9.9) | 36 (10.5) |  | 1193 (9.8) | 10 (15.6) |  |
|  | 18-30 | 2118 (17.4) | 2040 (17.2) | 78 (22.7) |  | 2088 (17.2) | 30 (46.9) |  |
|  | 31-40 | 1574 (12.9) | 1518 (12.8) | 56 (16.3) |  | 1564 (12.9) | 10 (15.6) |  |
|  | 41-50 | 2220 (18.2) | 2148 (18.1) | 72 (20.9) |  | 2212 (18.3) | 8 (12.5) |  |
|  | 51-60 | 1868 (15.3) | 1823 (15.4) | 45 (13.1) |  | 1866 (15.4) | 2 (3.1) |  |
|  | 61-80 | 2786 (22.9) | 2730 (23.1) | 56 (16.3) |  | 2785 (23.0) | 1 (1.6) |  |
| job.status (%) | freelance | 521 (4.3) | 506 (4.3) | 15 (4.4) | 0.016 | 519 (4.3) | 2 (3.1) | <0.001 |
|  | labor | 1922 (15.8) | 1888 (15.9) | 34 (9.9) |  | 1916 (15.8) | 6 (9.4) |  |
|  | management | 627 (5.1) | 603 (5.1) | 24 (7.0) |  | 627 (5.2) | 0 (0.0) |  |
|  | office | 656 (5.4) | 633 (5.3) | 23 (6.7) |  | 647 (5.3) | 9 (14.1) |  |
|  | other | 2315 (19.0) | 2251 (19.0) | 64 (18.6) |  | 2307 (19.0) | 8 (12.5) |  |
|  | public servant | 608 (5.0) | 584 (4.9) | 24 (7.0) |  | 604 (5.0) | 4 (6.2) |  |
|  | retired | 1976 (16.2) | 1933 (16.3) | 43 (12.5) |  | 1974 (16.3) | 2 (3.1) |  |
|  | specialised | 592 (4.9) | 573 (4.8) | 19 (5.5) |  | 588 (4.9) | 4 (6.2) |  |
|  | student | 2122 (17.4) | 2049 (17.3) | 73 (21.2) |  | 2099 (17.3) | 23 (35.9) |  |
|  | unemployed | 843 (6.9) | 818 (6.9) | 25 (7.3) |  | 837 (6.9) | 6 (9.4) |  |
| marital.status (%) | divorced | 508 (4.2) | 489 (4.1) | 19 (5.5) | 0.069 | 507 (4.2) | 1 (1.6) | <0.001 |
|  | married | 8099 (66.5) | 7891 (66.7) | 208 (60.5) |  | 8076 (66.6) | 23 (35.9) |  |
|  | single | 3074 (25.2) | 2970 (25.1) | 104 (30.2) |  | 3034 (25.0) | 40 (62.5) |  |
|  | widowed | 501 (4.1) | 488 (4.1) | 13 (3.8) |  | 501 (4.1) | 0 (0.0) |  |
| IF.F (%) | FALSE | 2917 (23.9) | 2896 (24.5) | 21 (6.1) | <0.001 | 2913 (24.0) | 4 (6.2) | 0.001 |
|  | TRUE | 9265 (76.1) | 8942 (75.5) | 323 (93.9) |  | 9205 (76.0) | 60 (93.8) |  |
| if.surgery (%) | FALSE | 10806 (88.7) | 10476 (88.5) | 330 (95.9) | <0.001 | 10747 (88.7) | 59 (92.2) | 0.494 |
|  | TRUE | 1376 (11.3) | 1362 (11.5) | 14 (4.1) |  | 1371 (11.3) | 5 (7.8) |  |
| psychiatric.comorbidity.number (%) | 0 | 7486 (61.5) | 7221 (61.0) | 265 (77.0) | <0.001 | 7438 (61.4) | 48 (75.0) | 0.278 |
|  | 1 | 4152 (34.1) | 4079 (34.5) | 73 (21.2) |  | 4138 (34.1) | 14 (21.9) |  |
|  | 2 | 510 (4.2) | 504 (4.3) | 6 (1.7) |  | 508 (4.2) | 2 (3.1) |  |
|  | 3 | 33 (0.3) | 33 (0.3) | 0 (0.0) |  | 33 (0.3) | 0 (0.0) |  |
|  | 4 | 1 (0.0) | 1 (0.0) | 0 (0.0) |  | 1 (0.0) | 0 (0.0) |  |
| psychiatric.comorbidity (%) | FALSE | 7486 (61.5) | 7221 (61.0) | 265 (77.0) | <0.001 | 7438 (61.4) | 48 (75.0) | 0.035 |
|  | TRUE | 4696 (38.5) | 4617 (39.0) | 79 (23.0) |  | 4680 (38.6) | 16 (25.0) |  |
| endocrine.comorbidity.number (%) | 0 | 9485 (77.9) | 9178 (77.5) | 307 (89.2) | <0.001 | 9428 (77.8) | 57 (89.1) | 0.653 |
|  | 1 | 1976 (16.2) | 1945 (16.4) | 31 (9.0) |  | 1970 (16.3) | 6 (9.4) |  |
|  | 2 | 523 (4.3) | 519 (4.4) | 4 (1.2) |  | 522 (4.3) | 1 (1.6) |  |
|  | 3 | 146 (1.2) | 144 (1.2) | 2 (0.6) |  | 146 (1.2) | 0 (0.0) |  |
|  | 4 | 32 (0.3) | 32 (0.3) | 0 (0.0) |  | 32 (0.3) | 0 (0.0) |  |
|  | 5 | 15 (0.1) | 15 (0.1) | 0 (0.0) |  | 15 (0.1) | 0 (0.0) |  |
|  | 6 | 4 (0.0) | 4 (0.0) | 0 (0.0) |  | 4 (0.0) | 0 (0.0) |  |
|  | 7 | 1 (0.0) | 1 (0.0) | 0 (0.0) |  | 1 (0.0) | 0 (0.0) |  |
| endocrine.comorbidity (%) | FALSE | 9485 (77.9) | 9178 (77.5) | 307 (89.2) | <0.001 | 9428 (77.8) | 57 (89.1) | 0.044 |
|  | TRUE | 2697 (22.1) | 2660 (22.5) | 37 (10.8) |  | 2690 (22.2) | 7 (10.9) |  |
| nerve.comorbidity.number (%) | 0 | 11034 (90.6) | 10700 (90.4) | 334 (97.1) | 0.001 | 10974 (90.6) | 60 (93.8) | 0.861 |
|  | 1 | 954 (7.8) | 945 (8.0) | 9 (2.6) |  | 950 (7.8) | 4 (6.2) |  |
|  | 2 | 155 (1.3) | 155 (1.3) | 0 (0.0) |  | 155 (1.3) | 0 (0.0) |  |
|  | 3 | 37 (0.3) | 36 (0.3) | 1 (0.3) |  | 37 (0.3) | 0 (0.0) |  |
|  | 4 | 2 (0.0) | 2 (0.0) | 0 (0.0) |  | 2 (0.0) | 0 (0.0) |  |
| nerve.comorbidity (%) | FALSE | 11034 (90.6) | 10700 (90.4) | 334 (97.1) | <0.001 | 10974 (90.6) | 60 (93.8) | 0.511 |
|  | TRUE | 1148 (9.4) | 1138 (9.6) | 10 (2.9) |  | 1144 (9.4) | 4 (6.2) |  |
| digestive.comorbidity.number (%) | 0 | 9806 (80.5) | 9490 (80.2) | 316 (91.9) | <0.001 | 9747 (80.4) | 59 (92.2) | 0.489 |
|  | 1 | 1675 (13.7) | 1652 (14.0) | 23 (6.7) |  | 1670 (13.8) | 5 (7.8) |  |
|  | 2 | 510 (4.2) | 505 (4.3) | 5 (1.5) |  | 510 (4.2) | 0 (0.0) |  |
|  | 3 | 130 (1.1) | 130 (1.1) | 0 (0.0) |  | 130 (1.1) | 0 (0.0) |  |
|  | 4 | 36 (0.3) | 36 (0.3) | 0 (0.0) |  | 36 (0.3) | 0 (0.0) |  |
|  | 5 | 17 (0.1) | 17 (0.1) | 0 (0.0) |  | 17 (0.1) | 0 (0.0) |  |
|  | 6 | 7 (0.1) | 7 (0.1) | 0 (0.0) |  | 7 (0.1) | 0 (0.0) |  |
|  | 7 | 1 (0.0) | 1 (0.0) | 0 (0.0) |  | 1 (0.0) | 0 (0.0) |  |
| digestive.comorbidity (%) | FALSE | 9806 (80.5) | 9490 (80.2) | 316 (91.9) | <0.001 | 9747 (80.4) | 59 (92.2) | 0.027 |
|  | TRUE | 2376 (19.5) | 2348 (19.8) | 28 (8.1) |  | 2371 (19.6) | 5 (7.8) |  |
| circulatory.comorbidity.number (%) | 0 | 9263 (76.0) | 8961 (75.7) | 302 (87.8) | <0.001 | 9201 (75.9) | 62 (96.9) | <0.001 |
|  | 1 | 1737 (14.3) | 1701 (14.4) | 36 (10.5) |  | 1737 (14.3) | 0 (0.0) |  |
|  | 2 | 635 (5.2) | 631 (5.3) | 4 (1.2) |  | 635 (5.2) | 0 (0.0) |  |
|  | 3 | 271 (2.2) | 269 (2.3) | 2 (0.6) |  | 271 (2.2) | 0 (0.0) |  |
|  | 4 | 149 (1.2) | 149 (1.3) | 0 (0.0) |  | 148 (1.2) | 1 (1.6) |  |
|  | 5 | 66 (0.5) | 66 (0.6) | 0 (0.0) |  | 66 (0.5) | 0 (0.0) |  |
|  | 6 | 35 (0.3) | 35 (0.3) | 0 (0.0) |  | 35 (0.3) | 0 (0.0) |  |
|  | 7 | 17 (0.1) | 17 (0.1) | 0 (0.0) |  | 17 (0.1) | 0 (0.0) |  |
|  | 8 | 6 (0.0) | 6 (0.1) | 0 (0.0) |  | 5 (0.0) | 1 (1.6) |  |
|  | 9 | 3 (0.0) | 3 (0.0) | 0 (0.0) |  | 3 (0.0) | 0 (0.0) |  |
| circulatory.comorbidity (%) | FALSE | 9263 (76.0) | 8961 (75.7) | 302 (87.8) | <0.001 | 9201 (75.9) | 62 (96.9) | <0.001 |
|  | TRUE | 2919 (24.0) | 2877 (24.3) | 42 (12.2) |  | 2917 (24.1) | 2 (3.1) |  |
| respiratory.comorbidity.number (%) | 0 | 10682 (87.7) | 10360 (87.5) | 322 (93.6) | 0.019 | 10622 (87.7) | 60 (93.8) | 0.602 |
|  | 1 | 1147 (9.4) | 1129 (9.5) | 18 (5.2) |  | 1145 (9.4) | 2 (3.1) |  |
|  | 2 | 271 (2.2) | 268 (2.3) | 3 (0.9) |  | 269 (2.2) | 2 (3.1) |  |
|  | 3 | 64 (0.5) | 64 (0.5) | 0 (0.0) |  | 64 (0.5) | 0 (0.0) |  |
|  | 4 | 16 (0.1) | 15 (0.1) | 1 (0.3) |  | 16 (0.1) | 0 (0.0) |  |
|  | 5 | 2 (0.0) | 2 (0.0) | 0 (0.0) |  | 2 (0.0) | 0 (0.0) |  |
| respiratory.comorbidity (%) | FALSE | 10682 (87.7) | 10360 (87.5) | 322 (93.6) | 0.001 | 10622 (87.7) | 60 (93.8) | 0.197 |
|  | TRUE | 1500 (12.3) | 1478 (12.5) | 22 (6.4) |  | 1496 (12.3) | 4 (6.2) |  |
| cancer.comorbidity.number (%) | 0 | 11591 (95.1) | 11253 (95.1) | 338 (98.3) | 0.586 | 11527 (95.1) | 64 (100.0) | 0.974 |
|  | 1 | 370 (3.0) | 365 (3.1) | 5 (1.5) |  | 370 (3.1) | 0 (0.0) |  |
|  | 11 | 1 (0.0) | 1 (0.0) | 0 (0.0) |  | 1 (0.0) | 0 (0.0) |  |
|  | 2 | 75 (0.6) | 75 (0.6) | 0 (0.0) |  | 75 (0.6) | 0 (0.0) |  |
|  | 3 | 43 (0.4) | 43 (0.4) | 0 (0.0) |  | 43 (0.4) | 0 (0.0) |  |
|  | 4 | 46 (0.4) | 45 (0.4) | 1 (0.3) |  | 46 (0.4) | 0 (0.0) |  |
|  | 5 | 28 (0.2) | 28 (0.2) | 0 (0.0) |  | 28 (0.2) | 0 (0.0) |  |
|  | 6 | 13 (0.1) | 13 (0.1) | 0 (0.0) |  | 13 (0.1) | 0 (0.0) |  |
|  | 7 | 6 (0.0) | 6 (0.1) | 0 (0.0) |  | 6 (0.0) | 0 (0.0) |  |
|  | 8 | 7 (0.1) | 7 (0.1) | 0 (0.0) |  | 7 (0.1) | 0 (0.0) |  |
|  | 9 | 2 (0.0) | 2 (0.0) | 0 (0.0) |  | 2 (0.0) | 0 (0.0) |  |
| cancer.comorbidity (%) | FALSE | 11591 (95.1) | 11253 (95.1) | 338 (98.3) | 0.009 | 11527 (95.1) | 64 (100.0) | 0.129 |
|  | TRUE | 591 (4.9) | 585 (4.9) | 6 (1.7) |  | 591 (4.9) | 0 (0.0) |  |
| recurrent.depression (%) | FALSE | 11365 (93.3) | 11064 (93.5) | 301 (87.5) | <0.001 | 11305 (93.3) | 60 (93.8) | 1 |
|  | TRUE | 817 (6.7) | 774 (6.5) | 43 (12.5) |  | 813 (6.7) | 4 (6.2) |  |
| F41 (%) | FALSE | 11034 (90.6) | 10721 (90.6) | 313 (91.0) | 0.864 | 10974 (90.6) | 60 (93.8) | 0.511 |
|  | TRUE | 1148 (9.4) | 1117 (9.4) | 31 (9.0) |  | 1144 (9.4) | 4 (6.2) |  |
| dischage.type (%) | doctor.discharge | 10355 (85.0) | 10081 (85.2) | 274 (79.7) | 0.001 | 10301 (85.0) | 54 (84.4) | 0.013 |
|  | doctor.transfer | 71 (0.6) | 69 (0.6) | 2 (0.6) |  | 71 (0.6) | 0 (0.0) |  |
|  | other | 1394 (11.4) | 1332 (11.3) | 62 (18.0) |  | 1390 (11.5) | 4 (6.2) |  |
|  | self.discharge | 362 (3.0) | 356 (3.0) | 6 (1.7) |  | 356 (2.9) | 6 (9.4) |  |
| trauma.his (%) | FALSE | 11730 (96.3) | 11389 (96.2) | 341 (99.1) | 0.007 | 11668 (96.3) | 62 (96.9) | 1 |
|  | TRUE | 452 (3.7) | 449 (3.8) | 3 (0.9) |  | 450 (3.7) | 2 (3.1) |  |
| surgery.his (%) | FALSE | 7513 (61.7) | 7305 (61.7) | 208 (60.5) | 0.681 | 7461 (61.6) | 52 (81.2) | 0.002 |
|  | TRUE | 4669 (38.3) | 4533 (38.3) | 136 (39.5) |  | 4657 (38.4) | 12 (18.8) |  |
| allergy.his (%) | FALSE | 10425 (85.6) | 10132 (85.6) | 293 (85.2) | 0.89 | 10367 (85.6) | 58 (90.6) | 0.33 |
|  | TRUE | 1757 (14.4) | 1706 (14.4) | 51 (14.8) |  | 1751 (14.4) | 6 (9.4) |  |
| transblood.his (%) | FALSE | 11721 (96.2) | 11386 (96.2) | 335 (97.4) | 0.313 | 11657 (96.2) | 64 (100.0) | 0.207 |
|  | TRUE | 461 (3.8) | 452 (3.8) | 9 (2.6) |  | 461 (3.8) | 0 (0.0) |  |
| smoking.his (%) | FALSE | 8772 (72.0) | 8538 (72.1) | 234 (68.0) | 0.108 | 8731 (72.0) | 41 (64.1) | 0.201 |
|  | TRUE | 3410 (28.0) | 3300 (27.9) | 110 (32.0) |  | 3387 (28.0) | 23 (35.9) |  |
| drinking.his (%) | none | 9482 (77.8) | 9218 (77.9) | 264 (76.7) | 0.796 | 9432 (77.8) | 50 (78.1) | 0.885 |
|  | often | 941 (7.7) | 915 (7.7) | 26 (7.6) |  | 937 (7.7) | 4 (6.2) |  |
|  | sometime | 1759 (14.4) | 1705 (14.4) | 54 (15.7) |  | 1749 (14.4) | 10 (15.6) |  |
| drug.his (%) | none | 9861 (80.9) | 9600 (81.1) | 261 (75.9) | 0.02 | 9806 (80.9) | 55 (85.9) | 0.113 |
|  | often | 1741 (14.3) | 1674 (14.1) | 67 (19.5) |  | 1737 (14.3) | 4 (6.2) |  |
|  | sometime | 580 (4.8) | 564 (4.8) | 16 (4.7) |  | 575 (4.7) | 5 (7.8) |  |
| famliy.illness (%) | FALSE | 11522 (94.6) | 11202 (94.6) | 320 (93.0) | 0.24 | 11470 (94.7) | 52 (81.2) | <0.001 |
|  | TRUE | 660 (5.4) | 636 (5.4) | 24 (7.0) |  | 648 (5.3) | 12 (18.8) |  |
| revision.times (%) | 0 | 10716 (88.0) | 10428 (88.1) | 288 (83.7) | 0.17 | 10661 (88.0) | 55 (85.9) | 0.62 |
|  | 1 | 1196 (9.8) | 1151 (9.7) | 45 (13.1) |  | 1187 (9.8) | 9 (14.1) |  |
|  | 2 | 212 (1.7) | 203 (1.7) | 9 (2.6) |  | 212 (1.7) | 0 (0.0) |  |
|  | 3 | 37 (0.3) | 36 (0.3) | 1 (0.3) |  | 37 (0.3) | 0 (0.0) |  |
|  | 4 | 21 (0.2) | 20 (0.2) | 1 (0.3) |  | 21 (0.2) | 0 (0.0) |  |
| mooddown (%) | FALSE | 5424 (44.5) | 5347 (45.2) | 77 (22.4) | <0.001 | 5402 (44.6) | 22 (34.4) | 0.131 |
|  | TRUE | 6758 (55.5) | 6491 (54.8) | 267 (77.6) |  | 6716 (55.4) | 42 (65.6) |  |
| worsen (%) | FALSE | 7505 (61.6) | 7303 (61.7) | 202 (58.7) | 0.289 | 7462 (61.6) | 43 (67.2) | 0.429 |
|  | TRUE | 4677 (38.4) | 4535 (38.3) | 142 (41.3) |  | 4656 (38.4) | 21 (32.8) |  |
| sleepbad (%) | FALSE | 8153 (66.9) | 7945 (67.1) | 208 (60.5) | 0.012 | 8110 (66.9) | 43 (67.2) | 1 |
|  | TRUE | 4029 (33.1) | 3893 (32.9) | 136 (39.5) |  | 4008 (33.1) | 21 (32.8) |  |
| relapse (%) | FALSE | 8265 (67.8) | 8080 (68.3) | 185 (53.8) | <0.001 | 8219 (67.8) | 46 (71.9) | 0.577 |
|  | TRUE | 3917 (32.2) | 3758 (31.7) | 159 (46.2) |  | 3899 (32.2) | 18 (28.1) |  |
| worry (%) | FALSE | 9803 (80.5) | 9541 (80.6) | 262 (76.2) | 0.048 | 9753 (80.5) | 50 (78.1) | 0.752 |
|  | TRUE | 2379 (19.5) | 2297 (19.4) | 82 (23.8) |  | 2365 (19.5) | 14 (21.9) |  |
| fatigue (%) | FALSE | 11282 (92.6) | 10948 (92.5) | 334 (97.1) | 0.002 | 11219 (92.6) | 63 (98.4) | 0.122 |
|  | TRUE | 900 (7.4) | 890 (7.5) | 10 (2.9) |  | 899 (7.4) | 1 (1.6) |  |
| decline (%) | FALSE | 10794 (88.6) | 10502 (88.7) | 292 (84.9) | 0.034 | 10735 (88.6) | 59 (92.2) | 0.48 |
|  | TRUE | 1388 (11.4) | 1336 (11.3) | 52 (15.1) |  | 1383 (11.4) | 5 (7.8) |  |
| pain (%) | FALSE | 10556 (86.7) | 10238 (86.5) | 318 (92.4) | 0.002 | 10494 (86.6) | 62 (96.9) | 0.026 |
|  | TRUE | 1626 (13.3) | 1600 (13.5) | 26 (7.6) |  | 1624 (13.4) | 2 (3.1) |  |
| dizzy (%) | FALSE | 11305 (92.8) | 10974 (92.7) | 331 (96.2) | 0.017 | 11242 (92.8) | 63 (98.4) | 0.132 |
|  | TRUE | 877 (7.2) | 864 (7.3) | 13 (3.8) |  | 876 (7.2) | 1 (1.6) |  |
| physical.discomfort (%) | FALSE | 9949 (81.7) | 9657 (81.6) | 292 (84.9) | 0.136 | 9891 (81.6) | 58 (90.6) | 0.09 |
|  | TRUE | 2233 (18.3) | 2181 (18.4) | 52 (15.1) |  | 2227 (18.4) | 6 (9.4) |  |
| suicide (%) | FALSE | 11581 (95.1) | 11260 (95.1) | 321 (93.3) | 0.163 | 11521 (95.1) | 60 (93.8) | 0.843 |
|  | TRUE | 601 (4.9) | 578 (4.9) | 23 (6.7) |  | 597 (4.9) | 4 (6.2) |  |
| paranoid (%) | FALSE | 11600 (95.2) | 11271 (95.2) | 329 (95.6) | 0.811 | 11555 (95.4) | 45 (70.3) | <0.001 |
|  | TRUE | 582 (4.8) | 567 (4.8) | 15 (4.4) |  | 563 (4.6) | 19 (29.7) |  |
| moodunstable (%) | FALSE | 11876 (97.5) | 11541 (97.5) | 335 (97.4) | 1 | 11815 (97.5) | 61 (95.3) | 0.475 |
|  | TRUE | 306 (2.5) | 297 (2.5) | 9 (2.6) |  | 303 (2.5) | 3 (4.7) |  |
| provoke (%) | FALSE | 11304 (92.8) | 10999 (92.9) | 305 (88.7) | 0.004 | 11243 (92.8) | 61 (95.3) | 0.59 |
|  | TRUE | 878 (7.2) | 839 (7.1) | 39 (11.3) |  | 875 (7.2) | 3 (4.7) |  |
| ALA_level (%) | h | 1090 (8.9) | 1067 (9.0) | 23 (6.7) | 0.235 | 1085 (9.0) | 5 (7.8) | 0.895 |
|  | l | 22 (0.2) | 22 (0.2) | 0 (0.0) |  | 22 (0.2) | 0 (0.0) |  |
|  | z | 11070 (90.9) | 10749 (90.8) | 321 (93.3) |  | 11011 (90.9) | 59 (92.2) |  |
| ASA_level (%) | h | 942 (7.7) | 924 (7.8) | 18 (5.2) | 0.209 | 939 (7.7) | 3 (4.7) | 0.656 |
|  | l | 1 (0.0) | 1 (0.0) | 0 (0.0) |  | 1 (0.0) | 0 (0.0) |  |
|  | z | 11239 (92.3) | 10913 (92.2) | 326 (94.8) |  | 11178 (92.2) | 61 (95.3) |  |
| CK_level (%) | h | 747 (6.1) | 726 (6.1) | 21 (6.1) | 0.325 | 738 (6.1) | 9 (14.1) | 0.022 |
|  | l | 138 (1.1) | 137 (1.2) | 1 (0.3) |  | 138 (1.1) | 0 (0.0) |  |
|  | z | 11297 (92.7) | 10975 (92.7) | 322 (93.6) |  | 11242 (92.8) | 55 (85.9) |  |
| LD_level (%) | h | 913 (7.5) | 902 (7.6) | 11 (3.2) | 0.007 | 912 (7.5) | 1 (1.6) | 0.139 |
|  | l | 785 (6.4) | 759 (6.4) | 26 (7.6) |  | 779 (6.4) | 6 (9.4) |  |
|  | z | 10484 (86.1) | 10177 (86.0) | 307 (89.2) |  | 10427 (86.0) | 57 (89.1) |  |
| urea_level (%) | h | 502 (4.1) | 498 (4.2) | 4 (1.2) | 0.02 | 501 (4.1) | 1 (1.6) | 0.586 |
|  | l | 1135 (9.3) | 1101 (9.3) | 34 (9.9) |  | 1129 (9.3) | 6 (9.4) |  |
|  | z | 10545 (86.6) | 10239 (86.5) | 306 (89.0) |  | 10488 (86.5) | 57 (89.1) |  |
| TB_level (%) | h | 203 (1.7) | 201 (1.7) | 2 (0.6) | 0.075 | 203 (1.7) | 0 (0.0) | 0.431 |
|  | l | 855 (7.0) | 823 (7.0) | 32 (9.3) |  | 852 (7.0) | 3 (4.7) |  |
|  | z | 11124 (91.3) | 10814 (91.3) | 310 (90.1) |  | 11063 (91.3) | 61 (95.3) |  |
| DB_level (%) | h | 344 (2.8) | 340 (2.9) | 4 (1.2) | 0.139 | 343 (2.8) | 1 (1.6) | 0.801 |
|  | l | 13 (0.1) | 13 (0.1) | 0 (0.0) |  | 13 (0.1) | 0 (0.0) |  |
|  | z | 11825 (97.1) | 11485 (97.0) | 340 (98.8) |  | 11762 (97.1) | 63 (98.4) |  |
| IDB_level (%) | h | 138 (1.1) | 137 (1.2) | 1 (0.3) | 0.215 | 138 (1.1) | 0 (0.0) | 0.79 |
|  | z | 12044 (98.9) | 11701 (98.8) | 343 (99.7) |  | 11980 (98.9) | 64 (100.0) |  |
| TP_level (%) | h | 31 (0.3) | 31 (0.3) | 0 (0.0) | 0.631 | 31 (0.3) | 0 (0.0) | 0.738 |
|  | l | 3494 (28.7) | 3394 (28.7) | 100 (29.1) |  | 3478 (28.7) | 16 (25.0) |  |
|  | z | 8657 (71.1) | 8413 (71.1) | 244 (70.9) |  | 8609 (71.0) | 48 (75.0) |  |
| albumin_level (%) | h | 1 (0.0) | 1 (0.0) | 0 (0.0) | 0.986 | 1 (0.0) | 0 (0.0) | 0.084 |
|  | l | 2972 (24.4) | 2888 (24.4) | 84 (24.4) |  | 2964 (24.5) | 8 (12.5) |  |
|  | z | 9209 (75.6) | 8949 (75.6) | 260 (75.6) |  | 9153 (75.5) | 56 (87.5) |  |
| creatinine_level (%) | h | 177 (1.5) | 177 (1.5) | 0 (0.0) | 0.013 | 177 (1.5) | 0 (0.0) | 0.245 |
|  | l | 334 (2.7) | 330 (2.8) | 4 (1.2) |  | 334 (2.8) | 0 (0.0) |  |
|  | z | 11671 (95.8) | 11331 (95.7) | 340 (98.8) |  | 11607 (95.8) | 64 (100.0) |  |
| glucose_level (%) | h | 1676 (13.8) | 1648 (13.9) | 28 (8.1) | 0.002 | 1669 (13.8) | 7 (10.9) | 0.651 |
|  | l | 343 (2.8) | 338 (2.9) | 5 (1.5) |  | 342 (2.8) | 1 (1.6) |  |
|  | z | 10163 (83.4) | 9852 (83.2) | 311 (90.4) |  | 10107 (83.4) | 56 (87.5) |  |
| AP_level (%) | h | 387 (3.2) | 382 (3.2) | 5 (1.5) | 0.011 | 387 (3.2) | 0 (0.0) | 0.346 |
|  | l | 1430 (11.7) | 1375 (11.6) | 55 (16.0) |  | 1422 (11.7) | 8 (12.5) |  |
|  | z | 10365 (85.1) | 10081 (85.2) | 284 (82.6) |  | 10309 (85.1) | 56 (87.5) |  |
| GT_level (%) | h | 1270 (10.4) | 1243 (10.5) | 27 (7.8) | 0.132 | 1268 (10.5) | 2 (3.1) | 0.154 |
|  | l | 11 (0.1) | 10 (0.1) | 1 (0.3) |  | 11 (0.1) | 0 (0.0) |  |
|  | z | 10901 (89.5) | 10585 (89.4) | 316 (91.9) |  | 10839 (89.4) | 62 (96.9) |  |
| sodium_level (%) | h | 345 (2.8) | 331 (2.8) | 14 (4.1) | <0.001 | 344 (2.8) | 1 (1.6) | 0.185 |
|  | l | 528 (4.3) | 527 (4.5) | 1 (0.3) |  | 528 (4.4) | 0 (0.0) |  |
|  | z | 11309 (92.8) | 10980 (92.8) | 329 (95.6) |  | 11246 (92.8) | 63 (98.4) |  |
| potassium_level (%) | h | 25 (0.2) | 25 (0.2) | 0 (0.0) | 0.688 | 25 (0.2) | 0 (0.0) | 0.832 |
|  | l | 943 (7.7) | 917 (7.7) | 26 (7.6) |  | 937 (7.7) | 6 (9.4) |  |
|  | z | 11214 (92.1) | 10896 (92.0) | 318 (92.4) |  | 11156 (92.1) | 58 (90.6) |  |
| chlorine_level (%) | h | 511 (4.2) | 483 (4.1) | 28 (8.1) | <0.001 | 508 (4.2) | 3 (4.7) | 0.447 |
|  | l | 876 (7.2) | 869 (7.3) | 7 (2.0) |  | 874 (7.2) | 2 (3.1) |  |
|  | z | 10795 (88.6) | 10486 (88.6) | 309 (89.8) |  | 10736 (88.6) | 59 (92.2) |  |
| globulin_level (%) | h | 97 (0.8) | 95 (0.8) | 2 (0.6) | 0.628 | 97 (0.8) | 0 (0.0) | 0.77 |
|  | l | 784 (6.4) | 758 (6.4) | 26 (7.6) |  | 780 (6.4) | 4 (6.2) |  |
|  | z | 11301 (92.8) | 10985 (92.8) | 316 (91.9) |  | 11241 (92.8) | 60 (93.8) |  |
| WBR_level (%) | h | 229 (1.9) | 221 (1.9) | 8 (2.3) | 0.408 | 228 (1.9) | 1 (1.6) | 0.792 |
|  | l | 593 (4.9) | 581 (4.9) | 12 (3.5) |  | 591 (4.9) | 2 (3.1) |  |
|  | z | 11360 (93.3) | 11036 (93.2) | 324 (94.2) |  | 11299 (93.2) | 61 (95.3) |  |
| UA_level (%) | h | 787 (6.5) | 769 (6.5) | 18 (5.2) | 0.043 | 781 (6.4) | 6 (9.4) | 0.466 |
|  | l | 915 (7.5) | 900 (7.6) | 15 (4.4) |  | 912 (7.5) | 3 (4.7) |  |
|  | z | 10480 (86.0) | 10169 (85.9) | 311 (90.4) |  | 10425 (86.0) | 55 (85.9) |  |
| HD_level (%) | h | 781 (6.4) | 772 (6.5) | 9 (2.6) | 0.014 | 779 (6.4) | 2 (3.1) | 0.509 |
|  | l | 34 (0.3) | 33 (0.3) | 1 (0.3) |  | 34 (0.3) | 0 (0.0) |  |
|  | z | 11367 (93.3) | 11033 (93.2) | 334 (97.1) |  | 11305 (93.3) | 62 (96.9) |  |
| cholesterol_level (%) | h | 1086 (8.9) | 1052 (8.9) | 34 (9.9) | 0.163 | 1085 (9.0) | 1 (1.6) | 0.106 |
|  | l | 478 (3.9) | 471 (4.0) | 7 (2.0) |  | 476 (3.9) | 2 (3.1) |  |
|  | z | 10618 (87.2) | 10315 (87.1) | 303 (88.1) |  | 10557 (87.1) | 61 (95.3) |  |
| HDL_level (%) | h | 1 (0.0) | 1 (0.0) | 0 (0.0) | 0.29 | 1 (0.0) | 0 (0.0) | 0.279 |
|  | l | 1065 (8.7) | 1043 (8.8) | 22 (6.4) |  | 1063 (8.8) | 2 (3.1) |  |
|  | z | 11116 (91.2) | 10794 (91.2) | 322 (93.6) |  | 11054 (91.2) | 62 (96.9) |  |
| LDL_level (%) | h | 454 (3.7) | 440 (3.7) | 14 (4.1) | 0.93 | 454 (3.7) | 0 (0.0) | 0.287 |
|  | l | 1 (0.0) | 1 (0.0) | 0 (0.0) |  | 1 (0.0) | 0 (0.0) |  |
|  | z | 11727 (96.3) | 11397 (96.3) | 330 (95.9) |  | 11663 (96.2) | 64 (100.0) |  |
| cystatinC_level (%) | h | 1472 (12.1) | 1448 (12.2) | 24 (7.0) | 0.011 | 1467 (12.1) | 5 (7.8) | 0.557 |
|  | l | 11 (0.1) | 11 (0.1) | 0 (0.0) |  | 11 (0.1) | 0 (0.0) |  |
|  | z | 10699 (87.8) | 10379 (87.7) | 320 (93.0) |  | 10640 (87.8) | 59 (92.2) |  |
| calcium_level (%) | h | 18 (0.1) | 18 (0.2) | 0 (0.0) | 0.757 | 18 (0.1) | 0 (0.0) | 0.277 |
|  | l | 1308 (10.7) | 1270 (10.7) | 38 (11.0) |  | 1305 (10.8) | 3 (4.7) |  |
|  | z | 10856 (89.1) | 10550 (89.1) | 306 (89.0) |  | 10795 (89.1) | 61 (95.3) |  |
| magnesium_level (%) | h | 456 (3.7) | 444 (3.8) | 12 (3.5) | 0.817 | 454 (3.7) | 2 (3.1) | 0.613 |
|  | l | 168 (1.4) | 162 (1.4) | 6 (1.7) |  | 168 (1.4) | 0 (0.0) |  |
|  | z | 11558 (94.9) | 11232 (94.9) | 326 (94.8) |  | 11496 (94.9) | 62 (96.9) |  |
| SIP_level (%) | h | 758 (6.2) | 730 (6.2) | 28 (8.1) | 0.301 | 755 (6.2) | 3 (4.7) | 0.875 |
|  | l | 1090 (8.9) | 1062 (9.0) | 28 (8.1) |  | 1084 (8.9) | 6 (9.4) |  |
|  | z | 10334 (84.8) | 10046 (84.9) | 288 (83.7) |  | 10279 (84.8) | 55 (85.9) |  |
| CO2CP_level (%) | h | 1393 (11.4) | 1365 (11.5) | 28 (8.1) | 0.125 | 1384 (11.4) | 9 (14.1) | 0.473 |
|  | l | 192 (1.6) | 185 (1.6) | 7 (2.0) |  | 190 (1.6) | 2 (3.1) |  |
|  | z | 10597 (87.0) | 10288 (86.9) | 309 (89.8) |  | 10544 (87.0) | 53 (82.8) |  |
| AG_level (%) | h | 2347 (19.3) | 2302 (19.4) | 45 (13.1) | 0.008 | 2338 (19.3) | 9 (14.1) | 0.227 |
|  | l | 923 (7.6) | 890 (7.5) | 33 (9.6) |  | 915 (7.6) | 8 (12.5) |  |
|  | z | 8912 (73.2) | 8646 (73.0) | 266 (77.3) |  | 8865 (73.2) | 47 (73.4) |  |
| RBC_CV_level (%) | h | 2002 (16.4) | 1951 (16.5) | 51 (14.8) | 0.668 | 2001 (16.5) | 1 (1.6) | 0.005 |
|  | l | 50 (0.4) | 49 (0.4) | 1 (0.3) |  | 50 (0.4) | 0 (0.0) |  |
|  | z | 10130 (83.2) | 9838 (83.1) | 292 (84.9) |  | 10067 (83.1) | 63 (98.4) |  |
| RBC_SD_level (%) | h | 418 (3.4) | 413 (3.5) | 5 (1.5) | 0.046 | 418 (3.4) | 0 (0.0) | 0.192 |
|  | l | 176 (1.4) | 174 (1.5) | 2 (0.6) |  | 176 (1.5) | 0 (0.0) |  |
|  | z | 11588 (95.1) | 11251 (95.0) | 337 (98.0) |  | 11524 (95.1) | 64 (100.0) |  |
| WBCC_level (%) | h | 599 (4.9) | 587 (5.0) | 12 (3.5) | 0.234 | 596 (4.9) | 3 (4.7) | 0.32 |
|  | l | 731 (6.0) | 705 (6.0) | 26 (7.6) |  | 730 (6.0) | 1 (1.6) |  |
|  | z | 10852 (89.1) | 10546 (89.1) | 306 (89.0) |  | 10792 (89.1) | 60 (93.8) |  |
| POM_level (%) | h | 746 (6.1) | 728 (6.1) | 18 (5.2) | 0.777 | 745 (6.1) | 1 (1.6) | 0.201 |
|  | l | 149 (1.2) | 145 (1.2) | 4 (1.2) |  | 149 (1.2) | 0 (0.0) |  |
|  | z | 11287 (92.7) | 10965 (92.6) | 322 (93.6) |  | 11224 (92.6) | 63 (98.4) |  |
| AOM_level (%) | h | 907 (7.4) | 888 (7.5) | 19 (5.5) | 0.329 | 899 (7.4) | 8 (12.5) | 0.138 |
|  | l | 60 (0.5) | 59 (0.5) | 1 (0.3) |  | 59 (0.5) | 1 (1.6) |  |
|  | z | 11215 (92.1) | 10891 (92.0) | 324 (94.2) |  | 11160 (92.1) | 55 (85.9) |  |
| RBCC_level (%) | h | 417 (3.4) | 409 (3.5) | 8 (2.3) | 0.286 | 417 (3.4) | 0 (0.0) | 0.016 |
|  | l | 1827 (15.0) | 1782 (15.1) | 45 (13.1) |  | 1824 (15.1) | 3 (4.7) |  |
|  | z | 9938 (81.6) | 9647 (81.5) | 291 (84.6) |  | 9877 (81.5) | 61 (95.3) |  |
| hematocrit_level (%) | h | 380 (3.1) | 374 (3.2) | 6 (1.7) | 0.031 | 378 (3.1) | 2 (3.1) | 0.022 |
|  | l | 1899 (15.6) | 1859 (15.7) | 40 (11.6) |  | 1897 (15.7) | 2 (3.1) |  |
|  | z | 9903 (81.3) | 9605 (81.1) | 298 (86.6) |  | 9843 (81.2) | 60 (93.8) |  |
| POL_level (%) | h | 544 (4.5) | 525 (4.4) | 19 (5.5) | 0.045 | 538 (4.4) | 6 (9.4) | 0.162 |
|  | l | 1782 (14.6) | 1747 (14.8) | 35 (10.2) |  | 1773 (14.6) | 9 (14.1) |  |
|  | z | 9856 (80.9) | 9566 (80.8) | 290 (84.3) |  | 9807 (80.9) | 49 (76.6) |  |
| AOL_level (%) | h | 269 (2.2) | 258 (2.2) | 11 (3.2) | 0.244 | 266 (2.2) | 3 (4.7) | 0.382 |
|  | l | 1526 (12.5) | 1490 (12.6) | 36 (10.5) |  | 1519 (12.5) | 7 (10.9) |  |
|  | z | 10387 (85.3) | 10090 (85.2) | 297 (86.3) |  | 10333 (85.3) | 54 (84.4) |  |
| ARBC_HGB_level (%) | h | 381 (3.1) | 366 (3.1) | 15 (4.4) | 0.379 | 380 (3.1) | 1 (1.6) | 0.287 |
|  | l | 932 (7.7) | 908 (7.7) | 24 (7.0) |  | 930 (7.7) | 2 (3.1) |  |
|  | z | 10869 (89.2) | 10564 (89.2) | 305 (88.7) |  | 10808 (89.2) | 61 (95.3) |  |
| ARBC_HGB_con_level (%) | h | 132 (1.1) | 131 (1.1) | 1 (0.3) | 0.194 | 132 (1.1) | 0 (0.0) | 0.194 |
|  | l | 1557 (12.8) | 1506 (12.7) | 51 (14.8) |  | 1553 (12.8) | 4 (6.2) |  |
|  | z | 10493 (86.1) | 10201 (86.2) | 292 (84.9) |  | 10433 (86.1) | 60 (93.8) |  |
| ARBCV_level (%) | h | 496 (4.1) | 482 (4.1) | 14 (4.1) | 0.995 | 494 (4.1) | 2 (3.1) | 0.33 |
|  | l | 687 (5.6) | 668 (5.6) | 19 (5.5) |  | 686 (5.7) | 1 (1.6) |  |
|  | z | 10999 (90.3) | 10688 (90.3) | 311 (90.4) |  | 10938 (90.3) | 61 (95.3) |  |
| POB_level (%) | h | 603 (4.9) | 586 (5.0) | 17 (4.9) | 1 | 600 (5.0) | 3 (4.7) | 1 |
|  | z | 11579 (95.1) | 11252 (95.0) | 327 (95.1) |  | 11518 (95.0) | 61 (95.3) |  |
| POE_level (%) | h | 351 (2.9) | 338 (2.9) | 13 (3.8) | 0.047 | 350 (2.9) | 1 (1.6) | 0.649 |
|  | l | 701 (5.8) | 691 (5.8) | 10 (2.9) |  | 696 (5.7) | 5 (7.8) |  |
|  | z | 11130 (91.4) | 10809 (91.3) | 321 (93.3) |  | 11072 (91.4) | 58 (90.6) |  |
| hemoglobin_level (%) | h | 297 (2.4) | 285 (2.4) | 12 (3.5) | 0.002 | 297 (2.5) | 0 (0.0) | 0.031 |
|  | l | 2007 (16.5) | 1973 (16.7) | 34 (9.9) |  | 2003 (16.5) | 4 (6.2) |  |
|  | z | 9878 (81.1) | 9580 (80.9) | 298 (86.6) |  | 9818 (81.0) | 60 (93.8) |  |
| PC_level (%) | h | 809 (6.6) | 794 (6.7) | 15 (4.4) | 0.024 | 805 (6.6) | 4 (6.2) | 0.753 |
|  | l | 689 (5.7) | 678 (5.7) | 11 (3.2) |  | 684 (5.6) | 5 (7.8) |  |
|  | z | 10684 (87.7) | 10366 (87.6) | 318 (92.4) |  | 10629 (87.7) | 55 (85.9) |  |
| PON_level (%) | h | 1284 (10.5) | 1256 (10.6) | 28 (8.1) | 0.273 | 1280 (10.6) | 4 (6.2) | 0.525 |
|  | l | 600 (4.9) | 580 (4.9) | 20 (5.8) |  | 597 (4.9) | 3 (4.7) |  |
|  | z | 10298 (84.5) | 10002 (84.5) | 296 (86.0) |  | 10241 (84.5) | 57 (89.1) |  |
| AON_level (%) | h | 780 (6.4) | 764 (6.5) | 16 (4.7) | 0.237 | 777 (6.4) | 3 (4.7) | 0.787 |
|  | l | 787 (6.5) | 769 (6.5) | 18 (5.2) |  | 782 (6.5) | 5 (7.8) |  |
|  | z | 10615 (87.1) | 10305 (87.1) | 310 (90.1) |  | 10559 (87.1) | 56 (87.5) |  |
| ADP_type (%) | 0 | 1552 (12.7) | 1530 (12.9) | 22 (6.4) | 0.002 | 1547 (12.8) | 5 (7.8) | 0.803 |
|  | 1 | 8734 (71.7) | 8484 (71.7) | 250 (72.7) |  | 8687 (71.7) | 47 (73.4) |  |
|  | 2 | 1674 (13.7) | 1609 (13.6) | 65 (18.9) |  | 1664 (13.7) | 10 (15.6) |  |
|  | 3 | 204 (1.7) | 197 (1.7) | 7 (2.0) |  | 202 (1.7) | 2 (3.1) |  |
|  | 4 | 17 (0.1) | 17 (0.1) | 0 (0.0) |  | 17 (0.1) | 0 (0.0) |  |
|  | 6 | 1 (0.0) | 1 (0.0) | 0 (0.0) |  | 1 (0.0) | 0 (0.0) |  |
| AP_type (%) | 0 | 5004 (41.1) | 4926 (41.6) | 78 (22.7) | <0.001 | 4994 (41.2) | 10 (15.6) | <0.001 |
|  | 1 | 5080 (41.7) | 4887 (41.3) | 193 (56.1) |  | 5048 (41.7) | 32 (50.0) |  |
|  | 2 | 1631 (13.4) | 1576 (13.3) | 55 (16.0) |  | 1615 (13.3) | 16 (25.0) |  |
|  | 3 | 369 (3.0) | 356 (3.0) | 13 (3.8) |  | 366 (3.0) | 3 (4.7) |  |
|  | 4 | 81 (0.7) | 78 (0.7) | 3 (0.9) |  | 79 (0.7) | 2 (3.1) |  |
|  | 5 | 11 (0.1) | 10 (0.1) | 1 (0.3) |  | 11 (0.1) | 0 (0.0) |  |
|  | 6 | 4 (0.0) | 3 (0.0) | 1 (0.3) |  | 4 (0.0) | 0 (0.0) |  |
|  | 7 | 2 (0.0) | 2 (0.0) | 0 (0.0) |  | 1 (0.0) | 1 (1.6) |  |
| AA_type (%) | 0 | 2041 (16.8) | 2019 (17.1) | 22 (6.4) | <0.001 | 2024 (16.7) | 17 (26.6) | 0.668 |
|  | 1 | 5239 (43.0) | 5113 (43.2) | 126 (36.6) |  | 5214 (43.0) | 25 (39.1) |  |
|  | 2 | 3239 (26.6) | 3113 (26.3) | 126 (36.6) |  | 3224 (26.6) | 15 (23.4) |  |
|  | 3 | 1262 (10.4) | 1209 (10.2) | 53 (15.4) |  | 1257 (10.4) | 5 (7.8) |  |
|  | 4 | 338 (2.8) | 324 (2.7) | 14 (4.1) |  | 336 (2.8) | 2 (3.1) |  |
|  | 5 | 53 (0.4) | 51 (0.4) | 2 (0.6) |  | 53 (0.4) | 0 (0.0) |  |
|  | 6 | 9 (0.1) | 8 (0.1) | 1 (0.3) |  | 9 (0.1) | 0 (0.0) |  |
|  | 7 | 1 (0.0) | 1 (0.0) | 0 (0.0) |  | 1 (0.0) | 0 (0.0) |  |
| MSB_type (%) | 0 | 10978 (90.1) | 10714 (90.5) | 264 (76.7) | <0.001 | 10920 (90.1) | 58 (90.6) | 0.982 |
|  | 1 | 1086 (8.9) | 1016 (8.6) | 70 (20.3) |  | 1081 (8.9) | 5 (7.8) |  |
|  | 2 | 112 (0.9) | 103 (0.9) | 9 (2.6) |  | 111 (0.9) | 1 (1.6) |  |
|  | 3 | 5 (0.0) | 4 (0.0) | 1 (0.3) |  | 5 (0.0) | 0 (0.0) |  |
|  | 4 | 1 (0.0) | 1 (0.0) | 0 (0.0) |  | 1 (0.0) | 0 (0.0) |  |
| ASE_type (%) | 0 | 7761 (63.7) | 7542 (63.7) | 219 (63.7) | 0.98 | 7729 (63.8) | 32 (50.0) | 0.028 |
|  | 1 | 2557 (21.0) | 2481 (21.0) | 76 (22.1) |  | 2535 (20.9) | 22 (34.4) |  |
|  | 2 | 1260 (10.3) | 1224 (10.3) | 36 (10.5) |  | 1254 (10.3) | 6 (9.4) |  |
|  | 3 | 448 (3.7) | 438 (3.7) | 10 (2.9) |  | 447 (3.7) | 1 (1.6) |  |
|  | 4 | 136 (1.1) | 133 (1.1) | 3 (0.9) |  | 133 (1.1) | 3 (4.7) |  |
|  | 5 | 16 (0.1) | 16 (0.1) | 0 (0.0) |  | 16 (0.1) | 0 (0.0) |  |
|  | 6 | 3 (0.0) | 3 (0.0) | 0 (0.0) |  | 3 (0.0) | 0 (0.0) |  |
|  | 7 | 1 (0.0) | 1 (0.0) | 0 (0.0) |  | 1 (0.0) | 0 (0.0) |  |
| HYP_type (%) | 0 | 11501 (94.4) | 11182 (94.5) | 319 (92.7) | 0.21 | 11440 (94.4) | 61 (95.3) | 0.966 |
|  | 1 | 681 (5.6) | 656 (5.5) | 25 (7.3) |  | 678 (5.6) | 3 (4.7) |  |
| OT_type (%) | 0 | 10065 (82.6) | 9807 (82.8) | 258 (75.0) | 0.001 | 10012 (82.6) | 53 (82.8) | 0.806 |
|  | 1 | 2037 (16.7) | 1953 (16.5) | 84 (24.4) |  | 2026 (16.7) | 11 (17.2) |  |
|  | 2 | 80 (0.7) | 78 (0.7) | 2 (0.6) |  | 80 (0.7) | 0 (0.0) |  |
| T3_type (%) | 0 | 11786 (96.7) | 11451 (96.7) | 335 (97.4) | 0.604 | 11724 (96.7) | 62 (96.9) | 1 |
|  | 1 | 396 (3.3) | 387 (3.3) | 9 (2.6) |  | 394 (3.3) | 2 (3.1) |  |
| CM_type (%) | 0 | 10648 (87.4) | 10352 (87.4) | 296 (86.0) | 0.491 | 10586 (87.4) | 62 (96.9) | 0.036 |
|  | 1 | 1534 (12.6) | 1486 (12.6) | 48 (14.0) |  | 1532 (12.6) | 2 (3.1) |  |
| PHY_type (%) | 0 | 6787 (55.7) | 6633 (56.0) | 154 (44.8) | <0.001 | 6741 (55.6) | 46 (71.9) | 0.131 |
|  | 1 | 4472 (36.7) | 4320 (36.5) | 152 (44.2) |  | 4457 (36.8) | 15 (23.4) |  |
|  | 2 | 802 (6.6) | 772 (6.5) | 30 (8.7) |  | 799 (6.6) | 3 (4.7) |  |
|  | 3 | 115 (0.9) | 108 (0.9) | 7 (2.0) |  | 115 (0.9) | 0 (0.0) |  |
|  | 4 | 6 (0.0) | 5 (0.0) | 1 (0.3) |  | 6 (0.0) | 0 (0.0) |  |
| PSY_type (%) | 0 | 6022 (49.4) | 5904 (49.9) | 118 (34.3) | <0.001 | 5995 (49.5) | 27 (42.2) | 0.3 |
|  | 1 | 6160 (50.6) | 5934 (50.1) | 226 (65.7) |  | 6123 (50.5) | 37 (57.8) |  |
| age (median [IQR]) | | 46.00 [28.00, 61.00] | 46.00 [28.00, 61.00] | 41.00 [24.00, 53.00] | <0.001 | 46.00 [28.00, 61.00] | 26.00 [19.00, 37.25] | <0.001 |
| Los (median [IQR]) | | 14.00 [10.00, 21.00] | 14.00 [10.00, 20.00] | 18.00 [13.00, 23.25] | <0.001 | 14.00 [10.00, 20.00] | 16.00 [13.00, 23.25] | 0.033 |
| temperature (median [IQR]) | | 36.50 [36.40, 36.70] | 36.50 [36.40, 36.70] | 36.50 [36.40, 36.70] | 0.029 | 36.50 [36.40, 36.70] | 36.50 [36.40, 36.70] | 0.802 |
| pluse (median [IQR]) | | 80.00 [73.00, 89.00] | 80.00 [73.00, 89.00] | 80.00 [75.00, 91.00] | 0.052 | 80.00 [73.00, 89.00] | 85.50 [79.75, 96.50] | <0.001 |
| breathing (median [IQR]) | | 20.00 [20.00, 20.00] | 20.00 [20.00, 20.00] | 20.00 [20.00, 20.00] | 0.004 | 20.00 [20.00, 20.00] | 20.00 [20.00, 20.00] | 0.375 |
| SP (median [IQR]) | | 121.00 [111.00, 132.00] | 121.00 [111.00, 132.00] | 119.00 [109.00, 129.00] | 0.011 | 121.00 [111.00, 132.00] | 116.00 [109.50, 124.00] | 0.003 |
| DP (median [IQR]) | | 77.00 [70.00, 84.00] | 77.00 [70.00, 84.00] | 76.00 [69.00, 83.00] | 0.074 | 77.00 [70.00, 84.00] | 78.00 [70.00, 82.00] | 0.847 |
| ALA_value (median [IQR]) | | 17.00 [12.00, 27.00] | 17.00 [12.00, 27.00] | 16.00 [12.00, 27.00] | 0.508 | 17.00 [12.00, 27.00] | 17.00 [12.00, 28.25] | 0.674 |
| ASA_value (median [IQR]) | | 19.00 [16.00, 25.00] | 19.00 [16.00, 25.00] | 19.00 [16.00, 24.00] | 0.068 | 19.00 [16.00, 25.00] | 18.50 [15.00, 26.00] | 0.49 |
| CK_value (median [IQR]) | | 65.00 [47.00, 93.00] | 65.00 [47.00, 93.00] | 64.00 [48.00, 95.00] | 0.732 | 65.00 [47.00, 92.75] | 81.50 [51.75, 121.50] | 0.008 |
| LD_value (median [IQR]) | | 155.00 [136.00, 179.00] | 155.00 [136.00, 179.00] | 150.00 [135.00, 173.25] | 0.018 | 155.00 [136.00, 179.00] | 153.00 [130.50, 179.00] | 0.397 |
| urea_value (median [IQR]) | | 4.63 [3.72, 5.70] | 4.64 [3.73, 5.70] | 4.50 [3.63, 5.40] | 0.014 | 4.63 [3.72, 5.70] | 4.61 [3.88, 5.57] | 0.79 |
| TB_value (median [IQR]) | | 9.80 [7.30, 13.20] | 9.80 [7.30, 13.20] | 9.15 [6.77, 12.30] | 0.008 | 9.80 [7.30, 13.20] | 10.20 [7.72, 13.55] | 0.339 |
| DB_value (median [IQR]) | | 3.40 [2.50, 4.60] | 3.40 [2.50, 4.60] | 3.10 [2.30, 4.23] | 0.001 | 3.40 [2.50, 4.60] | 3.85 [2.80, 4.62] | 0.12 |
| IDB_value (median [IQR]) | | 6.30 [4.60, 8.70] | 6.30 [4.60, 8.78] | 5.90 [4.20, 8.22] | 0.048 | 6.30 [4.60, 8.70] | 6.60 [5.20, 8.72] | 0.565 |
| TP_value (median [IQR]) | | 67.30 [63.70, 70.90] | 67.30 [63.70, 70.90] | 66.85 [63.40, 70.23] | 0.13 | 67.30 [63.60, 70.90] | 68.25 [64.30, 73.43] | 0.039 |
| albumin_value (median [IQR]) | | 42.10 [39.40, 44.60] | 42.10 [39.40, 44.70] | 41.55 [39.10, 44.20] | 0.112 | 42.10 [39.40, 44.60] | 43.40 [40.98, 46.07] | 0.004 |
| creatinine_value (median [IQR]) | | 62.50 [54.00, 73.70] | 62.65 [54.00, 73.80] | 61.85 [54.00, 72.00] | 0.457 | 62.50 [54.00, 73.60] | 64.50 [57.00, 74.95] | 0.219 |
| glucose_value (median [IQR]) | | 4.82 [4.47, 5.32] | 4.82 [4.46, 5.32] | 4.80 [4.50, 5.16] | 0.201 | 4.82 [4.47, 5.32] | 4.75 [4.42, 5.26] | 0.342 |
| AP_value (median [IQR]) | | 70.00 [56.00, 86.00] | 70.00 [57.00, 87.00] | 68.00 [53.00, 83.25] | 0.012 | 70.00 [56.25, 87.00] | 68.00 [55.75, 82.25] | 0.684 |
| GT_value (median [IQR]) | | 17.00 [12.00, 29.00] | 17.00 [12.00, 29.00] | 16.00 [11.00, 26.00] | 0.135 | 17.00 [12.00, 29.00] | 15.00 [11.00, 24.25] | 0.114 |
| sodium_value (median [IQR]) | | 141.70 [140.00, 143.30] | 141.70 [140.00, 143.30] | 141.65 [140.00, 143.00] | 0.603 | 141.70 [140.00, 143.30] | 141.65 [140.00, 143.50] | 0.876 |
| potassium_value (median [IQR]) | | 3.99 [3.77, 4.21] | 3.99 [3.77, 4.21] | 3.97 [3.78, 4.21] | 0.488 | 3.99 [3.77, 4.21] | 3.96 [3.75, 4.15] | 0.457 |
| chlorine_value (median [IQR]) | | 104.10 [102.00, 106.40] | 104.10 [102.00, 106.40] | 105.05 [103.00, 107.20] | <0.001 | 104.10 [102.00, 106.40] | 104.35 [102.68, 106.78] | 0.458 |
| globulin_value (median [IQR]) | | 25.10 [22.70, 27.70] | 25.10 [22.70, 27.80] | 25.20 [22.80, 27.50] | 0.967 | 25.10 [22.70, 27.70] | 24.65 [22.55, 27.82] | 0.88 |
| WBR_value (median [IQR]) | | 1.68 [1.49, 1.88] | 1.68 [1.49, 1.88] | 1.65 [1.50, 1.85] | 0.594 | 1.68 [1.49, 1.88] | 1.77 [1.52, 1.95] | 0.084 |
| UA_value (median [IQR]) | | 281.00 [231.00, 343.00] | 281.00 [231.00, 343.00] | 281.50 [230.75, 355.00] | 0.57 | 281.00 [231.00, 343.00] | 308.50 [242.25, 382.00] | 0.095 |
| HD_value (median [IQR]) | | 123.00 [108.00, 142.00] | 123.00 [108.00, 143.00] | 121.00 [108.00, 138.00] | 0.088 | 123.00 [108.00, 142.00] | 122.50 [102.75, 142.25] | 0.438 |
| cholesterol_value (median [IQR]) | | 4.24 [3.65, 4.93] | 4.24 [3.65, 4.92] | 4.25 [3.67, 4.97] | 0.612 | 4.24 [3.65, 4.93] | 3.95 [3.56, 4.41] | 0.007 |
| HDL_value (median [IQR]) | | 1.34 [1.11, 1.62] | 1.34 [1.11, 1.62] | 1.41 [1.15, 1.64] | 0.012 | 1.34 [1.11, 1.62] | 1.35 [1.14, 1.51] | 0.882 |
| LDL_value (median [IQR]) | | 2.35 [1.89, 2.91] | 2.35 [1.89, 2.91] | 2.28 [1.88, 2.85] | 0.516 | 2.35 [1.89, 2.91] | 2.29 [1.75, 2.55] | 0.042 |
| cystatinC_value (median [IQR]) | | 0.85 [0.77, 0.97] | 0.86 [0.77, 0.97] | 0.83 [0.75, 0.92] | <0.001 | 0.86 [0.77, 0.97] | 0.80 [0.73, 0.92] | 0.026 |
| calcium_value (median [IQR]) | | 2.24 [2.16, 2.32] | 2.24 [2.16, 2.32] | 2.22 [2.15, 2.29] | 0.004 | 2.24 [2.16, 2.32] | 2.27 [2.19, 2.36] | 0.02 |
| magnesium_value (median [IQR]) | | 0.88 [0.83, 0.94] | 0.88 [0.83, 0.94] | 0.88 [0.82, 0.93] | 0.239 | 0.88 [0.83, 0.94] | 0.88 [0.83, 0.94] | 0.724 |
| SIP_value (median [IQR]) | | 1.17 [1.02, 1.31] | 1.16 [1.02, 1.31] | 1.20 [1.06, 1.34] | 0.008 | 1.16 [1.02, 1.31] | 1.25 [1.08, 1.36] | 0.017 |
| CO2CP_value (median [IQR]) | | 24.30 [22.40, 26.40] | 24.30 [22.40, 26.40] | 23.95 [22.10, 25.90] | 0.02 | 24.30 [22.40, 26.40] | 24.45 [22.65, 26.23] | 0.859 |
| AG_value (median [IQR]) | | 17.10 [14.70, 19.40] | 17.10 [14.70, 19.40] | 16.40 [14.10, 18.80] | 0.001 | 17.10 [14.70, 19.40] | 16.70 [14.07, 18.70] | 0.306 |
| RBC_CV_value (median [IQR]) | | 13.30 [12.80, 14.10] | 13.30 [12.80, 14.10] | 13.20 [12.70, 14.00] | 0.045 | 13.30 [12.80, 14.10] | 13.10 [12.40, 13.60] | 0.001 |
| RBC_SD_value (median [IQR]) | | 44.80 [42.40, 47.60] | 44.80 [42.40, 47.60] | 44.60 [42.40, 47.20] | 0.358 | 44.80 [42.40, 47.60] | 44.10 [41.27, 46.10] | 0.029 |
| WBCC_value (median [IQR]) | | 5.60 [4.64, 6.82] | 5.60 [4.63, 6.83] | 5.68 [4.71, 6.78] | 0.882 | 5.60 [4.64, 6.82] | 5.43 [4.67, 7.25] | 0.743 |
| POM_value (median [IQR]) | | 6.30 [5.20, 7.70] | 6.30 [5.20, 7.70] | 6.15 [5.10, 7.32] | 0.034 | 6.30 [5.20, 7.70] | 6.55 [5.07, 8.33] | 0.388 |
| AOM_value (median [IQR]) | | 0.36 [0.27, 0.46] | 0.36 [0.27, 0.46] | 0.35 [0.26, 0.45] | 0.214 | 0.36 [0.27, 0.46] | 0.37 [0.29, 0.47] | 0.368 |
| RBCC_value (median [IQR]) | | 4.38 [4.04, 4.76] | 4.38 [4.04, 4.76] | 4.32 [4.05, 4.69] | 0.227 | 4.38 [4.04, 4.75] | 4.52 [4.21, 4.85] | 0.026 |
| hematocrit_value (median [IQR]) | | 0.40 [0.37, 0.43] | 0.40 [0.37, 0.43] | 0.40 [0.38, 0.42] | 0.61 | 0.40 [0.37, 0.43] | 0.42 [0.39, 0.44] | 0.003 |
| POL_value (median [IQR]) | | 30.60 [23.90, 37.50] | 30.60 [23.90, 37.50] | 30.80 [24.90, 36.92] | 0.749 | 30.60 [23.90, 37.50] | 33.10 [22.90, 37.70] | 0.477 |
| AOL_value (median [IQR]) | | 1.66 [1.31, 2.08] | 1.66 [1.31, 2.08] | 1.67 [1.32, 2.14] | 0.45 | 1.66 [1.31, 2.08] | 1.68 [1.39, 2.04] | 0.443 |
| ARBC_HGB_value (median [IQR]) | | 30.30 [29.20, 31.30] | 30.30 [29.20, 31.30] | 30.20 [29.20, 31.22] | 0.927 | 30.30 [29.20, 31.30] | 30.40 [29.75, 31.07] | 0.505 |
| ARBC_HGB_con_value (median [IQR]) | | 327.00 [320.00, 334.00] | 327.00 [320.00, 334.75] | 327.00 [320.00, 333.00] | 0.123 | 327.00 [320.00, 334.00] | 327.00 [321.00, 334.25] | 0.803 |
| ARBCV_value (median [IQR]) | | 92.20 [89.10, 95.20] | 92.20 [89.10, 95.20] | 92.30 [89.38, 95.70] | 0.338 | 92.20 [89.10, 95.20] | 92.70 [90.15, 94.98] | 0.505 |
| POB_value (median [IQR]) | | 0.40 [0.20, 0.60] | 0.40 [0.20, 0.60] | 0.40 [0.30, 0.60] | 0.975 | 0.40 [0.20, 0.60] | 0.50 [0.30, 0.70] | 0.251 |
| POE_value (median [IQR]) | | 1.90 [1.10, 3.00] | 1.90 [1.10, 3.00] | 1.90 [1.20, 3.00] | 0.576 | 1.90 [1.10, 3.00] | 1.75 [0.90, 2.52] | 0.166 |
| hemoglobin_value (median [IQR]) | | 131.00 [121.00, 143.00] | 131.00 [121.00, 143.00] | 130.00 [122.00, 139.00] | 0.297 | 131.00 [121.00, 143.00] | 136.00 [127.00, 146.25] | 0.005 |
| PC_value (median [IQR]) | | 189.00 [149.00, 233.00] | 189.00 [149.00, 234.00] | 193.50 [156.00, 231.00] | 0.444 | 189.00 [149.00, 233.00] | 187.00 [152.50, 225.00] | 0.798 |
| PON_value (median [IQR]) | | 59.70 [52.40, 67.20] | 59.70 [52.32, 67.20] | 60.10 [53.27, 66.50] | 0.815 | 59.70 [52.40, 67.20] | 57.55 [51.67, 68.73] | 0.608 |
| AON_value (median [IQR]) | | 3.27 [2.53, 4.30] | 3.26 [2.53, 4.30] | 3.29 [2.57, 4.23] | 0.914 | 3.27 [2.53, 4.30] | 3.28 [2.64, 4.37] | 0.799 |
| SUM (median [IQR]) | | 516.00 [380.00, 728.00] | 515.00 [379.00, 727.00] | 583.50 [412.75, 776.00] | 0.001 | 516.00 [380.00, 728.00] | 541.00 [384.25, 759.75] | 0.775 |
| ADP_SUM (median [IQR]) | | 19.00 [10.00, 27.00] | 19.00 [10.00, 27.00] | 24.00 [17.00, 33.25] | <0.001 | 19.00 [10.00, 27.00] | 21.50 [12.00, 29.25] | 0.083 |
| AMTL (median [IQR]) | | 0.00 [0.00, 0.00] | 0.00 [0.00, 0.00] | 0.00 [0.00, 0.00] | 0.079 | 0.00 [0.00, 0.00] | 0.00 [0.00, 0.00] | 0.261 |
| ASXTPL (median [IQR]) | | 0.00 [0.00, 0.00] | 0.00 [0.00, 0.00] | 0.00 [0.00, 0.00] | 0.003 | 0.00 [0.00, 0.00] | 0.00 [0.00, 0.00] | 0.283 |
| BMQ (median [IQR]) | | 0.00 [0.00, 0.00] | 0.00 [0.00, 0.00] | 0.00 [0.00, 0.00] | 0.703 | 0.00 [0.00, 0.00] | 0.00 [0.00, 0.00] | 0.871 |
| DLXT (median [IQR]) | | 0.00 [0.00, 0.00] | 0.00 [0.00, 0.00] | 0.00 [0.00, 0.00] | 0.54 | 0.00 [0.00, 0.00] | 0.00 [0.00, 0.00] | 0.462 |
| DSP (median [IQR]) | | 0.00 [0.00, 0.00] | 0.00 [0.00, 0.00] | 0.00 [0.00, 0.00] | 0.062 | 0.00 [0.00, 0.00] | 0.00 [0.00, 0.00] | 0.334 |
| FFSM (median [IQR]) | | 0.00 [0.00, 0.00] | 0.00 [0.00, 0.00] | 0.00 [0.00, 0.00] | 0.953 | 0.00 [0.00, 0.00] | 0.00 [0.00, 0.00] | 0.282 |
| FPSDMLQX (median [IQR]) | | 0.00 [0.00, 0.00] | 0.00 [0.00, 0.00] | 0.00 [0.00, 0.00] | 0.59 | 0.00 [0.00, 0.00] | 0.00 [0.00, 0.00] | 0.818 |
| FXT (median [IQR]) | | 0.00 [0.00, 0.00] | 0.00 [0.00, 0.00] | 0.00 [0.00, 0.00] | 0.925 | 0.00 [0.00, 0.00] | 0.00 [0.00, 0.00] | 0.077 |
| LMPM (median [IQR]) | | 0.00 [0.00, 0.00] | 0.00 [0.00, 0.00] | 0.00 [0.00, 0.00] | <0.001 | 0.00 [0.00, 0.00] | 0.00 [0.00, 0.00] | 0.032 |
| MDP (median [IQR]) | | 0.00 [0.00, 0.00] | 0.00 [0.00, 0.00] | 0.00 [0.00, 0.00] | 0.259 | 0.00 [0.00, 0.00] | 0.00 [0.00, 0.00] | 0.461 |
| MPTL (median [IQR]) | | 0.00 [0.00, 0.00] | 0.00 [0.00, 0.00] | 0.00 [0.00, 0.00] | 0.703 | 0.00 [0.00, 0.00] | 0.00 [0.00, 0.00] | 0.871 |
| PLXT (median [IQR]) | | 0.00 [0.00, 0.00] | 0.00 [0.00, 0.00] | 0.00 [0.00, 4.25] | <0.001 | 0.00 [0.00, 0.00] | 0.00 [0.00, 0.00] | 0.587 |
| SQL (median [IQR]) | | 0.00 [0.00, 6.00] | 0.00 [0.00, 6.00] | 0.00 [0.00, 0.50] | 0.269 | 0.00 [0.00, 6.00] | 0.00 [0.00, 15.25] | 0.167 |
| XTPL (median [IQR]) | | 0.00 [0.00, 0.00] | 0.00 [0.00, 0.00] | 0.00 [0.00, 0.00] | 0.896 | 0.00 [0.00, 0.00] | 0.00 [0.00, 0.00] | 0.173 |
| YSQZT (median [IQR]) | | 0.00 [0.00, 0.00] | 0.00 [0.00, 0.00] | 0.00 [0.00, 0.00] | 0.203 | 0.00 [0.00, 0.00] | 0.00 [0.00, 0.00] | 0.69 |
| YSWLFX (median [IQR]) | | 0.00 [0.00, 3.00] | 0.00 [0.00, 2.00] | 0.00 [0.00, 18.00] | <0.001 | 0.00 [0.00, 3.00] | 0.00 [0.00, 1.00] | 0.684 |
| AP_SUM (median [IQR]) | | 10.00 [0.00, 21.00] | 10.00 [0.00, 21.00] | 18.00 [5.00, 27.25] | <0.001 | 10.00 [0.00, 21.00] | 20.00 [9.00, 38.75] | <0.001 |
| ADanP (median [IQR]) | | 0.00 [0.00, 12.00] | 0.00 [0.00, 11.00] | 0.00 [0.00, 16.00] | 0.001 | 0.00 [0.00, 12.00] | 0.50 [0.00, 22.00] | 0.002 |
| AHBL (median [IQR]) | | 0.00 [0.00, 0.00] | 0.00 [0.00, 0.00] | 0.00 [0.00, 0.00] | 0.006 | 0.00 [0.00, 0.00] | 0.00 [0.00, 0.00] | <0.001 |
| ALPZ (median [IQR]) | | 0.00 [0.00, 0.00] | 0.00 [0.00, 0.00] | 0.00 [0.00, 0.00] | 0.487 | 0.00 [0.00, 0.00] | 0.00 [0.00, 0.00] | 0.036 |
| FMSZLP (median [IQR]) | | 0.00 [0.00, 3.00] | 0.00 [0.00, 3.00] | 0.00 [0.00, 15.00] | <0.001 | 0.00 [0.00, 3.00] | 0.00 [0.00, 3.25] | 0.83 |
| FPDC (median [IQR]) | | 0.00 [0.00, 0.00] | 0.00 [0.00, 0.00] | 0.00 [0.00, 0.00] | 0.555 | 0.00 [0.00, 0.00] | 0.00 [0.00, 0.00] | 0.801 |
| FPDCZSY (median [IQR]) | | 0.00 [0.00, 0.00] | 0.00 [0.00, 0.00] | 0.00 [0.00, 0.00] | 0.988 | 0.00 [0.00, 0.00] | 0.00 [0.00, 0.00] | 0.408 |
| LBL (median [IQR]) | | 0.00 [0.00, 0.00] | 0.00 [0.00, 0.00] | 0.00 [0.00, 0.00] | 0.516 | 0.00 [0.00, 0.00] | 0.00 [0.00, 0.00] | 0.758 |
| LBQ (median [IQR]) | | 0.00 [0.00, 0.00] | 0.00 [0.00, 0.00] | 0.00 [0.00, 0.00] | 0.495 | 0.00 [0.00, 0.00] | 0.00 [0.00, 0.00] | 0.771 |
| LDP (median [IQR]) | | 0.00 [0.00, 0.00] | 0.00 [0.00, 0.00] | 0.00 [0.00, 0.00] | 0.452 | 0.00 [0.00, 0.00] | 0.00 [0.00, 0.00] | 0.004 |
| LPT (median [IQR]) | | 0.00 [0.00, 0.00] | 0.00 [0.00, 0.00] | 0.00 [0.00, 0.00] | 0.446 | 0.00 [0.00, 0.00] | 0.00 [0.00, 0.00] | <0.001 |
| PLPT (median [IQR]) | | 0.00 [0.00, 0.00] | 0.00 [0.00, 0.00] | 0.00 [0.00, 0.00] | 0.008 | 0.00 [0.00, 0.00] | 0.00 [0.00, 0.00] | <0.001 |
| SBL (median [IQR]) | | 0.00 [0.00, 0.00] | 0.00 [0.00, 0.00] | 0.00 [0.00, 0.00] | 0.054 | 0.00 [0.00, 0.00] | 0.00 [0.00, 0.00] | <0.001 |
| SBLZSY (median [IQR]) | | 0.00 [0.00, 0.00] | 0.00 [0.00, 0.00] | 0.00 [0.00, 0.00] | 0.848 | 0.00 [0.00, 0.00] | 0.00 [0.00, 0.00] | 0.003 |
| YSQLXT (median [IQR]) | | 0.00 [0.00, 0.00] | 0.00 [0.00, 0.00] | 0.00 [0.00, 0.00] | 0.59 | 0.00 [0.00, 0.00] | 0.00 [0.00, 0.00] | 0.818 |
| AA_SUM (median [IQR]) | | 20.00 [5.00, 36.00] | 20.00 [4.00, 36.00] | 30.00 [17.00, 47.25] | <0.001 | 20.00 [5.00, 36.00] | 16.50 [0.00, 35.00] | 0.224 |
| APZL (median [IQR]) | | 1.00 [0.00, 18.00] | 1.00 [0.00, 18.00] | 2.00 [0.00, 23.00] | 0.034 | 1.00 [0.00, 18.00] | 2.50 [0.00, 16.25] | 0.881 |
| ASZL (median [IQR]) | | 0.00 [0.00, 0.00] | 0.00 [0.00, 0.00] | 0.00 [0.00, 0.00] | 0.232 | 0.00 [0.00, 0.00] | 0.00 [0.00, 0.00] | 0.403 |
| DLHT (median [IQR]) | | 0.00 [0.00, 0.00] | 0.00 [0.00, 0.00] | 0.00 [0.00, 0.00] | 0.2 | 0.00 [0.00, 0.00] | 0.00 [0.00, 0.00] | 0.432 |
| DXP (median [IQR]) | | 0.00 [0.00, 0.00] | 0.00 [0.00, 0.00] | 0.00 [0.00, 0.00] | 0.024 | 0.00 [0.00, 0.00] | 0.00 [0.00, 0.00] | 0.589 |
| LLXZ (median [IQR]) | | 0.00 [0.00, 0.00] | 0.00 [0.00, 0.00] | 0.00 [0.00, 1.00] | <0.001 | 0.00 [0.00, 0.00] | 0.00 [0.00, 0.00] | 0.316 |
| LXXP (median [IQR]) | | 0.00 [0.00, 13.00] | 0.00 [0.00, 13.00] | 8.00 [0.00, 24.25] | <0.001 | 0.00 [0.00, 13.00] | 0.00 [0.00, 11.25] | 0.966 |
| MDZL (median [IQR]) | | 0.00 [0.00, 0.00] | 0.00 [0.00, 0.00] | 0.00 [0.00, 0.00] | 0.588 | 0.00 [0.00, 0.00] | 0.00 [0.00, 0.00] | 0.573 |
| MDZLZSY (median [IQR]) | | 0.00 [0.00, 0.00] | 0.00 [0.00, 0.00] | 0.00 [0.00, 0.00] | 0.043 | 0.00 [0.00, 0.00] | 0.00 [0.00, 0.00] | 0.324 |
| TDLTJL (median [IQR]) | | 0.00 [0.00, 0.00] | 0.00 [0.00, 0.00] | 0.00 [0.00, 0.00] | 0.548 | 0.00 [0.00, 0.00] | 0.00 [0.00, 0.00] | 0.396 |
| XXP (median [IQR]) | | 0.00 [0.00, 0.00] | 0.00 [0.00, 0.00] | 0.00 [0.00, 0.00] | 0.609 | 0.00 [0.00, 0.00] | 0.00 [0.00, 0.00] | 0.827 |
| YZPKL (median [IQR]) | | 0.00 [0.00, 0.00] | 0.00 [0.00, 0.00] | 0.00 [0.00, 0.00] | 0.169 | 0.00 [0.00, 0.00] | 0.00 [0.00, 0.00] | 0.237 |
| MSB_SUM (median [IQR]) | | 0.00 [0.00, 0.00] | 0.00 [0.00, 0.00] | 0.00 [0.00, 0.00] | <0.001 | 0.00 [0.00, 0.00] | 0.00 [0.00, 0.00] | 0.931 |
| BWSN (median [IQR]) | | 0.00 [0.00, 0.00] | 0.00 [0.00, 0.00] | 0.00 [0.00, 0.00] | <0.001 | 0.00 [0.00, 0.00] | 0.00 [0.00, 0.00] | 0.675 |
| JBPD (median [IQR]) | | 0.00 [0.00, 0.00] | 0.00 [0.00, 0.00] | 0.00 [0.00, 0.00] | 0.394 | 0.00 [0.00, 0.00] | 0.00 [0.00, 0.00] | 0.716 |
| KMXP (median [IQR]) | | 0.00 [0.00, 0.00] | 0.00 [0.00, 0.00] | 0.00 [0.00, 0.00] | 0.557 | 0.00 [0.00, 0.00] | 0.00 [0.00, 0.00] | 0.745 |
| LMSZ (median [IQR]) | | 0.00 [0.00, 0.00] | 0.00 [0.00, 0.00] | 0.00 [0.00, 0.00] | 0.122 | 0.00 [0.00, 0.00] | 0.00 [0.00, 0.00] | 0.983 |
| TBZ (median [IQR]) | | 0.00 [0.00, 0.00] | 0.00 [0.00, 0.00] | 0.00 [0.00, 0.00] | 0.567 | 0.00 [0.00, 0.00] | 0.00 [0.00, 0.00] | 0.45 |
| TSZ (median [IQR]) | | 0.00 [0.00, 0.00] | 0.00 [0.00, 0.00] | 0.00 [0.00, 0.00] | 0.001 | 0.00 [0.00, 0.00] | 0.00 [0.00, 0.00] | 0.755 |
| ASE_SUM (median [IQR]) | | 0.00 [0.00, 7.00] | 0.00 [0.00, 7.00] | 0.00 [0.00, 9.00] | 0.909 | 0.00 [0.00, 7.00] | 0.50 [0.00, 18.25] | 0.004 |
| ASPL (median [IQR]) | | 0.00 [0.00, 0.00] | 0.00 [0.00, 0.00] | 0.00 [0.00, 0.00] | 0.022 | 0.00 [0.00, 0.00] | 0.00 [0.00, 0.00] | 0.04 |
| ATFTTG (median [IQR]) | | 0.00 [0.00, 0.00] | 0.00 [0.00, 0.00] | 0.00 [0.00, 0.00] | <0.001 | 0.00 [0.00, 0.00] | 0.00 [0.00, 0.00] | 0.11 |
| BHS (median [IQR]) | | 0.00 [0.00, 0.00] | 0.00 [0.00, 0.00] | 0.00 [0.00, 0.00] | 0.01 | 0.00 [0.00, 0.00] | 0.00 [0.00, 10.25] | <0.001 |
| BSKD (median [IQR]) | | 0.00 [0.00, 0.00] | 0.00 [0.00, 0.00] | 0.00 [0.00, 0.00] | 0.001 | 0.00 [0.00, 0.00] | 0.00 [0.00, 0.00] | 0.058 |
| JYEC (median [IQR]) | | 0.00 [0.00, 0.00] | 0.00 [0.00, 0.00] | 0.00 [0.00, 0.00] | 0.431 | 0.00 [0.00, 0.00] | 0.00 [0.00, 0.00] | 0.96 |
| KSL (median [IQR]) | | 0.00 [0.00, 0.00] | 0.00 [0.00, 0.00] | 0.00 [0.00, 0.00] | 0.722 | 0.00 [0.00, 0.00] | 0.00 [0.00, 0.00] | 0.777 |
| MR (median [IQR]) | | 0.00 [0.00, 0.00] | 0.00 [0.00, 0.00] | 0.00 [0.00, 0.00] | 0.564 | 0.00 [0.00, 0.00] | 0.00 [0.00, 0.00] | 0.466 |
| MSBL (median [IQR]) | | 0.00 [0.00, 0.00] | 0.00 [0.00, 0.00] | 0.00 [0.00, 0.00] | 0.078 | 0.00 [0.00, 0.00] | 0.00 [0.00, 0.00] | 0.185 |
| HYP_SUM (median [IQR]) | | 0.00 [0.00, 0.00] | 0.00 [0.00, 0.00] | 0.00 [0.00, 0.00] | 0.155 | 0.00 [0.00, 0.00] | 0.00 [0.00, 0.00] | 0.761 |
| ZPKL (median [IQR]) | | 0.00 [0.00, 0.00] | 0.00 [0.00, 0.00] | 0.00 [0.00, 0.00] | 0.865 | 0.00 [0.00, 0.00] | 0.00 [0.00, 0.00] | 0.942 |
| ZZT (median [IQR]) | | 0.00 [0.00, 0.00] | 0.00 [0.00, 0.00] | 0.00 [0.00, 0.00] | 0.153 | 0.00 [0.00, 0.00] | 0.00 [0.00, 0.00] | 0.763 |
| OT_SUM (median [IQR]) | | 0.00 [0.00, 0.00] | 0.00 [0.00, 0.00] | 0.00 [0.00, 0.25] | <0.001 | 0.00 [0.00, 0.00] | 0.00 [0.00, 0.00] | 0.95 |
| BSLE (median [IQR]) | | 0.00 [0.00, 0.00] | 0.00 [0.00, 0.00] | 0.00 [0.00, 0.00] | 0.41 | 0.00 [0.00, 0.00] | 0.00 [0.00, 0.00] | 0.52 |
| HPSMTLE (median [IQR]) | | 0.00 [0.00, 0.00] | 0.00 [0.00, 0.00] | 0.00 [0.00, 0.00] | 0.172 | 0.00 [0.00, 0.00] | 0.00 [0.00, 0.00] | 0.419 |
| JSSMTLE (median [IQR]) | | 0.00 [0.00, 0.00] | 0.00 [0.00, 0.00] | 0.00 [0.00, 0.00] | 0.058 | 0.00 [0.00, 0.00] | 0.00 [0.00, 0.00] | 0.372 |
| PNLE (median [IQR]) | | 0.00 [0.00, 0.00] | 0.00 [0.00, 0.00] | 0.00 [0.00, 0.00] | <0.001 | 0.00 [0.00, 0.00] | 0.00 [0.00, 0.00] | 0.424 |
| ZJZXSN (median [IQR]) | | 0.00 [0.00, 0.00] | 0.00 [0.00, 0.00] | 0.00 [0.00, 0.00] | 0.516 | 0.00 [0.00, 0.00] | 0.00 [0.00, 0.00] | 0.985 |
| GWS (median [IQR]) | | 0.00 [0.00, 0.00] | 0.00 [0.00, 0.00] | 0.00 [0.00, 0.00] | 0.423 | 0.00 [0.00, 0.00] | 0.00 [0.00, 0.00] | 0.018 |
| PHY_SUM (median [IQR]) | | 0.00 [0.00, 26.00] | 0.00 [0.00, 26.00] | 9.00 [0.00, 34.25] | <0.001 | 0.00 [0.00, 26.00] | 0.00 [0.00, 5.50] | 0.04 |
| MPBT (median [IQR]) | | 0.00 [0.00, 0.00] | 0.00 [0.00, 0.00] | 0.00 [0.00, 0.00] | 0.33 | 0.00 [0.00, 0.00] | 0.00 [0.00, 0.00] | 0.226 |
| ME (median [IQR]) | | 0.00 [0.00, 0.00] | 0.00 [0.00, 0.00] | 0.00 [0.00, 0.00] | 0.044 | 0.00 [0.00, 0.00] | 0.00 [0.00, 0.00] | 0.016 |
| TMS (median [IQR]) | | 0.00 [0.00, 0.00] | 0.00 [0.00, 0.00] | 0.00 [0.00, 0.00] | 0.003 | 0.00 [0.00, 0.00] | 0.00 [0.00, 0.00] | 0.421 |
| TRMS (median [IQR]) | | 0.00 [0.00, 0.00] | 0.00 [0.00, 0.00] | 0.00 [0.00, 0.00] | 0.944 | 0.00 [0.00, 0.00] | 0.00 [0.00, 0.00] | 0.654 |
| EEG (median [IQR]) | | 0.00 [0.00, 22.00] | 0.00 [0.00, 22.00] | 0.00 [0.00, 30.00] | <0.001 | 0.00 [0.00, 22.00] | 0.00 [0.00, 0.00] | <0.001 |
| BT (median [IQR]) | | 0.00 [0.00, 0.00] | 0.00 [0.00, 0.00] | 0.00 [0.00, 0.00] | 0.85 | 0.00 [0.00, 0.00] | 0.00 [0.00, 0.00] | 0.236 |
| PSY (median [IQR]) | | 1.00 [0.00, 4.00] | 1.00 [0.00, 4.00] | 2.00 [0.00, 6.00] | <0.001 | 1.00 [0.00, 4.00] | 1.00 [0.00, 6.25] | 0.088 |

**Supplementary Information SI3:**

**3.1 Conversions to BD/SCZ stratified by some predictors**

Rates of transition from UD to BD were substantially higher for females, while rates of transition from UD to SCZ were lower for females compared to males (Figure S1). Rates of transition from UD to SCZ were substantially higher for patients with family history of mental illness, and rates of transition from UD to BD were slightly higher for patients with family history of mental illness compared to patients without family history of mental illness (Figure S2). Patients with recurrent depression had higher transition rates from UD to BD compared to patients with single-episode depression, while patients with psychotic symptoms had substantially higher transition rates from UD to SCZ compared to patients without psychotic symptoms (Figure S3-S4).

For antidepressants, patients prescribed venlafaxine and paroxetine had higher transition rates from UD to BD, and patients prescribed citalopram and fluoxetine had higher transition rates from UD to SCZ. For antipsychotics, patients prescribed sulpiride and quetiapine had higher transition rates from UD to BD, and patients prescribed risperidone and sulpiride had higher transition rates from UD to SCZ. For mood stabilizers, sodium valproate and lithium carbonate prescribed to patients had higher rates of transition. Clonazepam, diazepam, and lorazepam are three anxiolytics with the highest rates of transition from UD to BD. For β receptor blockers, metoprolol tartrate and propranolol hydrochloride are two drugs with the highest rates of transition from UD to BD. Trihexyphenidyl and bisacodyl are two anti-side effects drugs affected most for transition from UD to BD.


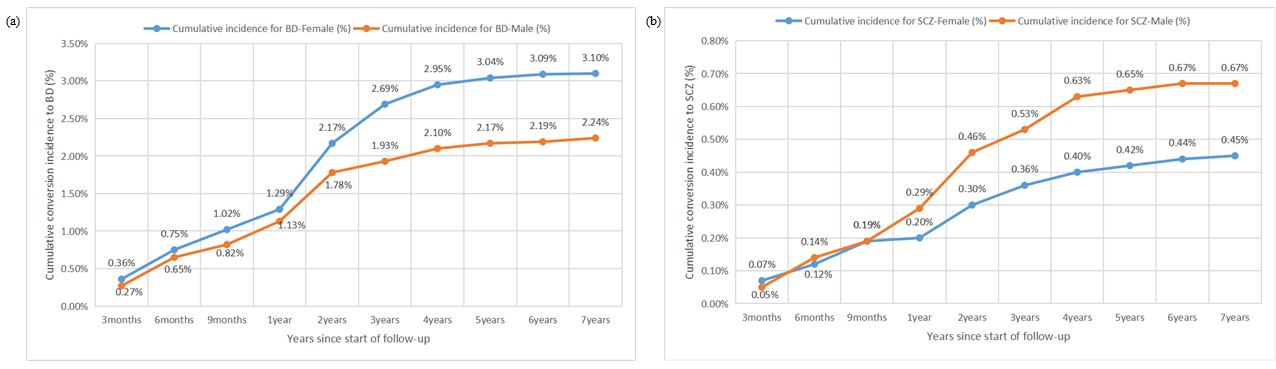


Figure S1 Cumulative conversion incidences and Yearly crude incidence rate for the conversion from UD to BD and SCZ stratified by sex.

**
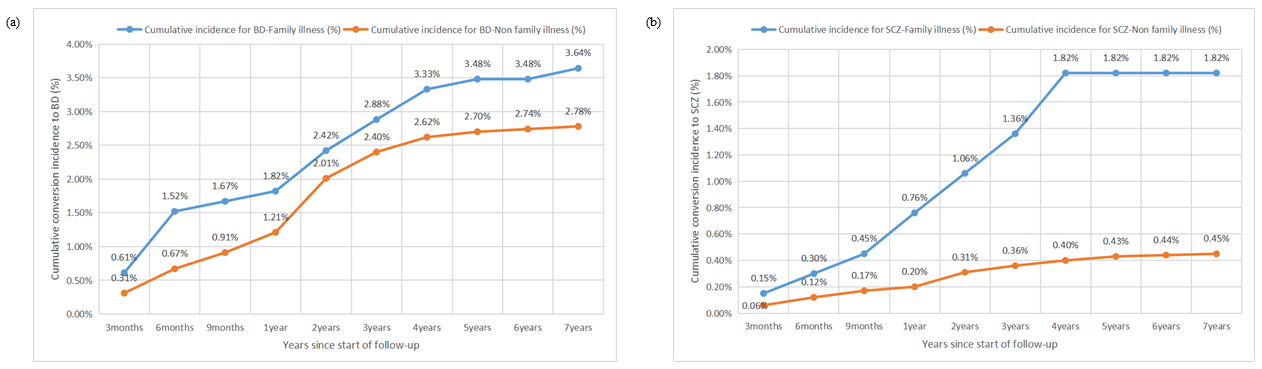
**

Figure S2 Cumulative conversion incidences and Yearly crude incidence rate for the conversion from UD to BD and SCZ stratified by history of family mental illness.

**
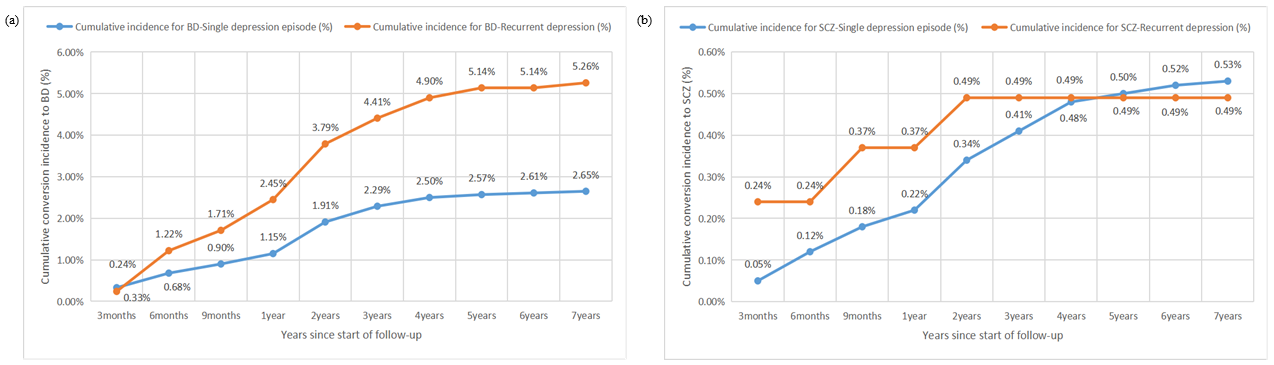
**

Figure S3 Cumulative conversion incidences and Yearly crude incidence rate for the conversion from UD to BD and SCZ stratified by single-episode or recurrent depression.

**
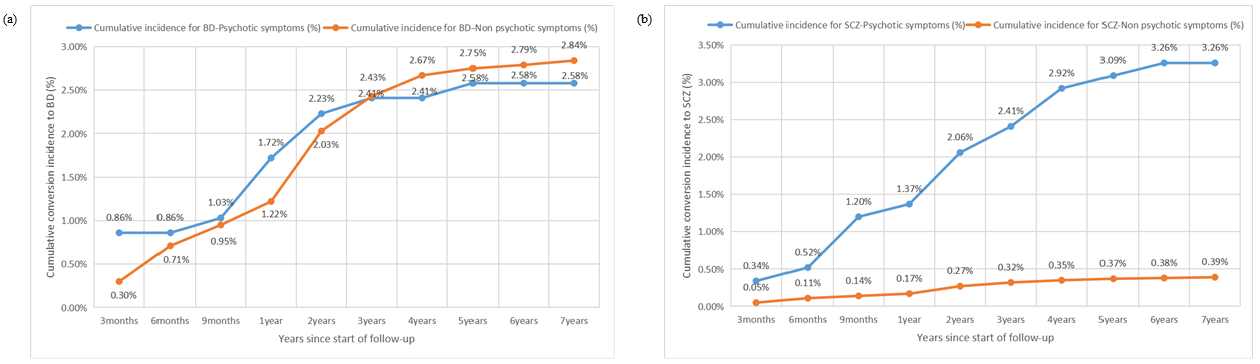
**

Figure S4 Cumulative conversion incidences and Yearly crude incidence rate for the conversion from UD to BD and SCZ stratified by psychotic symptoms.

**Supplementary Information SI4:**

| (A) Using the LASSO method filtered features to predict conversion to BD within 1 year (B) Using the LASSO method filtered features to predict conversion to BD within 2 years  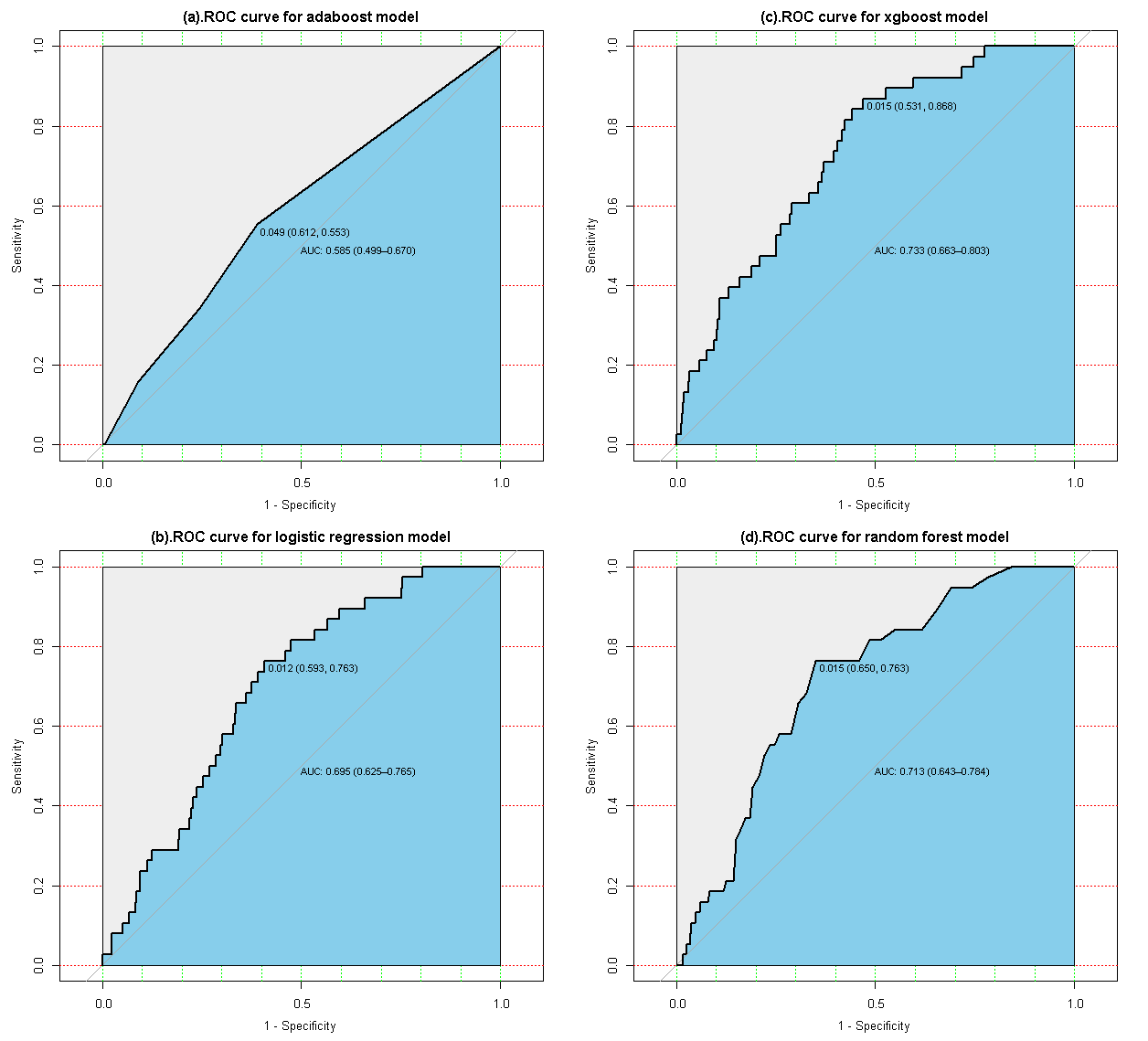 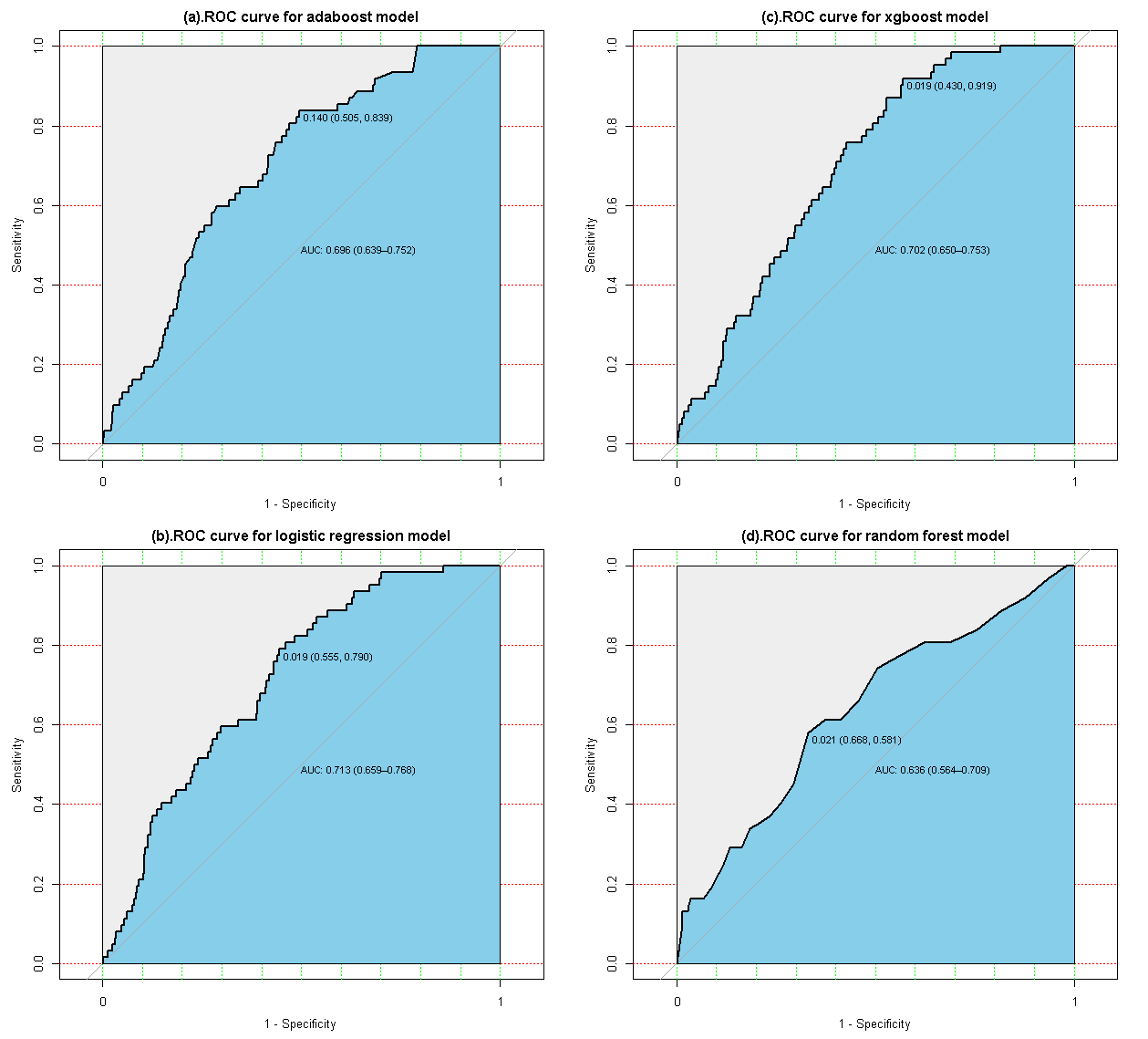   \| Algorithm \| Threshold \| Sensitivity \| Specificity \| PPV \| NPV \| Algorithm \| Threshold \| Sensitivity \| Specificity \| PPV \| NPV \| \| --- \| --- \| --- \| --- \| --- \| --- \| --- \| --- \| --- \| --- \| --- \| --- \| \| adaboost \| 0.049 \| 0.553 \| 0.612 \| 0.018 \| 0.991 \| adaboost \| 0.140 \| 0.839 \| 0.505 \| 0.034 \| 0.993 \| \| logistic regression \| 0.012 \| 0.763 \| 0.593 \| 0.023 \| 0.995 \| logistic regression \| 0.019 \| 0.790 \| 0.555 \| 0.036 \| 0.992 \| \| xgboost \| 0.015 \| 0.868 \| 0.531 \| 0.023 \| 0.997 \| xgboost \| 0.019 \| 0.919 \| 0.430 \| 0.032 \| 0.996 \| \| random forest \| 0.015 \| 0.763 \| 0.650 \| 0.027 \| 0.995 \| random forest \| 0.021 \| 0.581 \| 0.668 \| 0.035 \| 0.987 \|   (C) Using the LASSO method filtered features to predict conversion to BD within 7 years  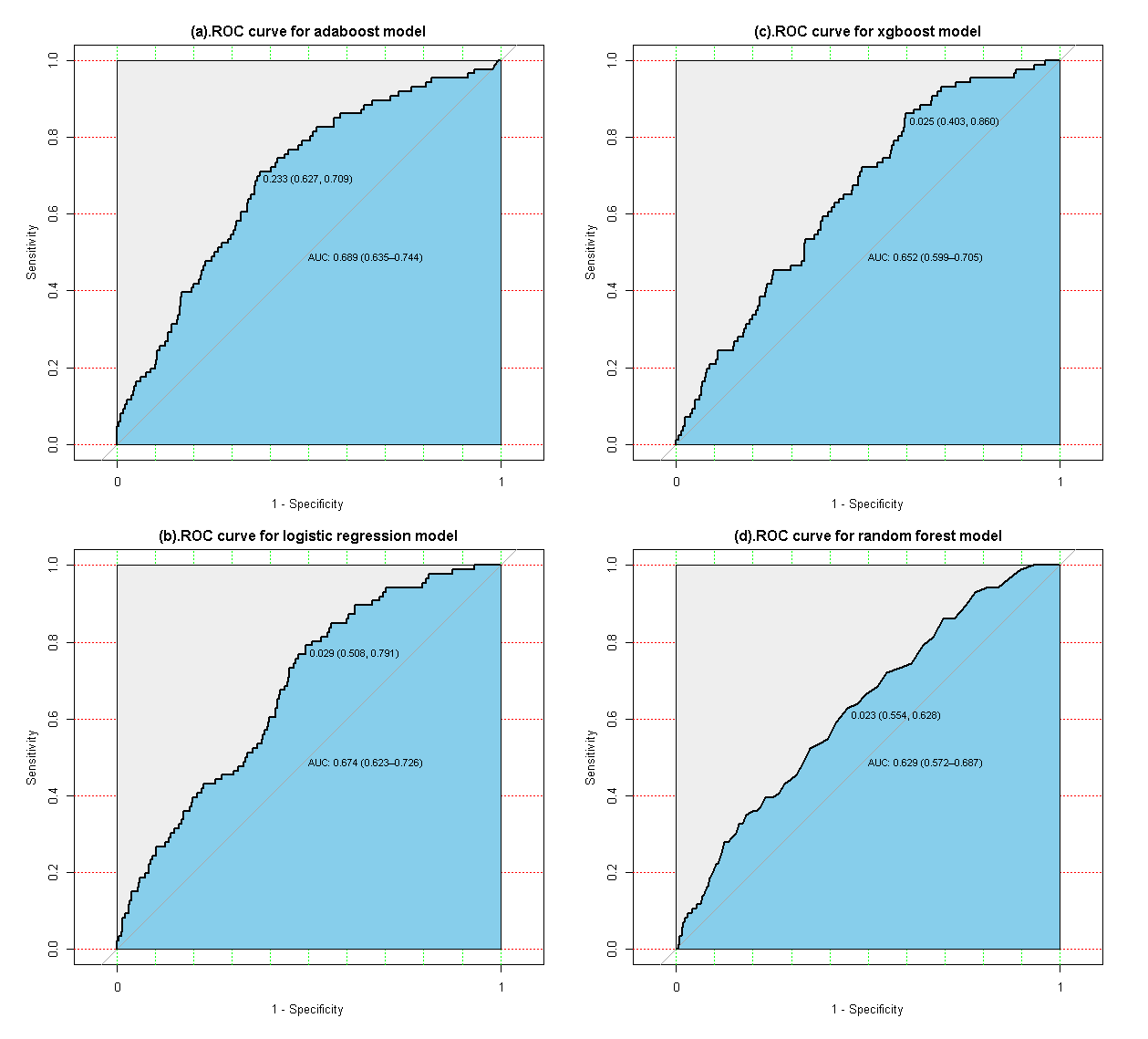   \| Algorithm \| Threshold \| Sensitivity \| Specificity \| PPV \| NPV \| \| --- \| --- \| --- \| --- \| --- \| --- \| \| adaboost \| 0.233 \| 0.709 \| 0.627 \| 0.052 \| 0.987 \| \| logistic regression \| 0.029 \| 0.791 \| 0.508 \| 0.045 \| 0.988 \| \| xgboost \| 0.025 \| 0.860 \| 0.403 \| 0.040 \| 0.990 \| \| random forest \| 0.023 \| 0.628 \| 0.554 \| 0.039 \| 0.980 \| |
| --- | --- | --- | --- | --- | --- | --- | --- | --- | --- | --- | --- | --- | --- | --- | --- | --- | --- | --- | --- | --- | --- | --- | --- | --- | --- | --- | --- | --- | --- | --- | --- | --- | --- | --- | --- | --- | --- | --- | --- | --- | --- | --- | --- | --- | --- | --- | --- | --- | --- | --- | --- | --- | --- | --- | --- | --- | --- | --- | --- | --- | --- | --- | --- | --- | --- | --- | --- | --- | --- | --- | --- | --- | --- | --- | --- | --- | --- | --- | --- | --- | --- | --- | --- | --- | --- | --- | --- | --- | --- | --- |

Figure S5 The performance of prediction models using the LASSO method filtered features for the conversion from UD to BD. The area under the receiver operating characteristic (AUC) ROC curve. The tables showed the internal validation performance (i.e., sensitivity, specificity, positive predictive value [PPV], and negative predictive value [NPV]) of prediction models.

| (A) Using the RF method filtered features to predict conversion to BD within 1 year (B) Using the RF method filtered features to predict conversion to BD within 2 years  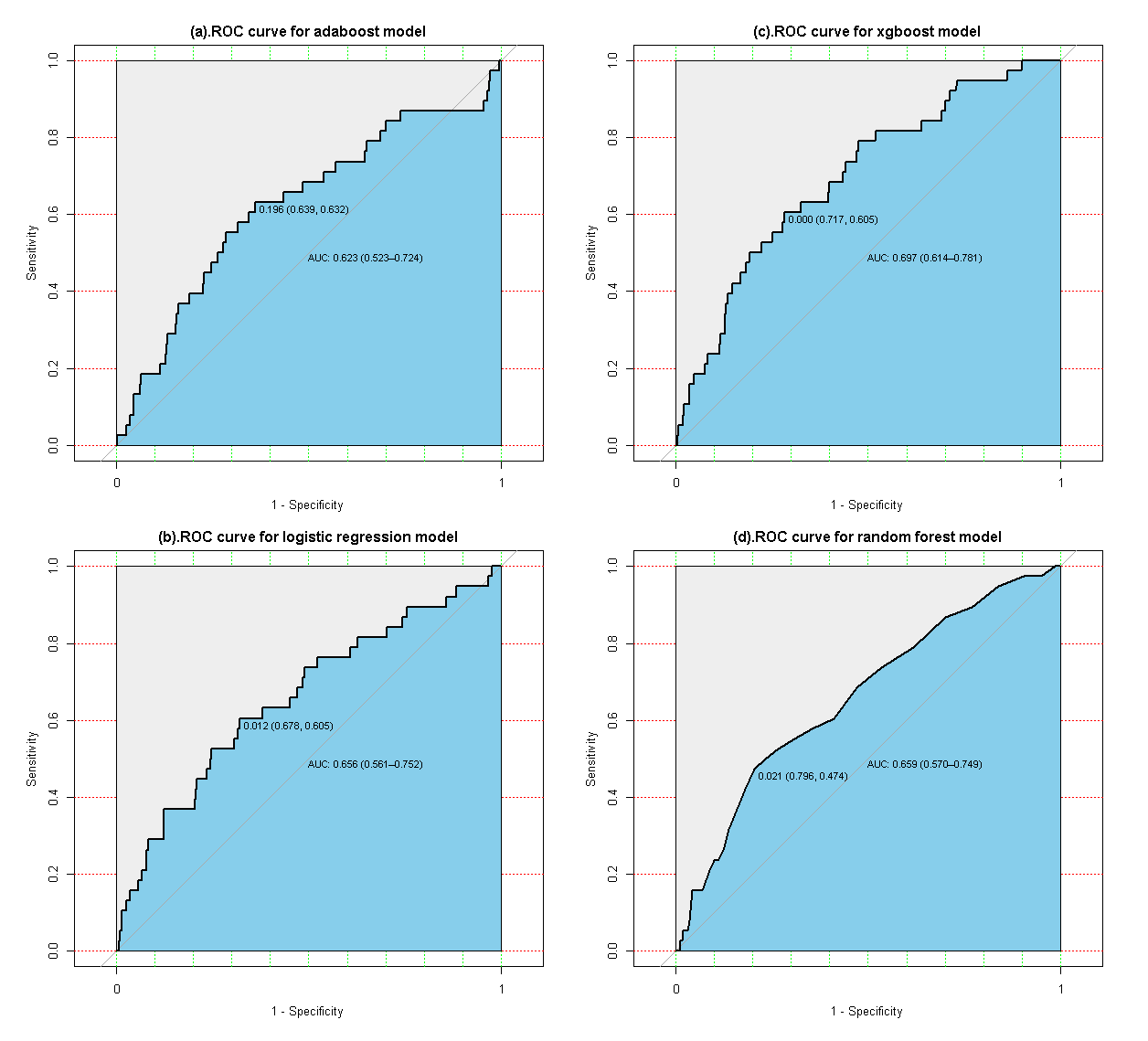 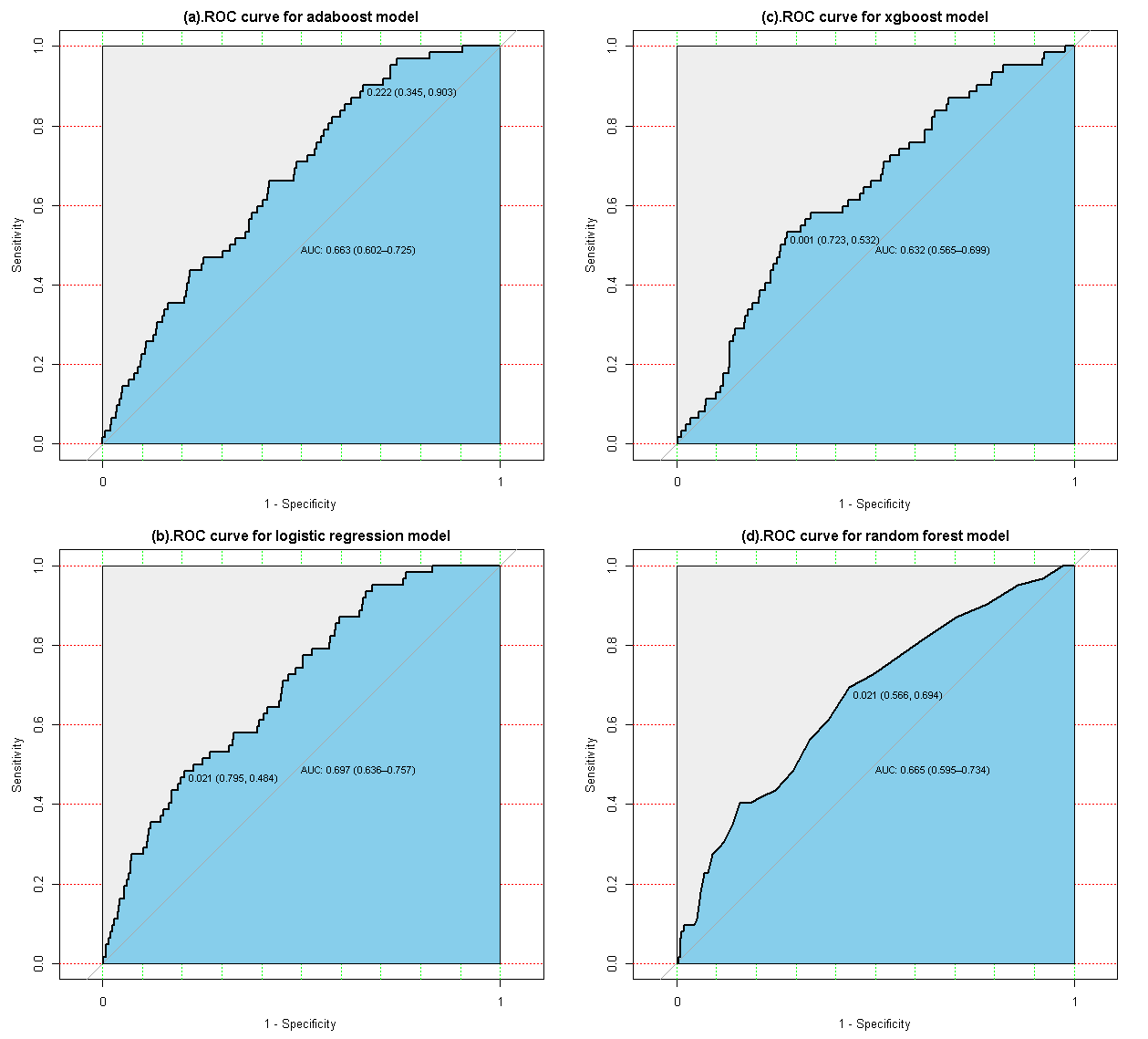   \| Algorithm \| Threshold \| Sensitivity \| Specificity \| PPV \| NPV \| Algorithm \| Threshold \| Sensitivity \| Specificity \| PPV \| NPV \| \| --- \| --- \| --- \| --- \| --- \| --- \| --- \| --- \| --- \| --- \| --- \| --- \| \| adaboost \| 0.196 \| 0.632 \| 0.639 \| 0.018 \| 0.991 \| adaboost \| 0.222 \| 0.903 \| 0.345 \| 0.028 \| 0.994 \| \| logistic regression \| 0.012 \| 0.605 \| 0.678 \| 0.023 \| 0.995 \| logistic regression \| 0.021 \| 0.484 \| 0.795 \| 0.047 \| 0.987 \| \| xgboost \| 0.000 \| 0.605 \| 0.717 \| 0.023 \| 0.997 \| xgboost \| 0.001 \| 0.532 \| 0.723 \| 0.038 \| 0.987 \| \| random forest \| 0.021 \| 0.474 \| 0.796 \| 0.027 \| 0.995 \| random forest \| 0.021 \| 0.694 \| 0.566 \| 0.032 \| 0.989 \|   (C) Using the RF method filtered features to predict conversion to BD within 7 years  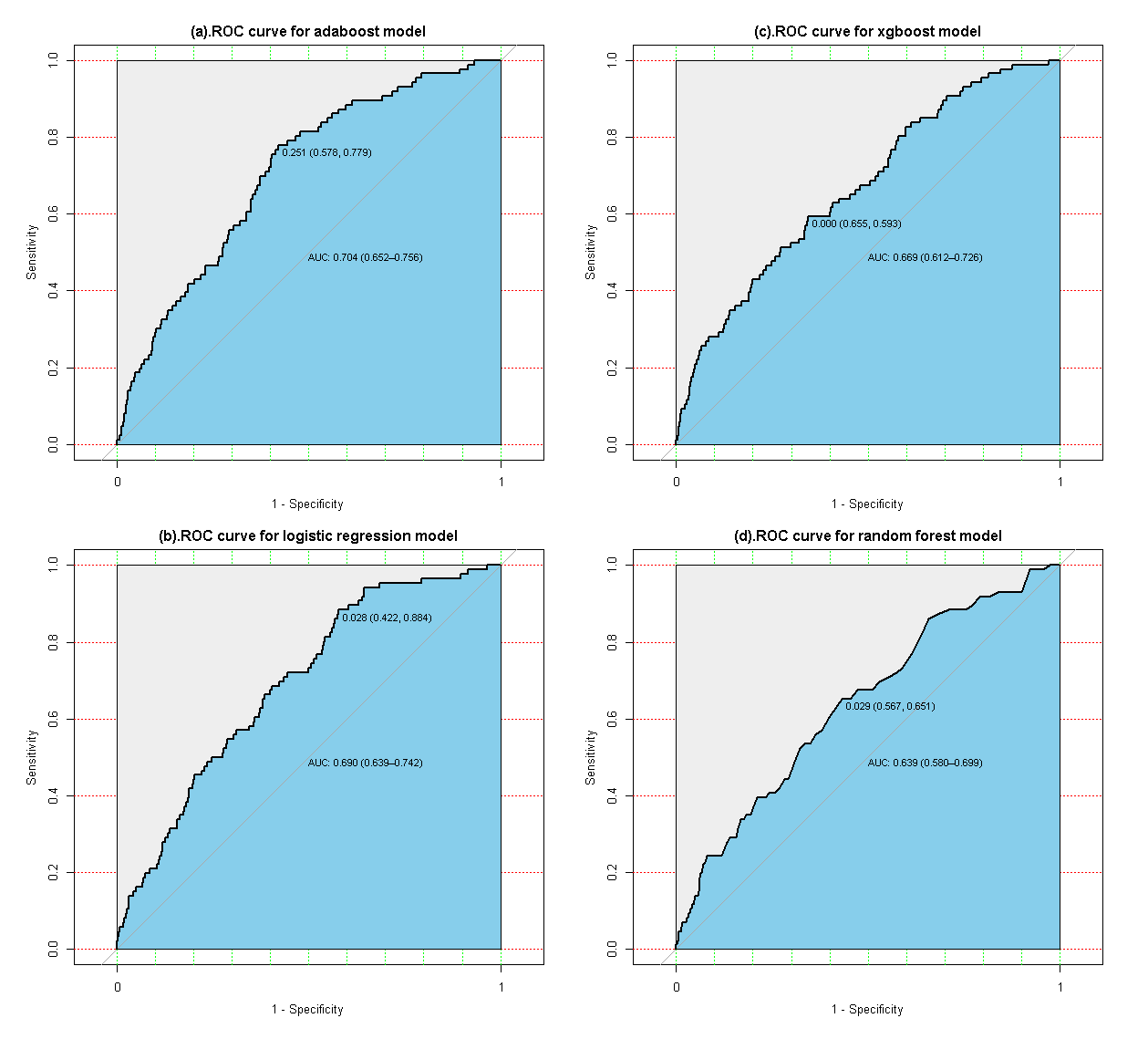   \| Algorithm \| Threshold \| Sensitivity \| Specificity \| PPV \| NPV \| \| --- \| --- \| --- \| --- \| --- \| --- \| \| adaboost \| 0.251 \| 0.779 \| 0.578 \| 0.051 \| 0.989 \| \| logistic regression \| 0.028 \| 0.884 \| 0.422 \| 0.043 \| 0.992 \| \| xgboost \| 0.0002 \| 0.593 \| 0.655 \| 0.048 \| 0.982 \| \| random forest \| 0.029 \| 0.651 \| 0.567 \| 0.042 \| 0.982 \| |
| --- | --- | --- | --- | --- | --- | --- | --- | --- | --- | --- | --- | --- | --- | --- | --- | --- | --- | --- | --- | --- | --- | --- | --- | --- | --- | --- | --- | --- | --- | --- | --- | --- | --- | --- | --- | --- | --- | --- | --- | --- | --- | --- | --- | --- | --- | --- | --- | --- | --- | --- | --- | --- | --- | --- | --- | --- | --- | --- | --- | --- | --- | --- | --- | --- | --- | --- | --- | --- | --- | --- | --- | --- | --- | --- | --- | --- | --- | --- | --- | --- | --- | --- | --- | --- | --- | --- | --- | --- | --- | --- |

Figure S6 The performance of prediction models using the RF method filtered features for the conversion from UD to BD. The area under the receiver operating characteristic (AUC) ROC curve. The tables showed the internal validation performance (i.e., sensitivity, specificity, positive predictive value [PPV], and negative predictive value [NPV]) of prediction models.

| (A) Using the LASSO method filtered features to predict conversion to SCZ within 1 year (B) Using the LASSO method filtered features to predict conversion to SCZ within 3 years  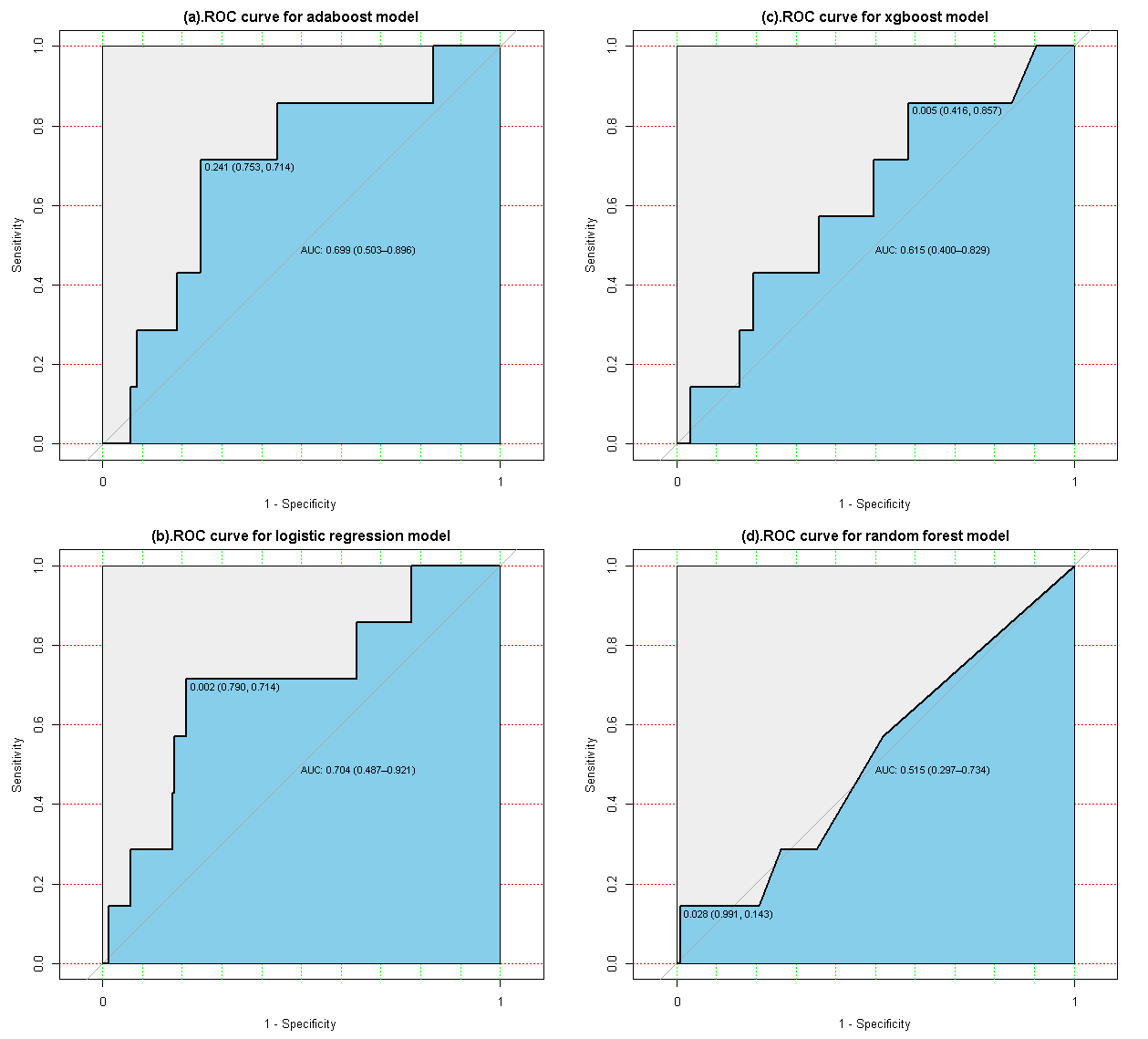 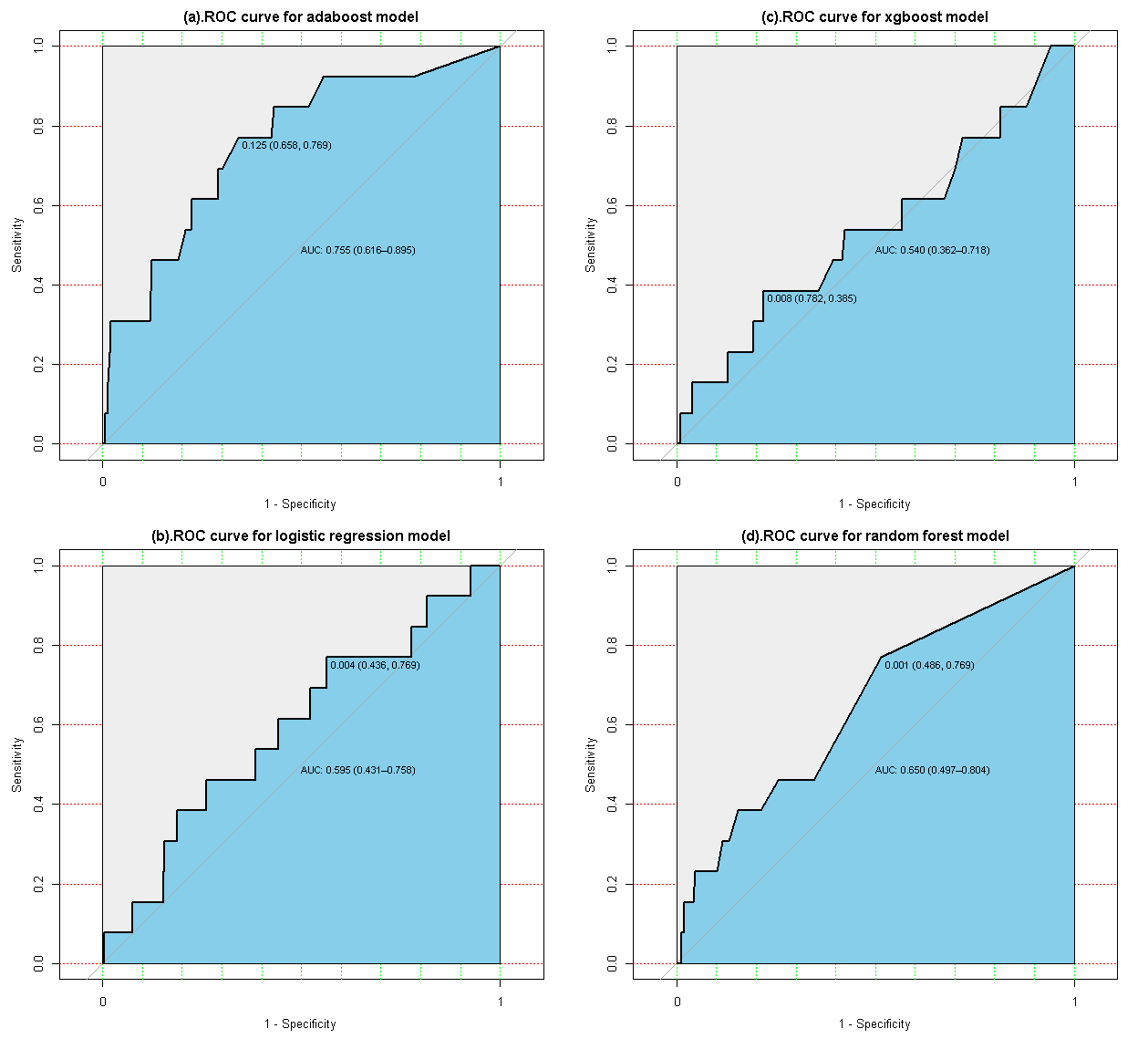   \| Algorithm \| Threshold \| Sensitivity \| Specificity \| PPV \| NPV \| Algorithm \| Threshold \| Sensitivity \| Specificity \| PPV \| NPV \| \| --- \| --- \| --- \| --- \| --- \| --- \| --- \| --- \| --- \| --- \| --- \| --- \| \| adaboost \| 0.241 \| 0.714 \| 0.753 \| 0.007 \| 0.999 \| adaboost \| 0.125 \| 0.769 \| 0.658 \| 0.010 \| 0.998 \| \| logistic regression \| 0.002 \| 0.714 \| 0.790 \| 0.008 \| 0.999 \| logistic regression \| 0.004 \| 0.769 \| 0.436 \| 0.006 \| 0.998 \| \| xgboost \| 0.005 \| 0.857 \| 0.416 \| 0.003 \| 0.999 \| xgboost \| 0.008 \| 0.385 \| 0.782 \| 0.007 \| 0.997 \| \| random forest \| 0.028 \| 0.143 \| 0.991 \| 0.037 \| 0.998 \| random forest \| 0.001 \| 0.769 \| 0.486 \| 0.006 \| 0.998 \|   (C) Using the LASSO method filtered features to predict conversion to SCZ within 7 years  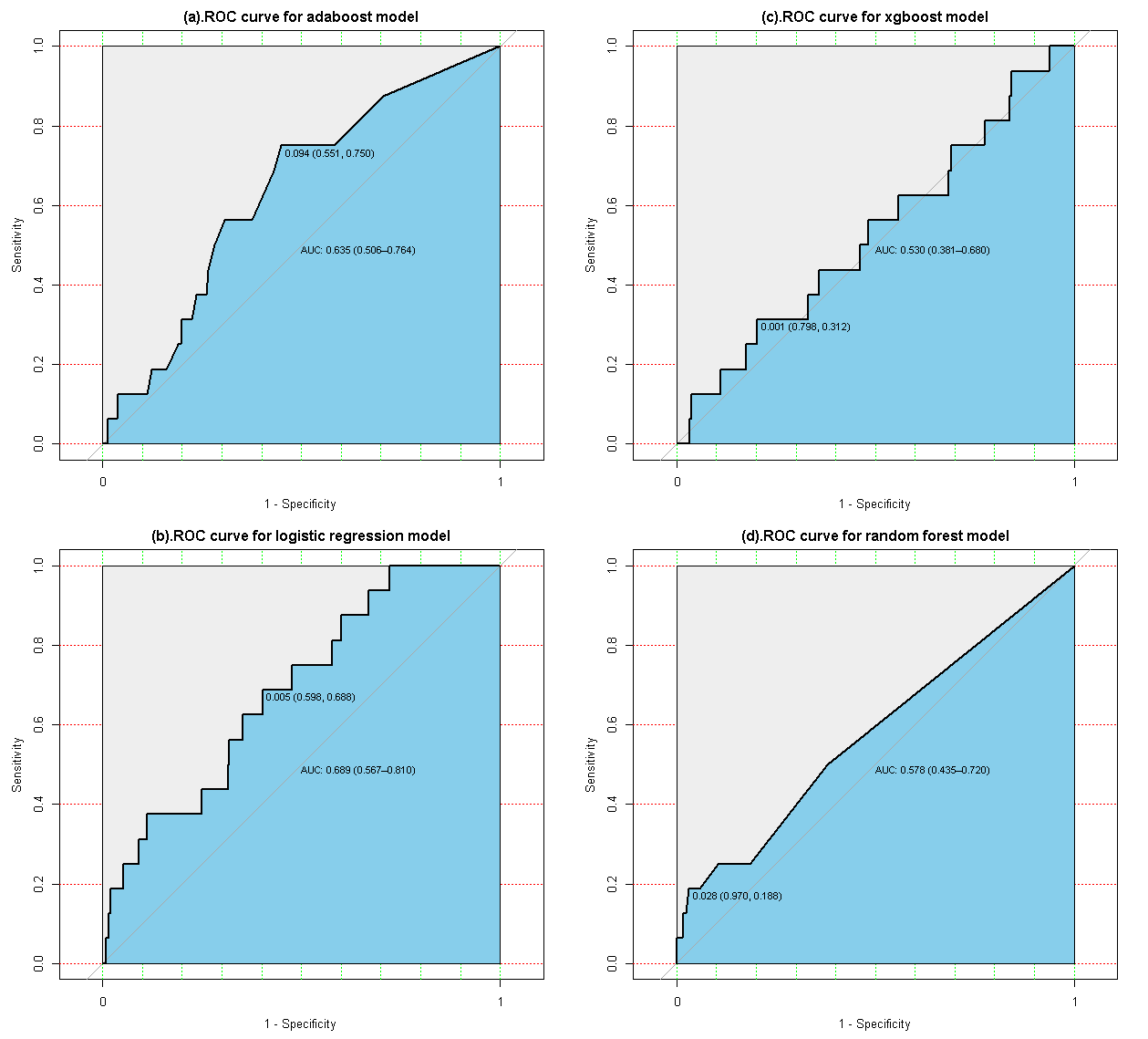   \| Algorithm \| Threshold \| Sensitivity \| Specificity \| PPV \| NPV \| \| --- \| --- \| --- \| --- \| --- \| --- \| \| adaboost \| 0.094 \| 0.750 \| 0.551 \| 0.009 \| 0.998 \| \| logistic regression \| 0.005 \| 0.688 \| 0.598 \| 0.009 \| 0.997 \| \| xgboost \| 0.001 \| 0.312 \| 0.798 \| 0.008 \| 0.995 \| \| random forest \| 0.028 \| 0.188 \| 0.970 \| 0.032 \| 0.996 \| |
| --- | --- | --- | --- | --- | --- | --- | --- | --- | --- | --- | --- | --- | --- | --- | --- | --- | --- | --- | --- | --- | --- | --- | --- | --- | --- | --- | --- | --- | --- | --- | --- | --- | --- | --- | --- | --- | --- | --- | --- | --- | --- | --- | --- | --- | --- | --- | --- | --- | --- | --- | --- | --- | --- | --- | --- | --- | --- | --- | --- | --- | --- | --- | --- | --- | --- | --- | --- | --- | --- | --- | --- | --- | --- | --- | --- | --- | --- | --- | --- | --- | --- | --- | --- | --- | --- | --- | --- | --- | --- | --- |

Figure S7 The performance of prediction models using the LASSO method filtered features for the conversion from UD to SCZ. The area under the receiver operating characteristic (AUC) ROC curve. The tables showed the internal validation performance (i.e., sensitivity, specificity, positive predictive value [PPV], and negative predictive value [NPV]) of prediction models.

| (A) Using the RF method filtered features to predict conversion to SCZ within 1 year (B) Using the RF method filtered features to predict conversion to SCZ within 3 years  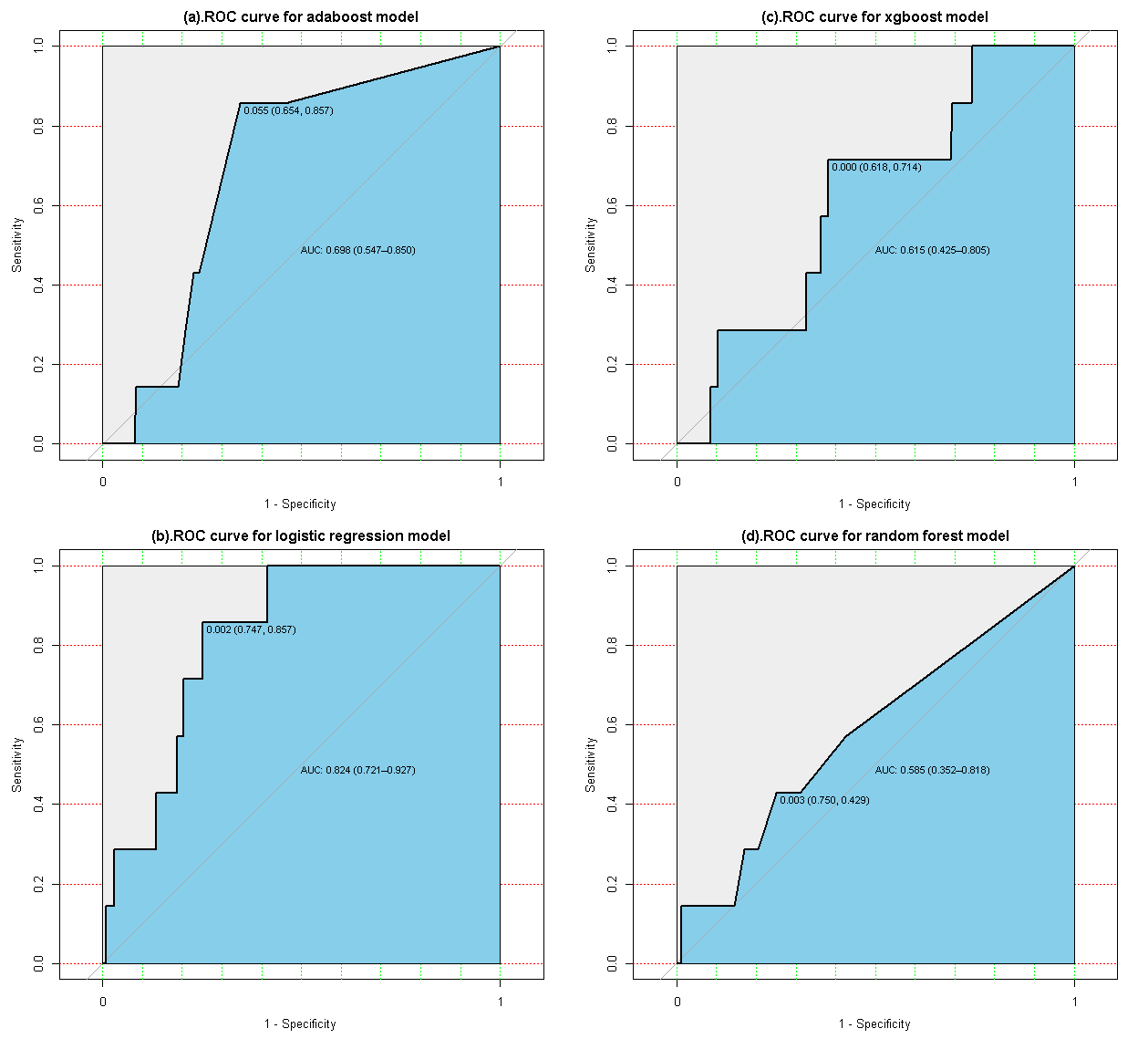 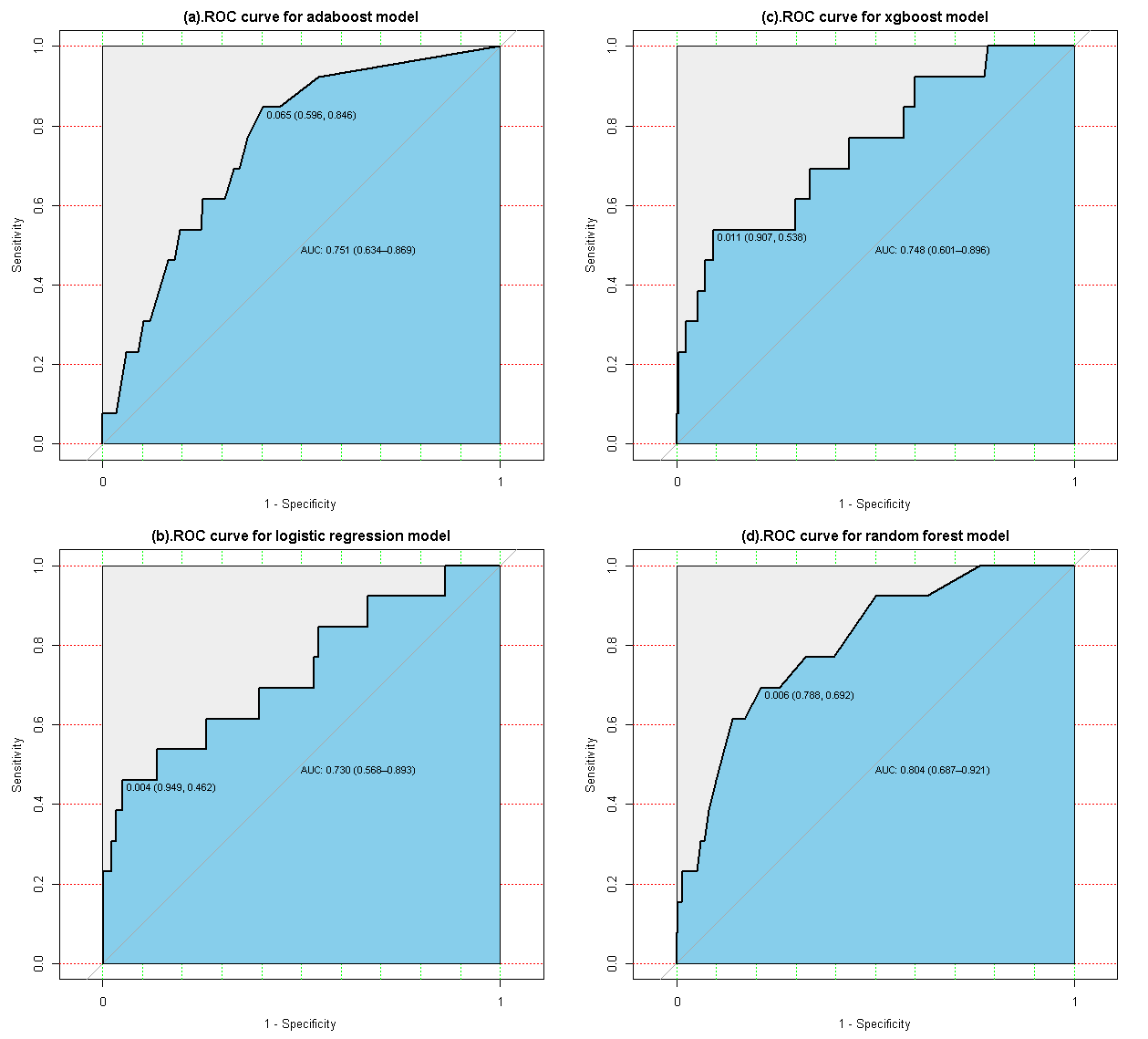   \| Algorithm \| Threshold \| Sensitivity \| Specificity \| PPV \| NPV \| Algorithm \| Threshold \| Sensitivity \| Specificity \| PPV \| NPV \| \| --- \| --- \| --- \| --- \| --- \| --- \| --- \| --- \| --- \| --- \| --- \| --- \| \| adaboost \| 0.055 \| 0.857 \| 0.654 \| 0.006 \| 0.999 \| adaboost \| 0.065 \| 0.846 \| 0.596 \| 0.009 \| 0.999 \| \| logistic regression \| 0.002 \| 0.857 \| 0.747 \| 0.008 \| 1 \| logistic regression \| 0.004 \| 0.462 \| 0.949 \| 0.038 \| 0.996 \| \| xgboost \| 0.000 \| 0.714 \| 0.618 \| 0.004 \| 0.999 \| xgboost \| 0.011 \| 0.538 \| 0.907 \| 0.024 \| 0.998 \| \| random forest \| 0.003 \| 0.429 \| 0.750 \| 0.004 \| 0.998 \| random forest \| 0.006 \| 0.692 \| 0.788 \| 0.014 \| 0.998 \|   (C) Using the RF method filtered features to predict conversion to SCZ within 7 years  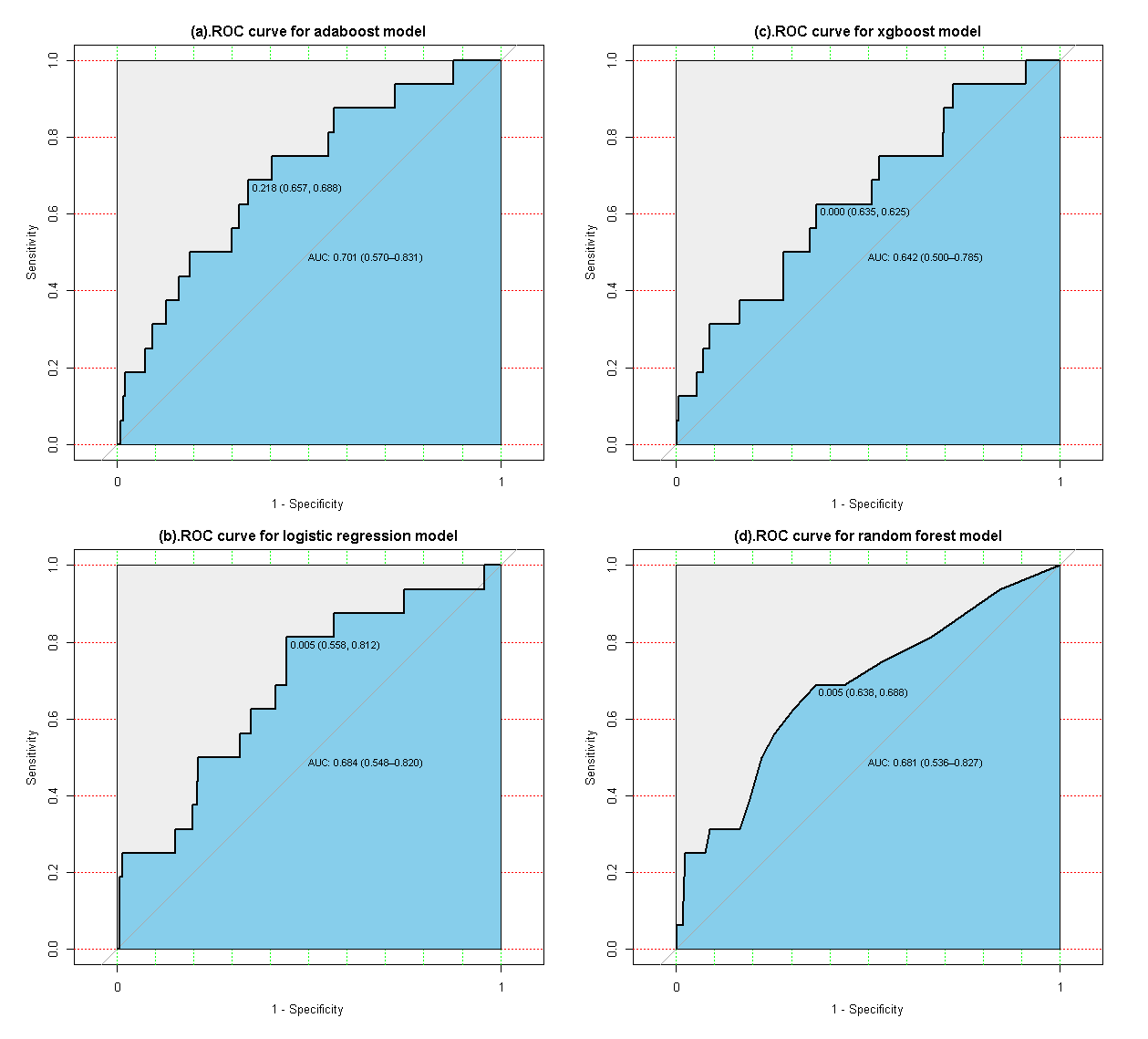   \| Algorithm \| Threshold \| Sensitivity \| Specificity \| PPV \| NPV \| \| --- \| --- \| --- \| --- \| --- \| --- \| \| adaboost \| 0.218 \| 0.688 \| 0.657 \| 0.010 \| 0.997 \| \| logistic regression \| 0.005 \| 0.813 \| 0.558 \| 0.009 \| 0.998 \| \| xgboost \| 0.0001 \| 0.625 \| 0.635 \| 0.009 \| 0.997 \| \| random forest \| 0.005 \| 0.688 \| 0.638 \| 0.010 \| 0.997 \| |
| --- | --- | --- | --- | --- | --- | --- | --- | --- | --- | --- | --- | --- | --- | --- | --- | --- | --- | --- | --- | --- | --- | --- | --- | --- | --- | --- | --- | --- | --- | --- | --- | --- | --- | --- | --- | --- | --- | --- | --- | --- | --- | --- | --- | --- | --- | --- | --- | --- | --- | --- | --- | --- | --- | --- | --- | --- | --- | --- | --- | --- | --- | --- | --- | --- | --- | --- | --- | --- | --- | --- | --- | --- | --- | --- | --- | --- | --- | --- | --- | --- | --- | --- | --- | --- | --- | --- | --- | --- | --- | --- |

Figure S8 The performance of prediction models using the RF method filtered features for the conversion from UD to SCZ. The area under the receiver operating characteristic (AUC) ROC curve. The tables showed the internal validation performance (i.e., sensitivity, specificity, positive predictive value [PPV], and negative predictive value [NPV]) of prediction models.

**Supplementary Information SI5:**

**5.1 Predictors of conversion to BD and SCZ at population-level**

Regarding the importance of features measured using the Break Down method, for short-term stratification of risk for BD conversion from UD (Figure 4(A)), length of stay of this hospitalization treatment for UD, sum of prescribed medical orders, antidepressants, anxiolytics, antipsychotics, β receptor blocker orders, number of prescribed venlafaxine hydrochloride sustained-release capsules, number of prescribed clonazepam tablets and propranolol hydrochloride tablets, whether the patient is admitted to a psychiatric ward, etc., were top ranked in predictive models within 1 year. Of note, markers of greater illness severity (e.g., length of stay, psychiatry versus other specialty care), and more medication prescribed were strongly associated with the short-term risk of BD transition. Notable differences were observed with respect to the medium- and long-term stratification of risk for BD transition. Particularly, some features of lifestyle (e.g., history of frequent medication use), vital signs (e.g., breathing, temperature), and laboratory blood-based markers (e.g., Calcium, Level of urea, Level of alkaline phosphatase and direct bilirubin, Red blood cell volume distribution width CV) were top ranked in predictive models within 2 years and within 7 years. In addition, treatment-related factors (e.g., sum of medical orders, number of orders of modified electroconvulsive therapy, benhexol hydrochloride tablets, escitalopram oxalate tablets, and aspirin enteric-coated tablets) and social factors (age and job status) were also top ranked.

For short-term stratification of risk for SCZ conversion from UD (Figure 4(B)), six features (type of patient discharge is self-discharge, number of orders of clozapine tablets, sulpiride, electroencephalographic (EEG) biofeedback therapy and modified electroconvulsive therapy, and Creatine kinase) were top 10 ranked in models within 1 year, 3 years and 7 years. In addition, features of family history of mental illness, vital signs (e.g., pulse), laboratory blood-based markers (e.g., Direct bilirubin, Lactate dehydrogenase, and Cholesterol), and treatment-related factors (e.g., number of orders of clomipramine hydrochloride tablets and paliperidone sustained-release tablets) were top ranked. Interestingly, top-predictive features included markers of poor prognosis for hospitalization (self-discharge), family history of mental illness, medication and physiotherapy indicated for psychosis, changes in vital signs and biochemical indicators (myocardial enzyme spectrum markers, markers of immune/inflammation, liver biochemistry marker). The detailed information of the included features was shown in Supplementary Information 1.2 Table S1.

**5.2 Predictors of conversion to BD and SCZ at individual-level**

| (A) The importance of top 10 features to predict conversion to BD within 1 year of a UD patient (B) The importance of top 10 features to predict non-conversion to BD within 1 year of a UD patient  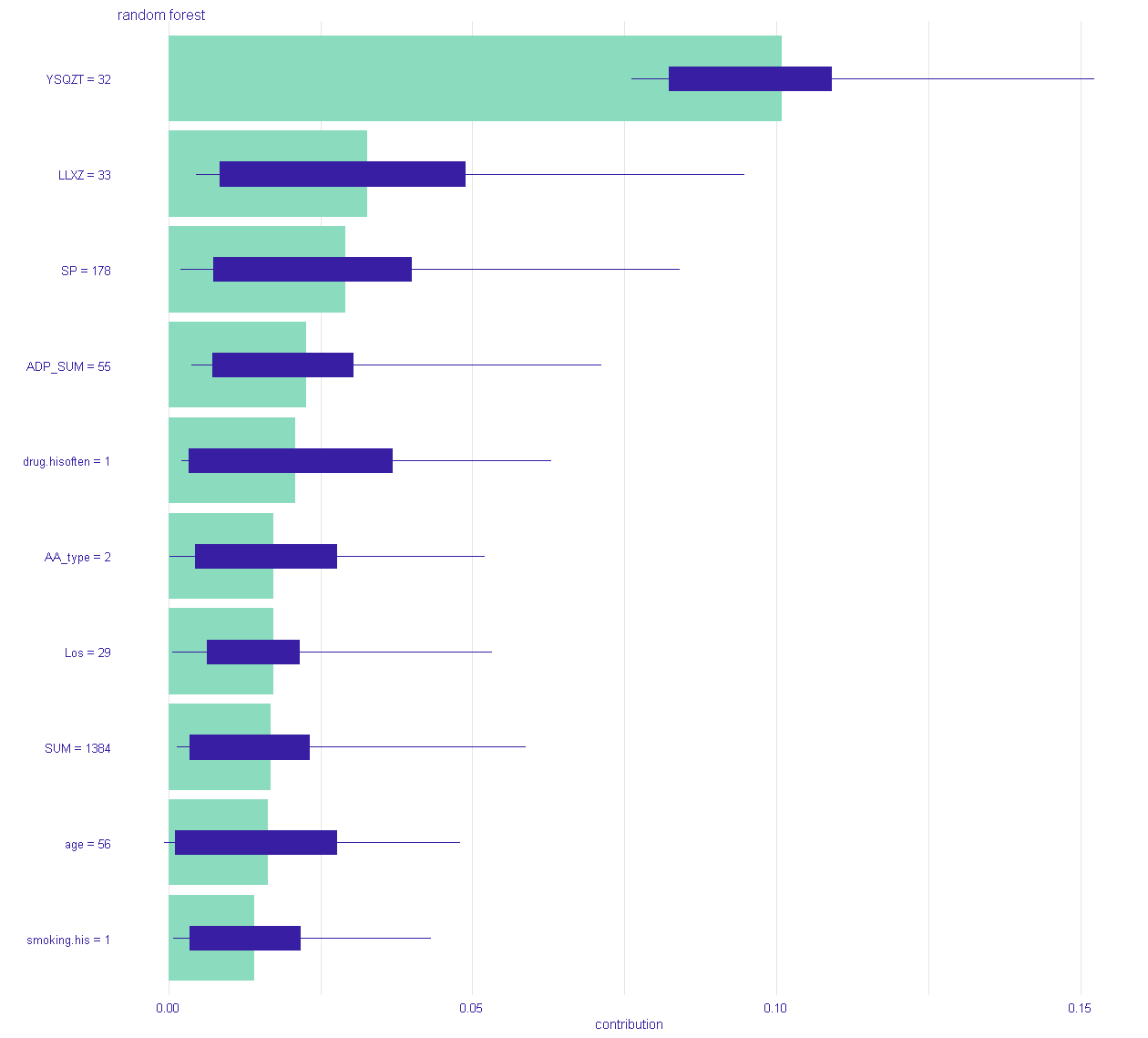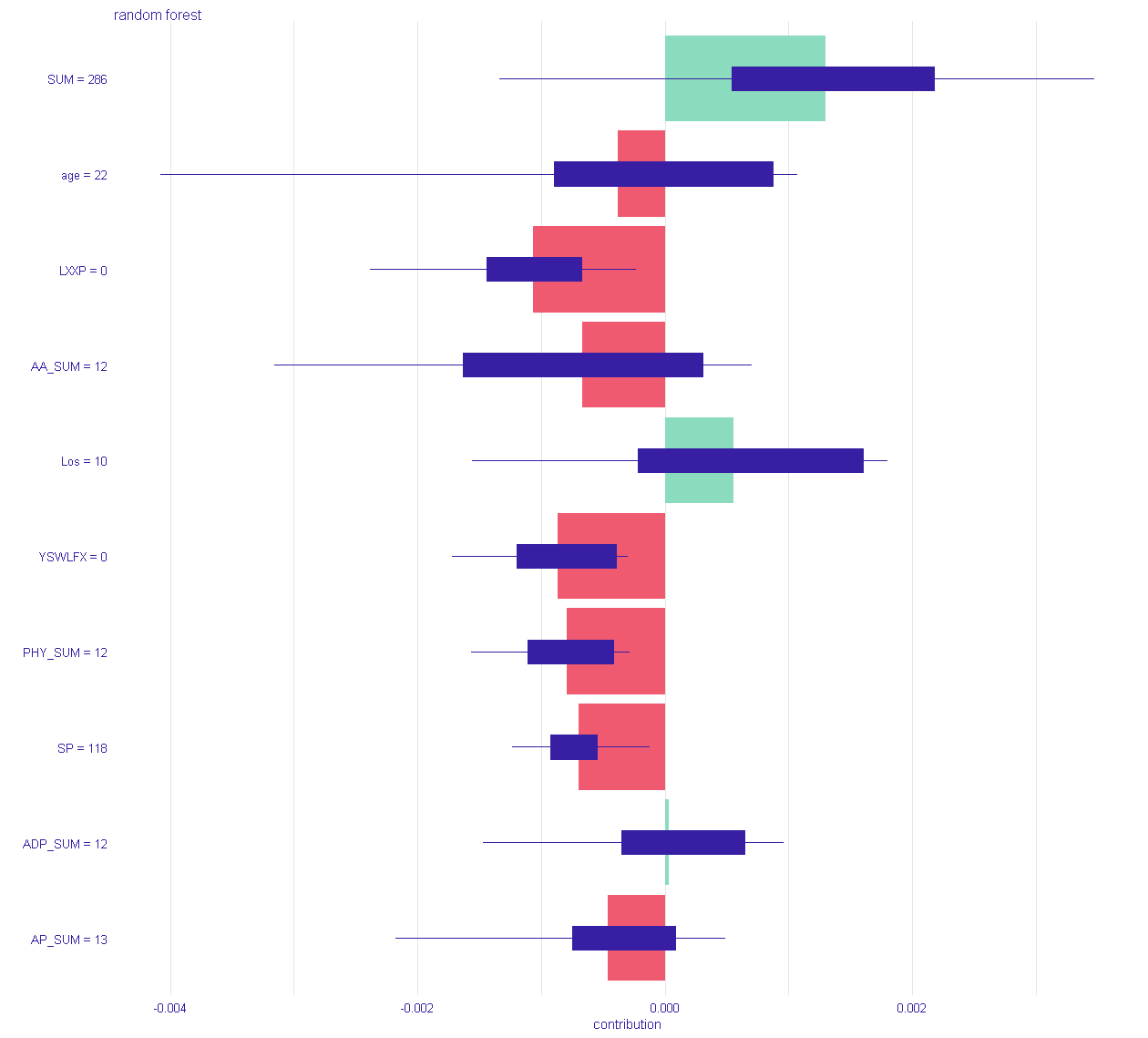  (C) The importance of top 10 features to predict conversion to BD within 2 years of a UD patient (D) The importance of top 10 features to predict non-conversion to BD within 2 years of a UD patient  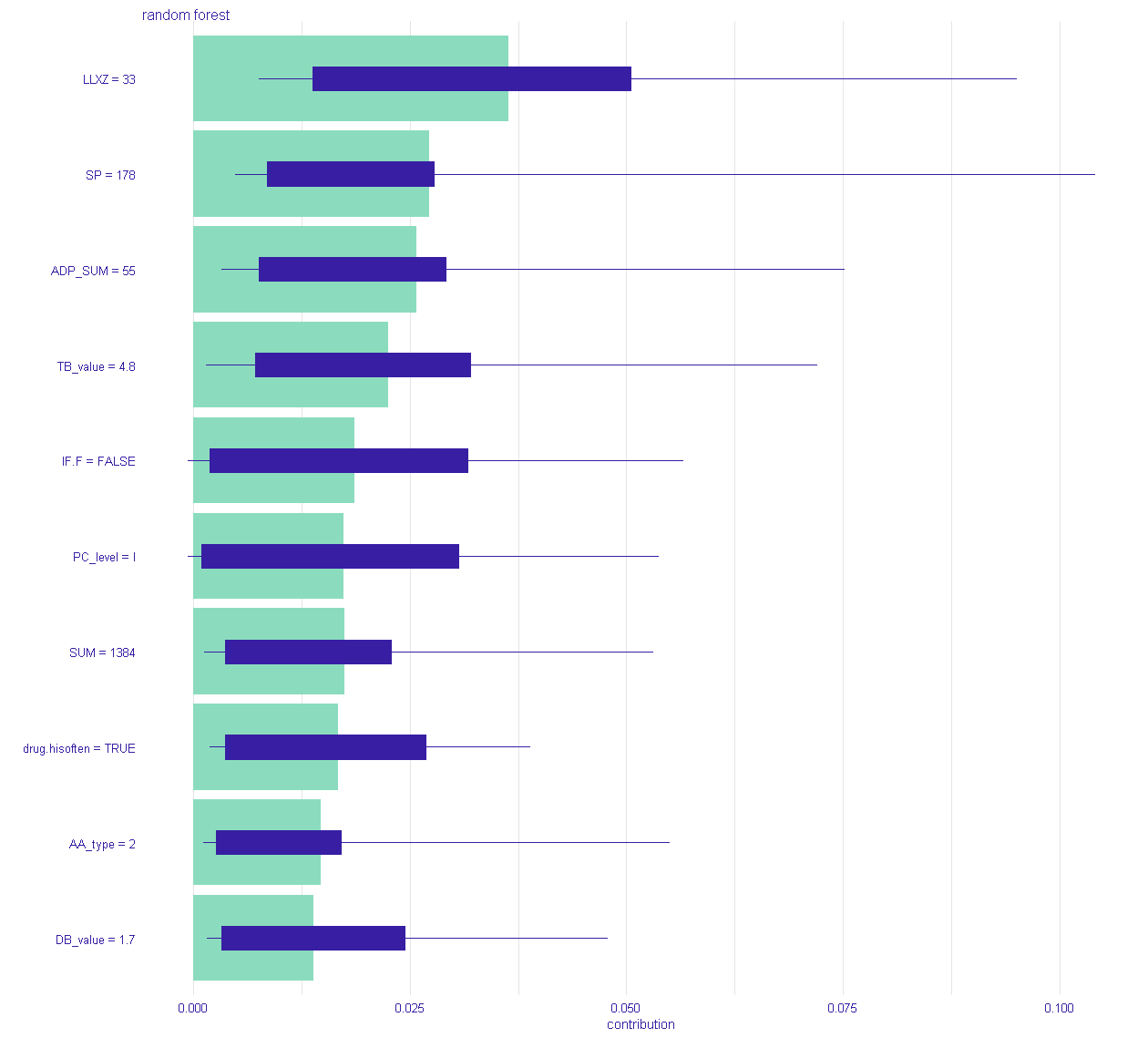 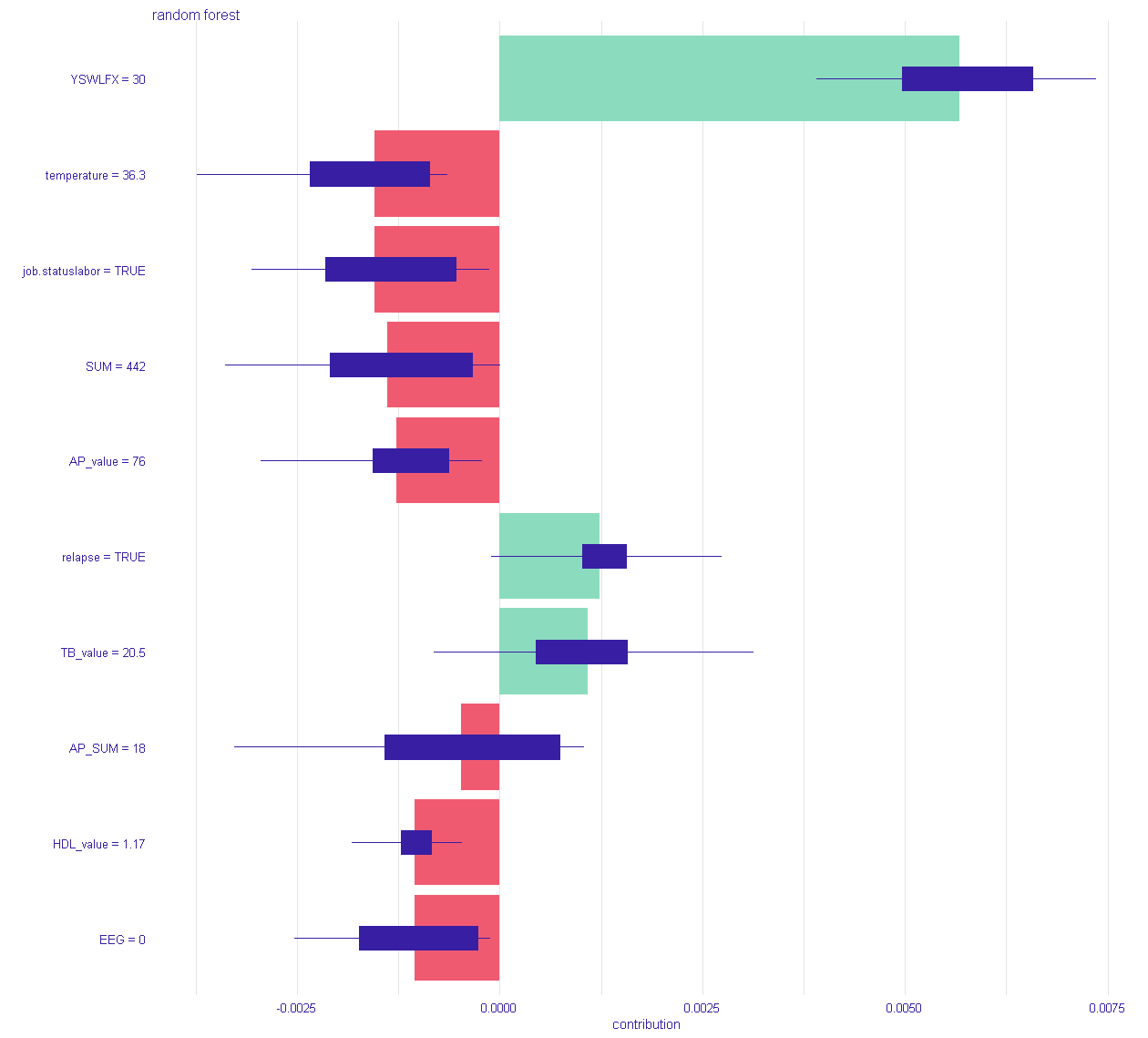  (E) The importance of top 10 features to predict conversion to BD within 7 years of a UD patient (F) The importance of top 10 features to predict non-conversion to BD within 7 years of a UD patient  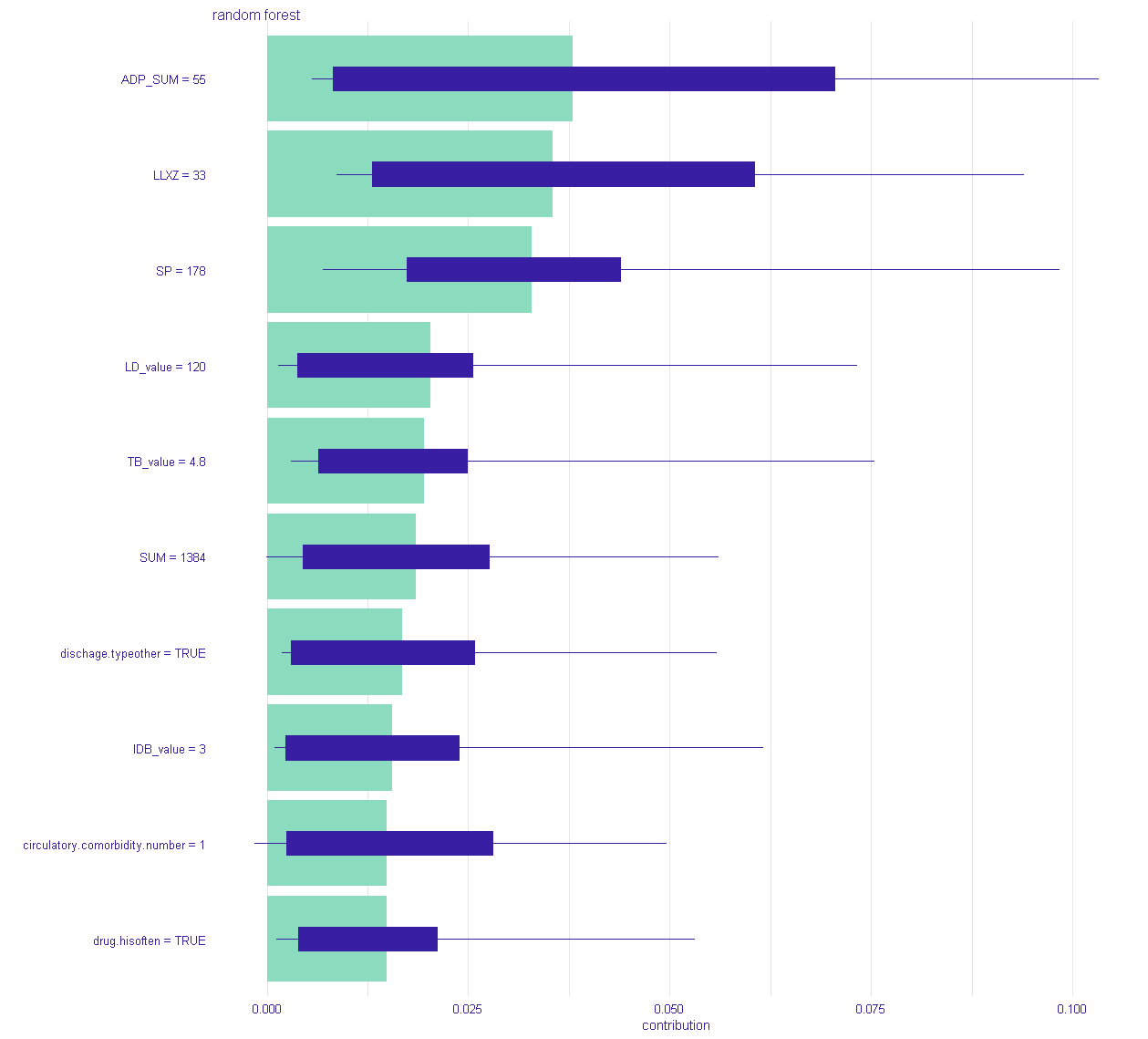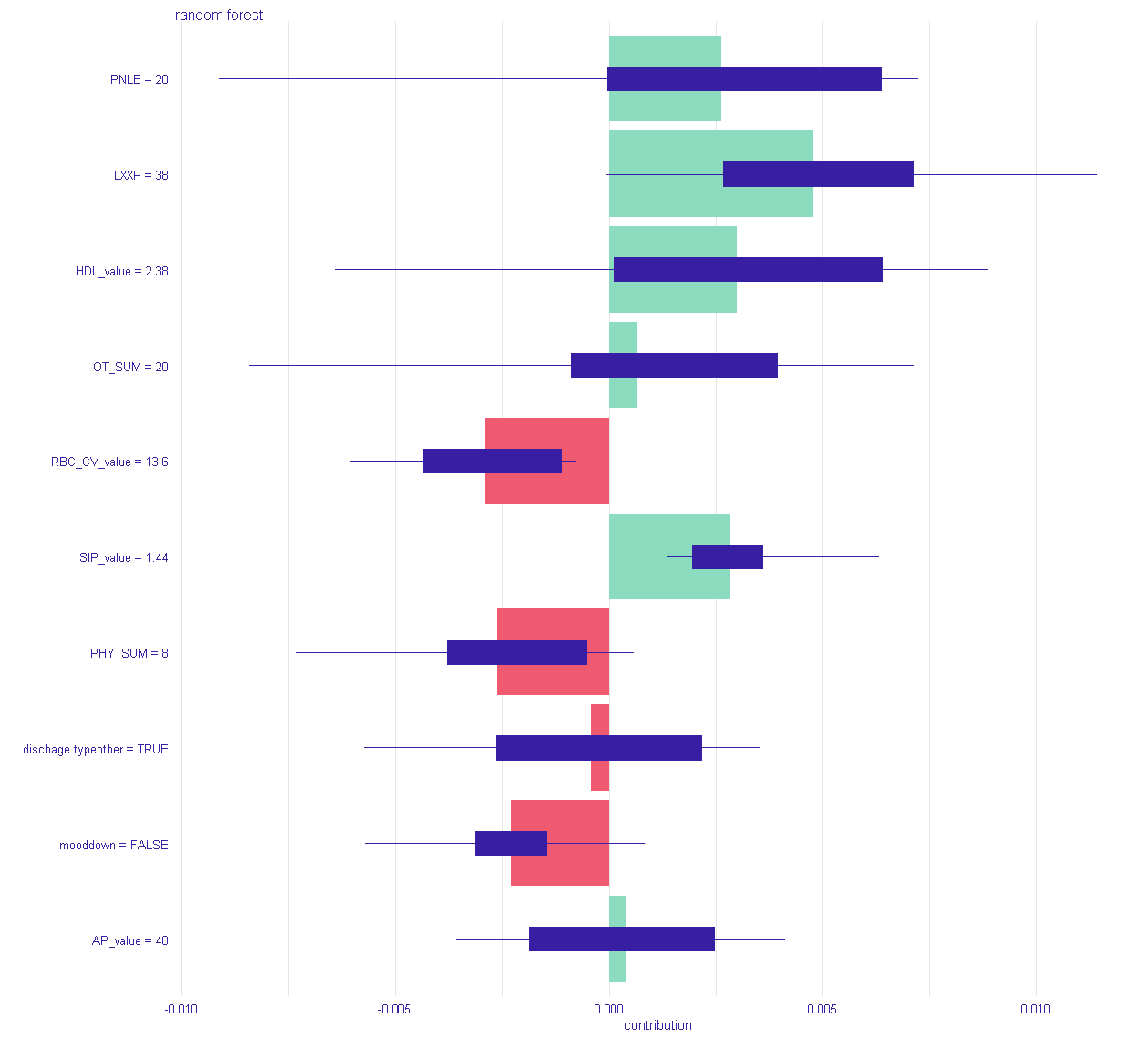 |
| --- |

Figure S9 The comparison of top 10 features to predict conversion and non-conversion from UD to BD within different temporal windows. The green bar represents the positive contribution features, and the red bar represents the negative contribution features.

| (A) The importance of top 10 features to predict conversion to SCZ within 1 year of a UD patient (B) The importance of top 10 features to predict non-conversion to SCZ within 1 year of a UD patient  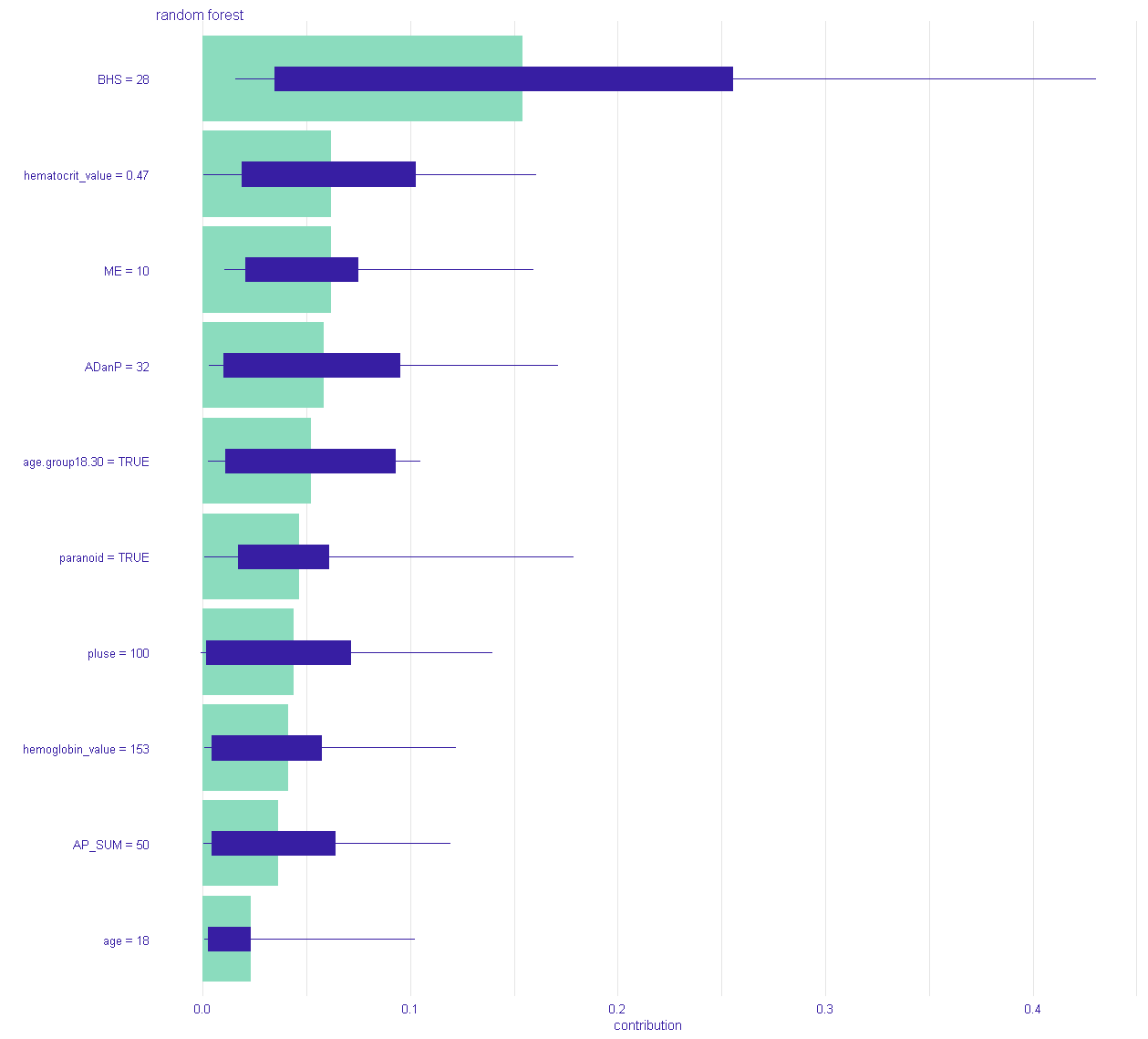 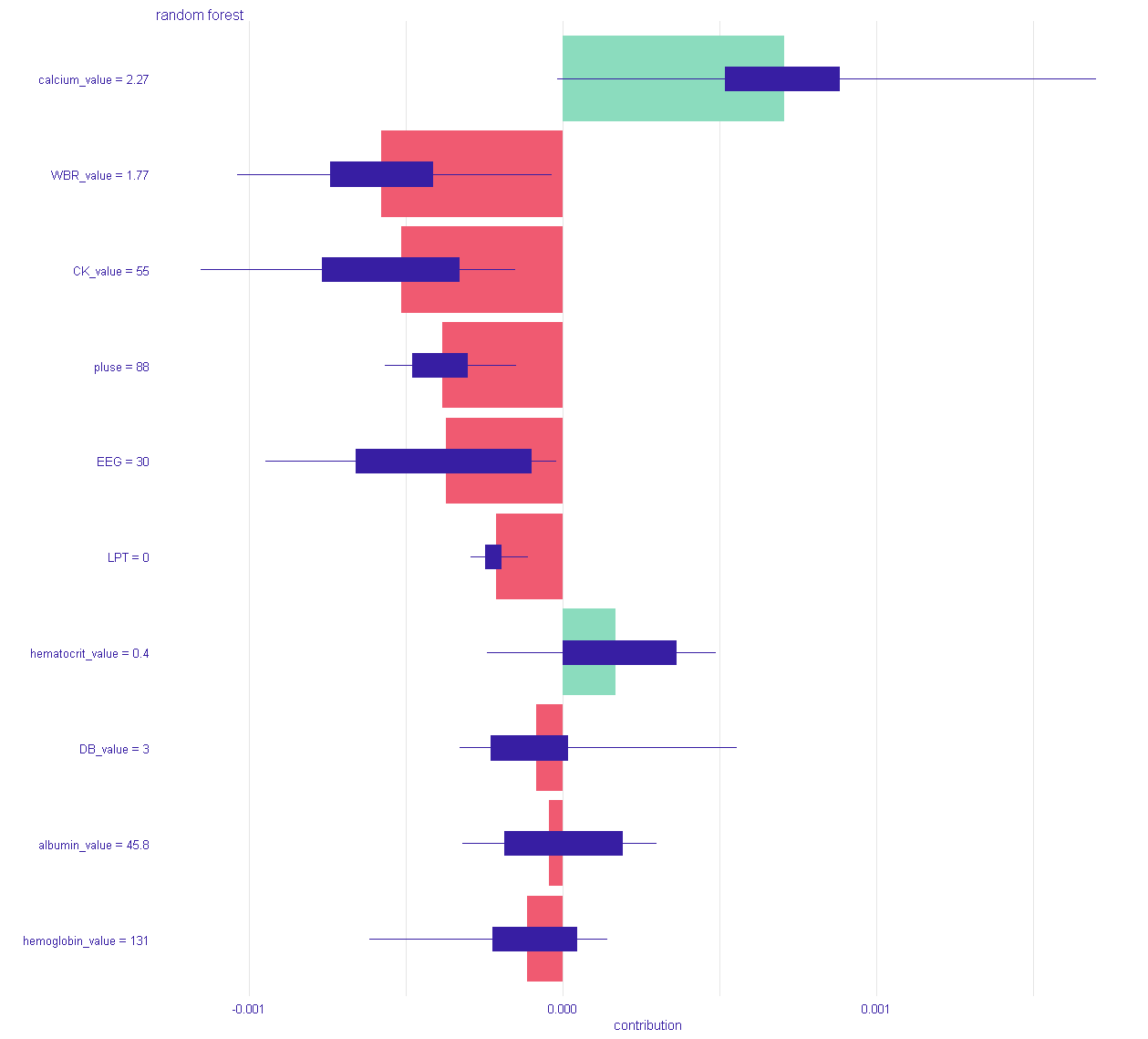  (C) The importance of top 10 features to predict conversion to SCZ within 3 years of a UD patient (D) The importance of top 10 features to predict non-conversion to SCZ within 3 years of a UD patient  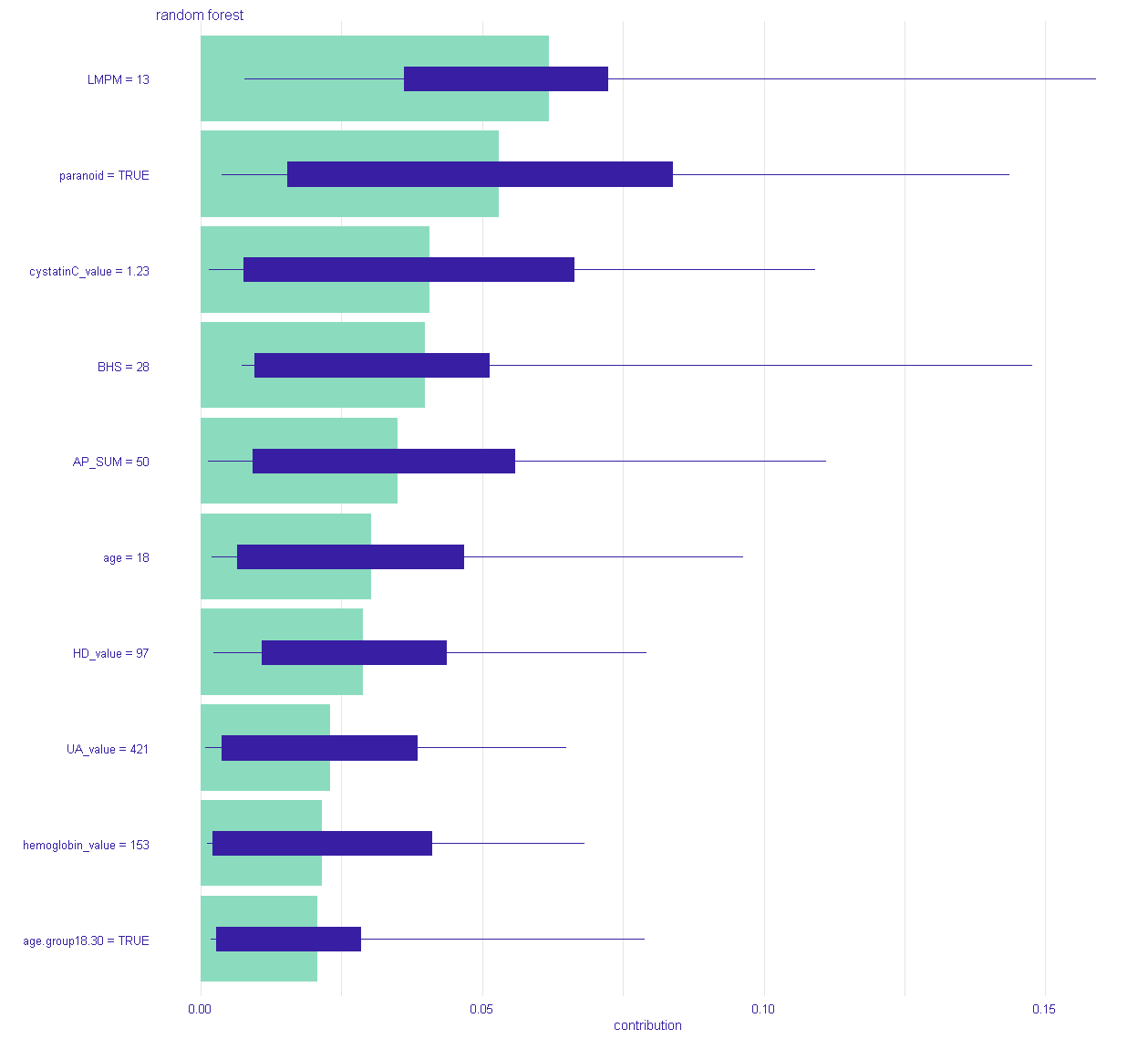 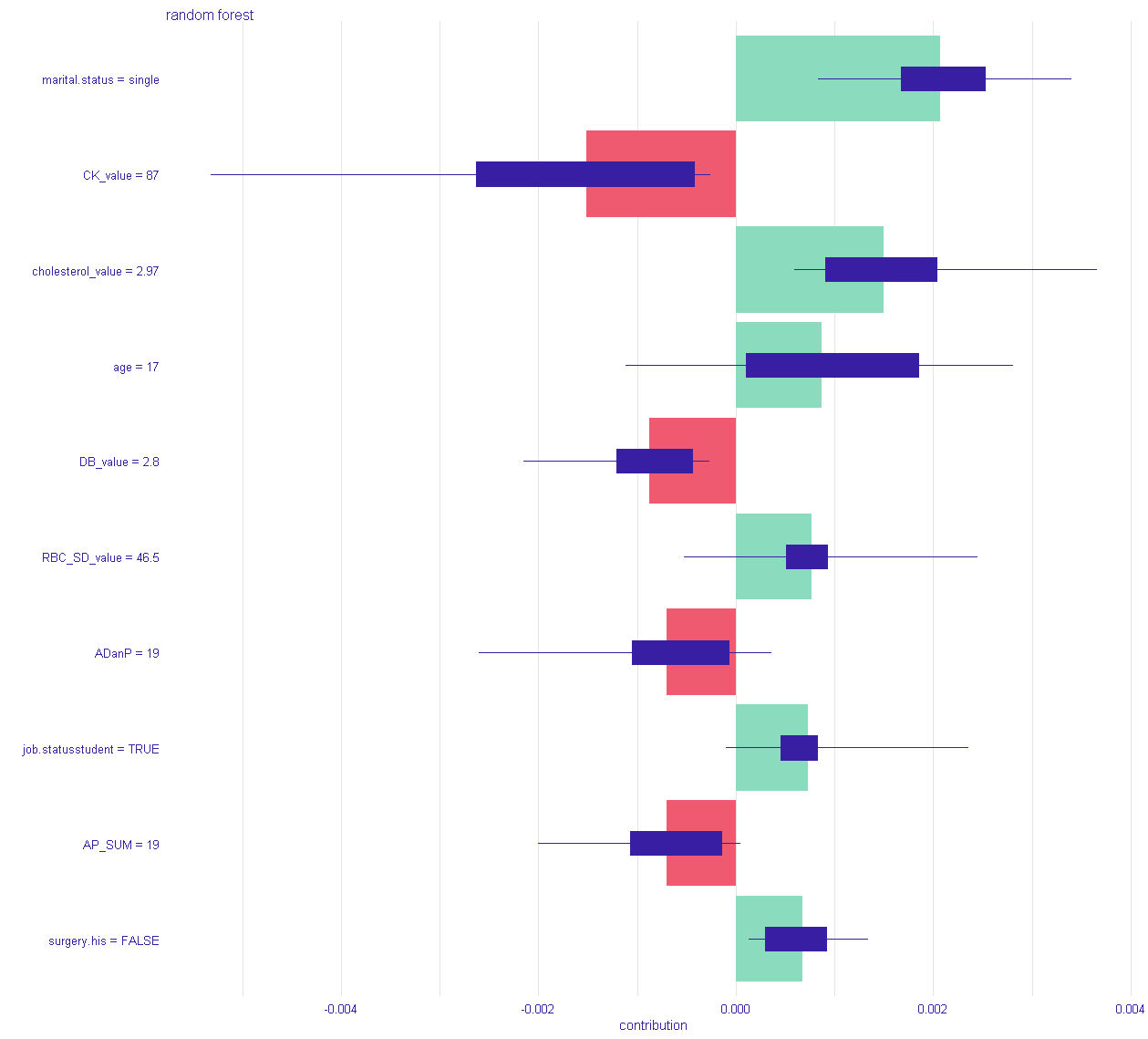    (E) The importance of top 10 features to predict conversion to SCZ within 7 years of a UD patient (F) The importance of top 10 features to predict non-conversion to SCZ within 7 years of a UD patient  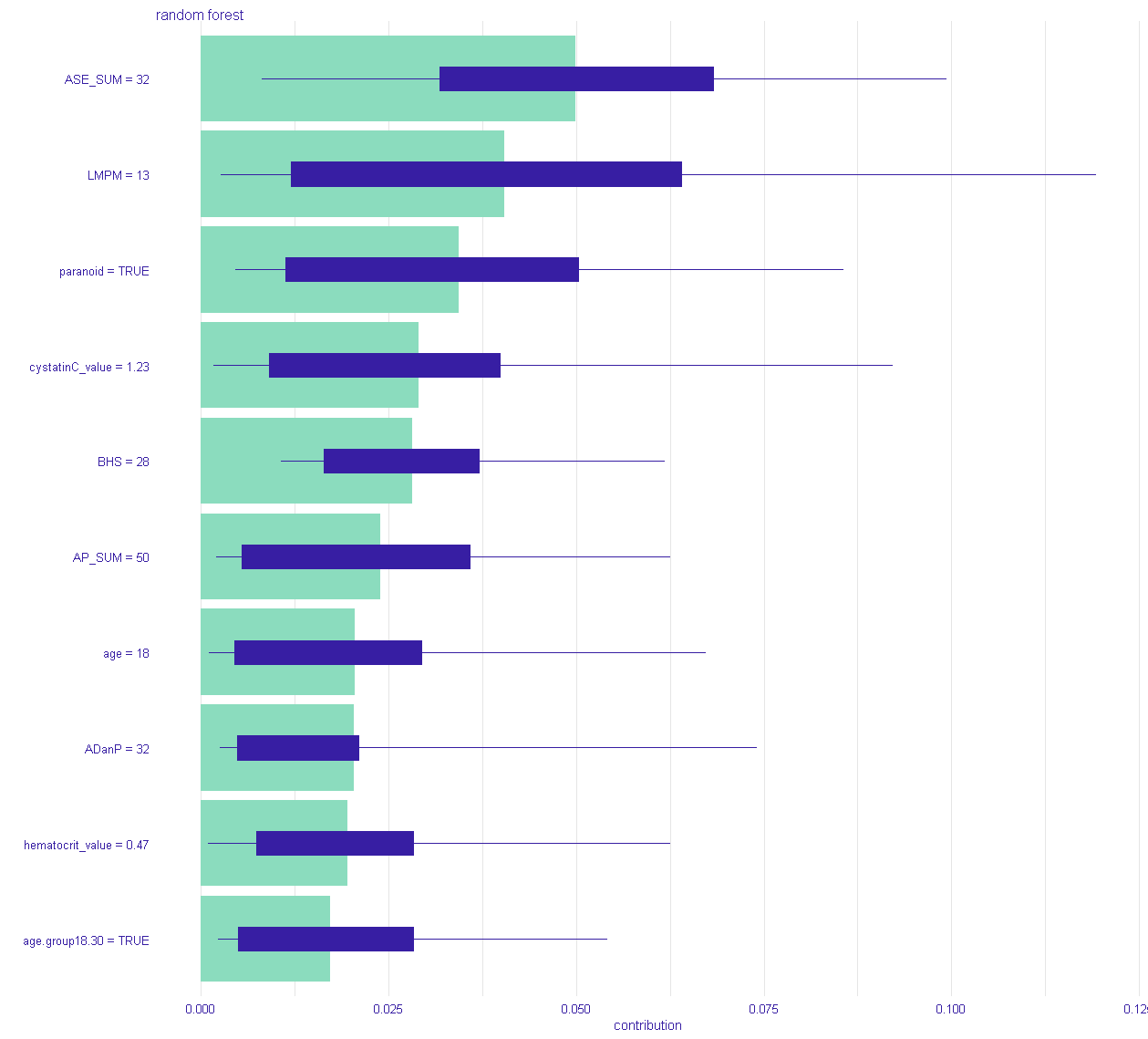 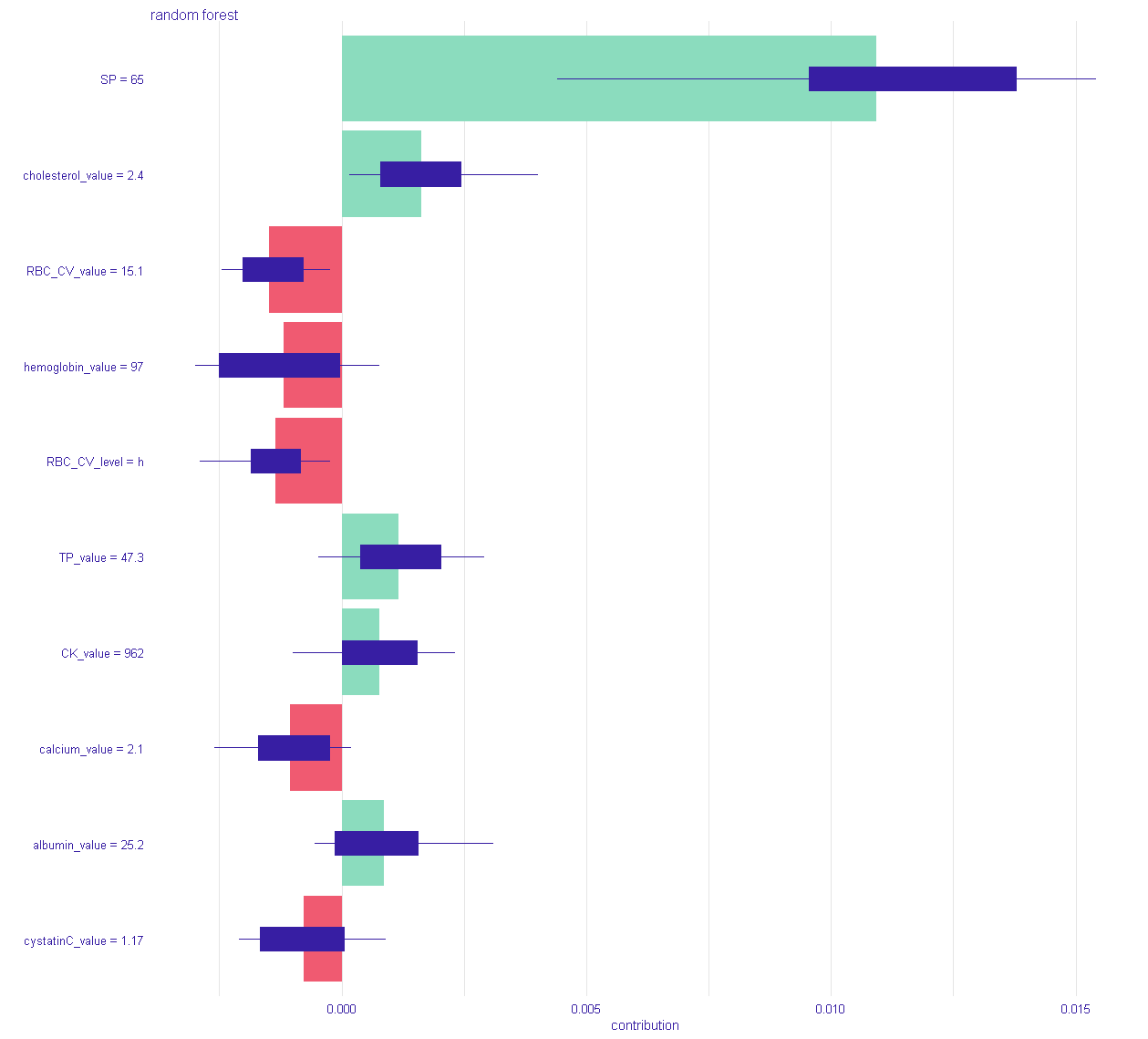 |
| --- |

Figure S10 The comparison of top 10 features to predict conversion and non-conversion from UD to SCZ within different temporal windows. The green bar represents the positive contribution features, and the red bar represents the negative contribution features.
